# A transit-amplifying progenitor with biphasic behavior contributes to epidermal renewal

**DOI:** 10.1242/dev.202389

**Published:** 2024-06-27

**Authors:** Sangeeta Ghuwalewala, Kevin Jiang, Sara Ragi, David Shalloway, Tudorita Tumbar

**Affiliations:** Department of Molecular Biology and Genetics, Cornell University, Ithaca, NY 14850, USA

**Keywords:** Lineage tracing, Stem cells, Skin, Transit-amplifying progenitor, Mathematical modeling, Mouse

## Abstract

Transit-amplifying (TA) cells are progenitors that undergo an amplification phase followed by transition into an extinction phase. A long postulated epidermal TA progenitor with biphasic behavior has not yet been experimentally observed *in vivo*. Here, we identify such a TA population using clonal analysis of Aspm-CreER genetic cell-marking in mice, which uncovers contribution to both homeostasis and injury repair of adult skin. This TA population is more frequently dividing than a Dlx1-CreER-marked long-term self-renewing (e.g. stem cell) population. Newly developed generalized birth-death modeling of long-term lineage tracing data shows that both TA progenitors and stem cells display neutral competition, but only the stem cells display neutral drift. The quantitative evolution of a nascent TA cell and its direct descendants shows that TA progenitors indeed amplify the basal layer before transition and that the homeostatic TA population is mostly in extinction phase. This model will be broadly useful for analyzing progenitors whose behavior changes with their clone age. This work identifies a long-missing class of non-self-renewing biphasic epidermal TA progenitors and has broad implications for understanding tissue renewal mechanisms.

## Teaser

**Highlighted Article:** Understanding transit amplifying progenitor behavior *in-vivo* during epidermal homeostasis opens new roads of investigation in stem cell biology, with broad implications for tissue renewal, aging and cancer.

## INTRODUCTION

Adult epidermal renewal is essential for skin barrier function, which is maintained through homeostasis and injury repair, for animal survival. Tissue renewal is fueled by proliferative cells that divide and differentiate (e.g. progenitors) located in the basal layer (BL). Upon terminal differentiation (TD), these cells move upwards into the suprabasal layers (sBL), shedding from the skin surface (Blanpain and Fuchs, 2009; Flora and Ezhkova, 2020; Hsu and Fuchs, 2022). In regenerative tissues, most progenitors are short-lived non-self-renewing (NSR) populations, whereas stem cells (SCs) are rare long-lived self-renewing (SR) progenitors (Bryder et al., 2006; Claudinot et al., 2005; Furuyama et al., 2011; Tian et al., 2011). Despite decades of work, the lineage organization of epidermal SCs and NSR progenitors is still unclear. The simplest model was based on live imaging (Mesa et al., 2018; Rompolas et al., 2016) and genetic marking driven from ubiquitous promoters Ahr-CreER (Clayton et al., 2007) or Axin-CreER (Lim et al., 2013). It proposed that the BL cells were equivalent and made of a single equipotent progenitor with balanced stochastic choices to self-renew and differentiate while generating neutral drift of observed clone sizes (i.e. number of cells per clone) (Clayton et al., 2007; Lim et al., 2013; Mesa et al., 2018; Piedrafita et al., 2020; Rompolas et al., 2016).

In contrast, genetic marking of BL subsets indicated that epidermal renewal might be complex and contain long-term SR SCs and NSR committed progenitors (Mascre et al., 2012; Sanchez-Danes et al., 2016). In addition, mouse back skin clonal data revealed bias towards even cell-number basal clones, suggesting a pairwise differentiation model that also contradicted the single progenitor model (Aragona et al., 2020). Another highly committed [expressing K10 (also known as Krt10)] and very short-lived BL progenitor divided one or two times before obligatory TD with exit into the sBL (Cockburn et al., 2022). Thus, the BL cells were clearly not all equipotent as suggested (Mesa et al., 2018; Rompolas et al., 2016), and, together with scRNA-seq data analysis, led to a ‘gradualistic’ differentiation model (Cockburn et al., 2022; Joost et al., 2016; Lin et al., 2020).

Furthermore, epidermal BL displays spatial heterogeneity related to location of progenitors near the hair follicle (Roy et al., 2016) or in domains known as scale/interscale in mouse tail and rete ridges/inter-ridges in human skin (Ghuwalewala et al., 2022; Gomez et al., 2013; Sada et al., 2016). Scales and interscales regenerate at different rates and are renewed from two long-term SR progenitors (e.g. SC populations) (Gomez et al., 2013), which we found the Slc1a3-CreER driver (scale enriched) and Dlx1-CreER driver (interscale enriched) mark preferentially (Sada et al., 2016).

Importantly, all SR and NSR progenitors identified to date have constant growth properties during homeostasis. Specifically, for the long-term SR progenitors, such as those rarely marked by Ahr-CreER, Axin-CreER or K14-CreER, choices of SR are constantly balanced with TD (Clayton et al., 2007; Lim et al., 2013; Mascre et al., 2012; Piedrafita et al., 2020; Sanchez-Danes et al., 2016). On the other hand, for NSR progenitors marked by differentiation-specific drivers (Involucrin-CreER or K10-CreER), fate choices are constantly imbalanced towards differentiation (Aragona et al., 2020; Cockburn et al., 2022; Mascre et al., 2012; Sanchez-Danes et al., 2016).

The constant growth properties of epidermal progenitors *in vivo* contrast with the classical behavior of primary cultured untransformed cells, which change their behavior over time in accordance to their cellular ‘age’, displaying two growth phases (e.g. biphasic) (Hayflick and Moorhead, 1961). Initially after plating, the early primary cells undergo an amplification phase, in which they divide repeatedly and increase their numbers. Then, over time, these cells reach a proliferation limit or ‘crisis’ (i.e. the Hayflick limit) when they abruptly transition into an extinction phase and senesce (Hayflick and Moorhead, 1961). In addition, primary cultured human epidermal cells have heterogeneous proliferation limits (e.g. form holoclones, meroclones and paraclones) (Barrandon and Green, 1987) associated with specific transcriptomic profiles (Enzo et al., 2021).

In line with the culture experiments, decades-old predictions from classical tissue kinetics studies suggested a hierarchical lineage model for tissues *in vivo*. In this model, infrequently dividing SCs (identified as label retaining cells, or LRCs, in pulse-chase experiments) divide and generate another SC and a frequently dividing biphasic transit-amplifying (TA) cell. The latter would divide repeatedly to amplify the BL (e.g. become non-LRC) and then terminally differentiate. This hierarchical model had important implications for tissue aging and cancer (Potten, 1974; Potten and Loeffler, 1990; Potten et al., 1982). Notably, our earlier lineage tracing data (Sada et al., 2016) were somewhat inconsistent with this model in that both non-LRC-enriched (Slc1a3-CreER) and LRC-enriched (Dlx1-CreER) contained long-term SR progenitors (e.g. SCs). Nonetheless, biphasic epidermal progenitors – i.e., the postulated TA cells – whose behavior changes with their clone age and are non-LRCs might still exist, even though none has yet been identified *in vivo* by genetic lineage tracing.

Here, we use single cell (sc) transcriptomics, clonal analysis combined with mathematical modeling, and functional wound healing studies and experimentally identify *in vivo* an epidermal NSR TA progenitor with biphasic behavior in mouse tail skin. In addition, by developing a generalization of the previously employed ‘critical birth-death’ model (CBDM) (Blanpain and Simons, 2013; Klein and Simons, 2011), we were able to quantitatively analyze the biphasic variation of their growth properties over time. This work bridges historical tissue kinetics and classical SC-TA-TD theory (Potten, 1974; Potten and Loeffler, 1990; Potten et al., 1982) with modern clonal evolution and live imaging studies (Clayton et al., 2007; Cockburn et al., 2022; Lim et al., 2013; Mascre et al., 2012; Rompolas et al., 2016) and uncovers a long-sought population of actively proliferating epidermal TA cells. This expands our understanding of cell fate decisions in homeostasis and tissue renewal *in vivo* and has broad implications for tissue aging and cancer.

## RESULTS

According to the classical SC-TA cell model (Potten, 1974; Potten et al., 1982; Potten and Loeffler, 1990), a TA cell would display characteristic behaviors that uniquely distinguish them from the SCs. Specifically, TA cells would be BL epidermal progenitors that: (1) proliferate faster than the SCs; (2) are molecularly distinct from the SCs; (3) strongly contribute to BL and epidermal renewal; (4) do not SR in long-term – i.e. are non-SR (NSR); (5) undergo biphasic behavior characterized by amplification in the early phase followed by transition into extinction in the late phase; and (6) increase the BL cell numbers in the early phase and decrease them in the late phase. Here, we provide experimental and modeling evidence attesting to the existence of a BL population in the adult epidermis that fulfills the TA progenitor predictions.

### Aspm marks a distinct BL heterogeneous subset of proliferative progenitors

Anticipating that TA cells may proliferate faster than SCs according to the classical model (Potten, 1974; Potten et al., 1982; Potten and Loeffler, 1990), we previously used H2B-GFP pulse-chase in transgenic mice (Tumbar et al., 2004) to isolate LRC and non-LRC BL cellular subsets (Sada et al., 2016). We then used the non-LRC genetic markers we had identified (Sada et al., 2016; Ghuwalewala et al., 2022) to search for a TA population. Following pilot studies with Slc1a3-CreER and Aspm-CreER marked populations, we found that the Slc1a3-CreER-marked population contained long-lived SR cells (Sada et al., 2016; Ghuwalewala et al., 2022; S.G., S. Lee and T.T., unpublished). We therefore focused here on Aspm as another a potential non-LRC TA cell marker. We used the LRC BL marker Dlx1, which contained long-term SR progenitors (Sada et al., 2016), as a control population.

**Fig. 1.**
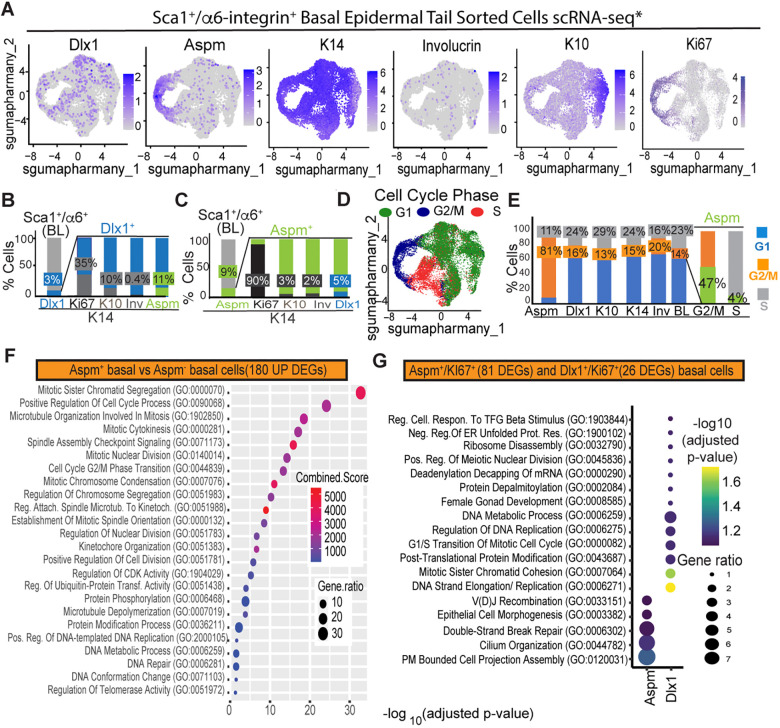
**Aspm is expressed in a distinct subset of highly proliferative basal cells.** (A) scRNA-seq feature plots of Sca1^+^/α6-integrin^+^ basal layer (BL) cells sorted from mouse tail skin at PD52. *Data extracted from Ghuwalewala et al., 2022 scRNA-seq database. Shades of blue show expression levels. Dlx1 and Aspm mark the BL subsets employed in this study, K14 marks the BL, K10 and Involucrin mark differentiating cells, and Ki67 marks proliferative cells (Hsu and Fuchs, 2022). (B) The scRNA-seq BL data in A were analyzed to determine the fractions of Dlx1^+^ cells that co-expressed the indicated genes. (C) As in B, except that the Aspm^+^ cells were analyzed. (D) Cell cycle phase PCA analysis of BL cells. (E) Fractions of distinct BL populations in the cell cycle phases. (F,G) Differentially expressed genes (DEGs) that were upregulated (UP) in the indicated cells as analyzed using GO Ontology (2023) identifiers. DEGs in G were extracted by comparing each population with all BL Ki67^+^ cells. Enrichr combined scores (indicated by color) (Edward et al., 2013) and gene expression ratio (indicated by ellipse size) are shown. The *x*-axis in F shows the negative log_10_ of the *P*-value adjusted for multiple testing (Benjamini-Hochberg correction).

To characterize the Aspm^+^ and Dlx1^+^ basal epidermal subsets in more depth, we analyzed gene expression in our sc transcriptomics (RNA-seq) dataset generated using 10x Genomics Illumina RNA-seq of Sca1^+^/α6-integrin BL cells sorted from mouse tail skin (Ghuwalewala et al., 2022) (Fig. 1A). Aspm and Dlx1 mRNAs were expressed in different subsets of ∼3%-9% BL cells and had little overlap with each other or with differentiating BL cells that are K10^high^/K14^+^ (Cockburn et al., 2022) or Involucrin^+^/K14^+^ (Mascre et al., 2012)(Fig. 1B,C). Moreover, Aspm^+^, but not Dlx1^+^, cells were highly enriched (>90%) in proliferative Ki67^+^ cells (Fig. 1A-C) and were primarily in S or G2/M phase (Fig. 1D,E). This suggested a strong proliferative status of the Aspm^+^ cells when compared with Dlx1^+^ and with other BL subsets (Fig. 1E). However, not all BL proliferative cells are Aspm^+^, with ∼47% of all G2/M and ∼4% of S-phase basal cells expressing Aspm (Fig. 1E).

Aspm mRNA expression in a subset of proliferative BL cells was also found in scRNA-seq data from human skin (Fig. S1A). ScRNA-seq primarily detects high-expressing cells due to well-known sensitivity issues inherent to this method, therefore we can expect the 9% Aspm^+^ cells to be an underestimate. As predicted, antibody staining of tissue sections revealed broader and heterogeneous expression in many BL cells. This occurred in both mouse tail scale/interscale and back skin, and in human skin rete ridges/inter-ridges (Fig. S1B-E) and (Ghuwalewala et al., 2022). These data demonstrate that the non-LRC marker Aspm is widely expressed in epidermal domains and is found at variable levels in the BL of skin tissues.

To further understand the molecular identity of the Aspm^+^ cells from scRNA-seq, we dissected pathways from our gene expression dataset of 14,833 tail BL epidermal cells (Ghuwalewala et al., 2022). Compared with BL cells, the Aspm^+^ BL cells showed increased proliferative features such as the ability to regulate cell cycle, spindle organization, kinetochore assembly and DNA repair (Fig. 1F). Importantly, comparing the Aspm^+^/Ki67^+^ or the Dlx1^+^/Ki67^+^ molecular characteristics suggested that they are distinctive proliferative basal populations (Fig. 1G). Furthermore, a small fraction (10%) of basal Aspm^+^ were Ki67^−^ and were enriched in intermediate filament organization and epidermal differentiation pathways when compared with Aspm^+^/Ki67^+^ cells (Fig. S1F,G).

To examine lineage trajectory predictions for the BL epidermal cells, we regressed out cell cycle genes and analyzed clustering of the scRNA of 14,833 tail BL cells using Monocle 3 (Fig. S2A,B). After regression, many Aspm^+^ G2/M cells re-clustered with other undifferentiated BL clusters (Fig. S2C); of the three Aspm^+^-enriched clusters (#4, #5 and #7), cluster 4 was predicted to contain the root of epidermal lineage hierarchy (Fig. S2B,C). On the other hand, cluster 5 contained cells with the highest Aspm expression level (Fig. S2C), and Aspm^+^ cells in the three clusters differentially expressed genes (DEGs) (Fig. S2D). Taken together, the data suggest that the Aspm^+^ basal subset marks a heterogeneous population of putative epidermal progenitors with high proliferative status that may contribute to epidermis renewal.

### Genetic lineage tracing demonstrates that Aspm-CreER-marked cells contribute to long-term epidermis homeostasis and injury repair

**Fig. 2.**
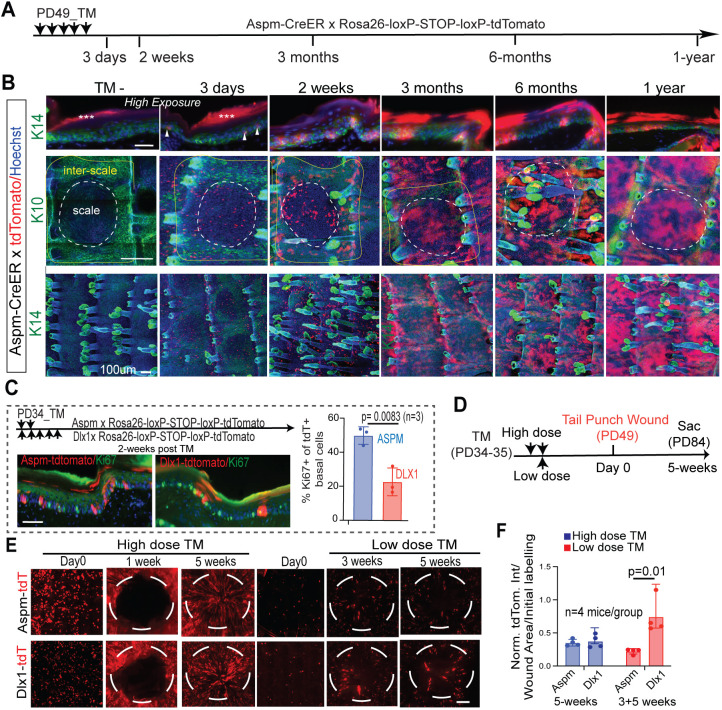
**Aspm-CreER genetic marking of basal cells demonstrates contribution of the Aspm^+^ progenitors to tail epidermis homeostasis and wound repair.** (A) Schematic of high tamoxifen (TM) dose lineage tracing in Aspm-CreER mice. Each arrow indicates one injection/day of 100 µg/g body weight TM. (B) Images of immunofluorescent (IF)-stained tail skin in tissue sections (upper panel) and whole mount (lower panel) from mice in A show robust epidermal tdTomato labeling. No leaky tdTomato expression was observed in TM negative (TM^−^) control mice analyzed at the beginning of the chase (left panel) or at subsequent time points (Fig. S3A). Asterisks indicate autofluorescence from the cornified envelope seen in images taken at high exposure. Arrowheads indicate tdTomato^+^ basal cells. Dashed line encircles scales (white) and interscales (yellow). Starting at 2 weeks, images were taken at low exposure to avoid saturation due to the bright accumulation of tdTomato signal. (C) Schematic of high dose TM injections for Aspm- and Dlx1-CreER lineage traced mice (top) used for Ki67 IF staining (bottom). Each arrow indicates one injection/day. Quantification of images like those in C (right panel). Error bars represent s.d. and *P*-values were calculated using a two-tailed unpaired Student's *t*-test from *n*=3 mice and 5-8 images per mouse. (D) Schematic of the tail punch wound experiment. Each arrow indicates one injection/day. (E) Top-view images of tail wounds showing tracks of the tdTomato signal from the Aspm- and Dlx1-CreER lineages at indicated times after injury. (F) TdTomato signal intensities within the wounded areas shown in E (broken outlines) relative to the intensities at day 0. *N*=4 mice/group, with low TM 3 week (*n*=2) and 5 week (*n*=2) mice combined into one group. *P*-values were calculated by a two-tailed unpaired Student's *t*-test. Scale bars: 100 µm (B); 50 µm (C); 200 µm (E).

To test experimentally whether Aspm^+^ basal cells act as progenitors in the epidermal lineage, as predicted by our scRNA-seq data, we employed mice carrying the Aspm-CreER genetic driver (Marinaro et al., 2011). We crossed these mice to the Rosa26-loxP-STOP-loxP-tdTomato reporter mice and performed lineage tracing with two or five injections of high dose tamoxifen (TM) (see Materials and Methods) to efficiently mark Aspm-expressing BL cells (Fig. 2A; Fig. S3A,B). Whole-mount staining and microscopy imaging of tail epidermis revealed detectable tdTomato^+^ BL cells as early as 3 days post-induction (Fig. 2B). The tdTomato^+^ signal substantially increased in the TM-injected mice over time, generating bright basal and suprabasal patches of labeled cells in both scale and interscale (Fig. 2B). The Aspm-CreER induced signal strongly persisted in the skin in both basal and suprabasal layers up to 1 year of chase (Fig. 2B and Fig. S3A, top panels). Furthermore, the tdTomato^+^ labeling showed little overlap with K10 basal signal but overlapped strongly with suprabasal K10^+^ cells (Fig. S3B). No leaky expression of tdTomato due to spurious CreER activation without TM was observed in CreER/tdTomato-bearing mice (Fig. 2B, left panels; Fig. S3A, lower panels). To compare the proliferative status of the Aspm-CreER-marked progenitors with that of another basal progenitor, we employed the Dlx1-CreER carrying mice as control (Sada et al., 2016). Ki67 staining of the Aspm- and Dlx1-CreER-marked tdTomato^+^ cells showed that the Aspm-marked cells are more proliferative than the Dlx1-marked ones (Fig. 2C), as also predicted by our scRNA data analysis. Taken together, the data indicate that efficient high TM labeling of Aspm-expressing basal cells marks a highly proliferative basal progenitor that substantially contributes to epidermal homeostasis.

We next investigated the contribution of the Aspm-CreER-marked BL populations to injury repair. We already knew that Aspm-CreER can contribute to short-term (1 week) injury repair in mouse back skin (Kang et al., 2020). However, its longer-term activity during the healing process, its behavior in mouse tail skin and its activity relative to known SR populations, such as the K14-CreER-marked SCs (Mascre et al., 2012), were unknown.

Although the K14-CreER driver induced with a high TM dose indiscriminately marks all basal cell types, a low TM dose induces labeling in rare SR long-lived SCs, which were a subset of extremely infrequently dividing SCs (Mascre et al., 2012; Sanchez-Danes et al., 2016). That is, lower TM dosage can target cellular subsets with distinct characteristics, likely due to their higher marker and (hence CreER driver) expression. The K14^low^ ™-CreER-marked SCs contributed robustly to wound healing, whereas an Involucrin-CreER-marked NSR population showed much impaired contribution (Mascre et al., 2012). Interestingly, the Aspm-CreER showed impaired contribution to back skin wound healing relative to the K14^low^ ™-CreER SCs (Fig. S3C-E). This suggested that the Aspm-CreER-marked cells may contain a fraction of NSR progenitors. To study this further, we tested tail skin wound repair after inducing Aspm-CreER and Dlx1-CreER mice at high and low TM doses (Fig. 2D). With high TM dose labeling, both Aspm-CreER^high^ ™ and Dlx1-CreER^high^ ™ progenitors contributed equally to tail skin injury repair (Fig. 2E,F). Interestingly, the Aspm-CreER^low^ ™-marked cells showed impaired contribution to wounding when compared with the Dlx1-CreER^low^ ™-marked cells (Fig. 2E,F). Altogether, the data suggest that there are at least two subpopulations of Aspm-CreER-marked progenitors: one with long-term persistence that contributes robustly to long-term homeostasis and injury repair (i.e. a potential SR SC), and another that shows impaired contribution to injury and may be an NSR progenitor.

### Aspm-CreER^low TM^ marks biphasic NSR progenitors and Dlx1-CreER^low TM^ marks SCs

**Fig. 3.**
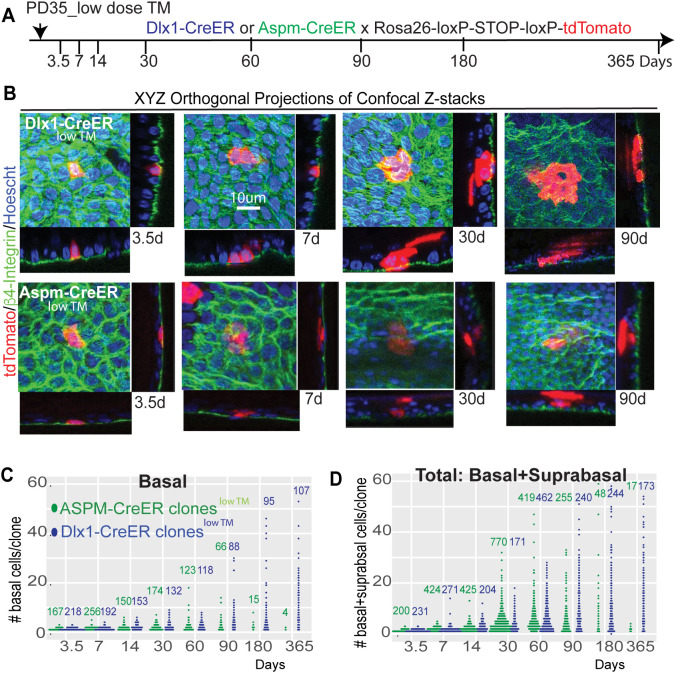
**Clonal genetic lineage tracing for Dlx1-CreER and Aspm-CreER epidermal progenitors.** (A) Schematic of low dose tamoxifen (TM) induction in mice injected once at PD35 and sacrificed at the chase times indicated. (B) *xyz* orthogonal projections through optical *z*-stacks from whole-mount skin collected from the chased mice stained with β4-integrin to mark the basal layer show the basal and suprabasal tdTomato^+^ clones and cells. (C,D) Beeswarm plots of tdTomato^+^ clone and cell counts in images like those shown in B from comparable tail skin areas of Aspm-CreER^low TM^ and Dlx1-CreER^low TM^ lineage traced mice (Table S1). Numbers at the top indicate the total number of clones counted.

To quantitatively study the SR versus NSR long-term behavior of epidermal cells (Blanpain and Fuchs, 2009), we sparsely labeled single rare BL cells (<1/700 BL in all images) in Aspm-CreER mice and Dlx1-CreER control mice using low TM induction followed by quantitative clonal analysis over a year (Fig. 3). Whole-mount tail skin collected from the two mouse lines at the indicated chase-time points (Fig. 3A) were stained with BL marker β4-integrin, and *xyz*-automated high-resolution confocal microscopy captured BL and sBL clones in their entirety (Fig. 3B). *Z*-stacks of over 2500 (Aspm-CreER^low^ ™) or 1725 (Dlx1-CreER^low^ ™) clones in total were assayed at the experimental time points (Fig. 3C,D; Table S1). There was no even-cell clone bias (Fig. 3C,D), as reported for K14^low TM^-CreER cells in mouse back skin (Aragona et al., 2020).

**Fig. 4.**
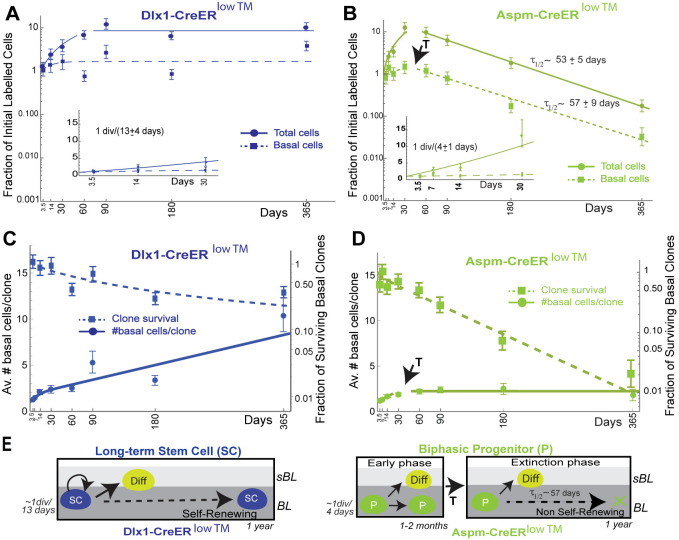
**Dlx1-CreER and Aspm-CreER clonal long-term lineage tracing data.** (A,B) The total (solid) and BL (dashed) relative labeled cell fractions computed from the data in Fig. 2C,D. (C,D) The average numbers of labeled BL cells/clone (solid; left ordinate) and surviving fractions of labeled BL clones (dashed; right ordinate). The arrows in B and D point to the Aspm-CreER^low TM^ transition between early and late phase. (E) Summary of data in A-D. *P*-values testing the similarity of the Dlx1- and Aspm-CreER^low TM^ relative cell fractions, BL clone survival, and average BL clone size were all <10^−10^. Best-fit, standard error, and *P*-value calculations are described in supplementary Materials and Methods, ‘Analysis of Lineage Tracing Data’ and ‘Statistical Methods’.

To examine in depth the behaviors of the two progenitor populations, the clonal data were quantified to calculate: (1) cell fraction of initial labeled cells (‘labeled cell fraction’: the number of tdTomato^+^ cells/initial tail area relative to that at chase-time t=0) in both BL and total (BL+sBL) layers (Fig. 4A,B); (2) average number of labeled basal cells/clone (‘average clone size’; Fig. 4C,D, left ordinate); and (3) fraction of surviving labeled basal clones (‘clone survival’; Fig. 4C,D, right ordinate label) (see supplementary Materials and Methods, ‘Analysis of Lineage Tracing Data’ and ‘Statistical Methods’ for details). Although both Dlx1-CreER^low^ ™- and Aspm-CreER^low^ ™-marked cells and clones were enriched in interscale relative to scale, as we previously reported for high TM dose (Sada et al., 2016; Ghuwalewala et al., 2022) (Fig. S4), there was no significant difference between their clonal dynamics in these two locations (Fig. S5). This indicates that the scale and interscale microenvironments are not dictating overall population behavior, as each of the populations maintains its characteristic behavior in the different locations. Because of this, for better statistical power, we used the combined scale+interscale data for further analysis.

The combined (i.e. scale+interscale) Dlx1-CreER^low^ ™-marked BL cell population behaved similarly to previously described SR epidermal populations that displayed neutral drift (Clayton et al., 2007; Mascre et al., 2012; Sanchez-Danes et al., 2016). Specifically, the fraction of BL labeled cells increased by ∼70% during the first month before reaching a constant level (Fig. 4A), after which time mean BL clone size increased linearly while BL clone survival decreased inversely (Fig. 4C). The labeled total cell fraction increased approximately ninefold during the first 2 months of chase, with the BL cells dividing on average once every 13±4 days (mean±s.e.m.), before it stabilized, presumably because of shedding of tdTomato^+^ cells from the cornified layer (Fig. 4A). Based on this data we conclude that Dlx1-CreER^low^ ™ marks a long-term SR SC population that maintains its BL numbers (i.e. self-renews) and differentiates to sBL cells. This SC population divides around three times slower than the reported average BL cell division rate of approximately once per 5 days (Potten et al., 1982; Sada et al., 2016). The transient small increase in the labeled BL cell fraction suggests that this SC, like the previously reported K14^low^ ™-CreER marked population (Mascre et al., 2012; Sanchez-Danes et al., 2016), generates additional short-lived progenitors whose BL cell fraction increases until steady-state is reached. However, the Dlx1-CreER^low^ ™-marked division rate is at least five times that of K14^low^ ™-CreER-marked cells, which divide only four to six times per year (Mascre et al., 2012; Sanchez-Danes et al., 2016), indicating that the two marked populations are not identical.

In striking contrast to Dlx1-CreER^low^ ™, the Aspm-CreER^low^ ™ population did not self-renew in the long-term, demonstrating that it is an NSR progenitor (Fig. 4B,D) as suggested by the wound healing data (Fig. 2D-F). Furthermore, the BL labeled cell fraction increased slightly during an ‘early phase’ that lasted between 30 and 60 days, when it transitioned into a second phase where it decreased around 25 times by 1 year, undergoing ‘extinction’, with a constant exponential half-life of 57±9 days (Fig. 4B). These data indicate that the Aspm-CreER^low^ ™-marked BL cells not only represent an NSR progenitor, but one that undergoes a biphasic behavior during homeostasis; together these are predicted characteristics of a TA cell. This biphasic behavior of a TA cell differs from the monophasic, ‘constant’ behavior of the previously described NSR Involucrin-CreER-marked progenitor (Sanchez-Danes et al., 2016) whose BL cell fraction is extinguished at a constant exponential rate from the start of the chase (Fig. S6A,C).

The Aspm-CreER^low^ ™-marked total cell fraction increased linearly by approximately ten times during the first 30 days, with the BL cells dividing on average once every 4±1 days. This was around three times faster than the Dlx1-CreER^low^ ™-marked BL cells and slightly faster than the average BL division rate, in line with the expected frequent divisions of a TA cell. As with its BL cell fraction, by 60 days the Aspm-CreER^low^ ™ total (basal+suprabasal) cell fraction was decreasing, with a half-life of 53±5 days (Fig. 4B), indicating that TD exceeds SR in the extinction phase.

The apparent change observed in the fraction of cells suggested a possible biphasic behavior with a transition between 30-60 days. This was confirmed by the behavior of the Aspm-CreER^low^ ™-marked BL average clone size: after increasing for 30-60 days in early phase, it rapidly stabilized at ∼2 cells/clone in extinction phase, during which period BL labeled clone survival decreased exponentially to near extinction (Fig. 4D). Like the BL cell fraction, this contrasts with the behavior of the monophasic NSR Involucrin-CreER-marked population (Sanchez-Danes et al., 2016), for which the average clone size undergoes smooth exponential relaxation toward its asymptotic value (Fig. S6B,C). We conclude that Aspm-CreER^low^ ™ marks a novel biphasic NSR population that initially divides rapidly in an early growth phase before a subsequent transition to an extinction phase (Fig. 4E). This biphasic behavior is characteristic of a TA cell and contrasts with that of Dlx1-CreER^low^ ™-marked SR progenitors (Fig. 4E) and that of all previously reported SR and NSR BL progenitors (Clayton et al., 2007; Mascre et al., 2012; Sanchez-Danes et al., 2016; Piedrafita et al., 2020; Cockburn et al., 2022).

### Lineage tracing may underestimate the BL amplification of a TA progenitor

To maintain homeostasis, TA cell loss must be counterbalanced by constant replenishment with new (i.e. ‘nascent’) TA cells derived from a SC or another BL precursor (Fig. S7). A nascent TA cell and its ‘pre-transition’ BL descendants will initially amplify their BL numbers and have a ‘positive fate imbalance’, where the rate of SR dominates that of TD (Fig. S7). Eventually, as in the well-known ‘crisis’ of primary cell cultures that occurs when their proliferative limits are reached (Hayflick and Moorhead, 1961; Potten, 1974; Potten et al., 1982), the TA cells will exhaust their potential, enter a ‘post-transition’ state having a negative fate imbalance favoring TD over SR, and begin their decline towards extinction (Fig. S7). Therefore, a homeostatic TA population in the BL must be a mixture of clones founded by nascent cells as they were introduced in the lineage from a precursor cell at varying times in the past. This mixture comprises nascent, pre-transition and post-transition cells with varying fate imbalances that depend on their ‘clonal age’ – the time spent since the generation of their clone's founding nascent cell from a precursor. Initial marking by a CreER genetic driver labels this mixture of cells with different clonal ages in proportion to their representation in the homeostatic population, but a potential CreER labeling bias towards cells of a specific clonal age could also influence the labeling proportions. Therefore, unless only nascent TA cells are initially labeled, the BL amplification observed in lineage tracing during early phase will always appear to be diminished by contributions from initial labeling of post-transition cells that exist in the mixture (supplementary Materials and Methods, ‘Inferring the Biological Properties of Evolving NSR Progenitors from Lineage Tracing Data’). Therefore, to extract the true biological behavior of the nascent TA cell in homeostasis from the lineage tracing data and to rigorously test the TA progenitor model requires deconvolution of the cells of potentially different clonal ages (Fig. 4B,D).

Previously, the CBDM (Klein and Simons, 2011; Blanpain and Simons, 2013) was used to mathematically analyze clonal long-term lineage tracing data of SR progenitors with constant, balanced SR and TD fates (Clayton et al., 2007; Mascre et al., 2012; Sanchez-Danes et al., 2016). However, because of the complications above, this type of modeling is not sufficient for the clonal analysis of an NSR TA population (Fig. S7). To address this, we developed the ‘generalized birth-death model’ (GBDM) described in supplementary Materials and Methods, ‘Inferring the Biological Properties of Evolving NSR Progenitors from Lineage Tracing Data’.

### Aspm-CreER^low TM^-labeled clones undergo neutral competition without neutral drift

**Fig. 5.**
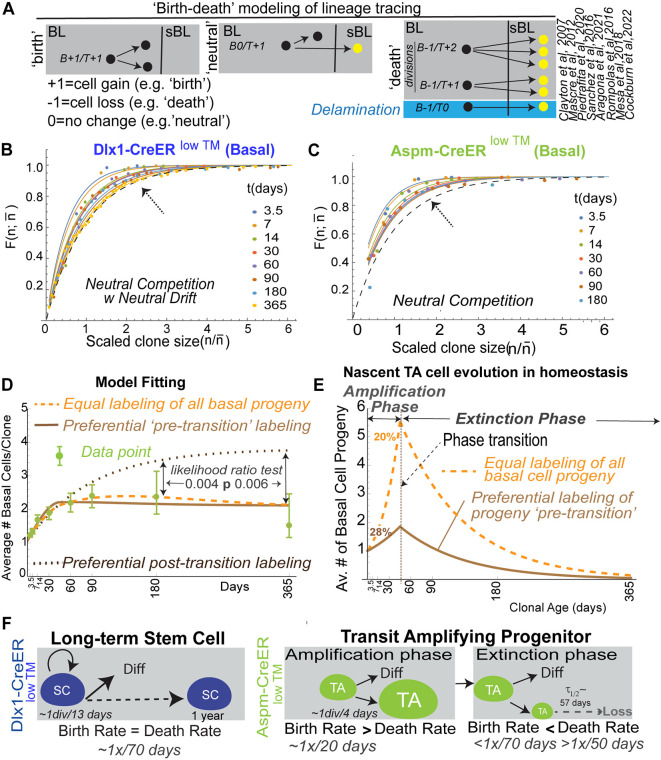
**Generalized birth-death analysis of Aspm-CreER^low TM^-labeled cells and biological BL clone dynamics in homeostasis.** (A) Simplified schematic of biological processes considered in ‘birth-death’ modeling of clonal lineage tracing. The effect on each process on the observed data on the timescale of lineage tracing is shown; e.g., B0/T+1 means that BL clone size and cell fraction is unchanged while total cell fraction increases (for an expanded version see Fig. S8). (B,C) Cumulative labeled clone size distributions at the indicated chase times (see also Fig. S9). 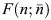 is the fraction of clones with size ≤*n*, where 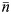. is the average clone size at the specified chase-time. Dots indicate experimental values. Solid lines are the neutral competition predictions of Eq. 2. The dashed black lines (identified by arrows) mark the asymptotic ‘neutral drift’ limit of Eq. 1, which is reached by the large Dlx1-CreER^low TM^-marked clones but not by the Aspm-CreER^low TM^-marked clones. (D) Basal labeled cell fraction (Fig. 4B) and basal labeled clone size data (Fig. 4D) were used together to compute the best-fits for Aspm-CreER^low TM^ progenitor birth and death rates, with a transition time at 45 days (supplementary Materials and Methods, ‘Analysis of Lineage Tracing Data’). The predictions for three models encompassing potential CreER labeling biases are compared with the data (see Fig. S10 for model-predicted labeled BL cell fractions). Likelihood ratio tests reject the ‘post-transition’ cell labeling model, which is equivalent to a monophasic model, with (1−*P*-value) confidence levels of >0.994. (E) Predicted average number of BL Aspm-CreER^low TM^ cells descended from a single nascent cell as a function of clone age (i.e. time nascent cell introduction of the clone's nascent cell founder) for the models indicated. The amount of pre-transition progenitor amplification and the percentage of cells in the amplification phase during homeostasis are shown for the two labeling models that are consistent with the data. (F) Summary of stem and progenitor cell behavior during homeostasis of adult epidermis.

Previous clonal lineage tracing of SR populations has shown that labeled BL clone loss over time is compensated by labeled clone size increase, which the CBDM explains by ‘neutral drift’ (Clayton et al., 2007; Mascre et al., 2012; Lim et al., 2013; Piedrafita et al., 2020). Labeled BL clones will display neutral drift when progenitors have similar potential and their SR and TD fate choices are: (1) stochastic and independent (i.e. progenitors undergo ‘neutral competition’) and (2) always ‘balanced’ (i.e. occur with equal and constant rates) (Fig. S7). It is important to recognize that the relationship between biological events and the events measured in clonal lineage tracing can be complicated: Biological events that co-occur sequentially within a few days (i.e. as observed in short-term live microscopy; Rompolas et al., 2016; Mesa et al., 2018) are recorded as single events in long-term lineage tracing due to infrequent time sampling (Fig. 5A; Fig. S8). Moreover, biologically symmetric divisions, in which both daughter cells remain in the BL, appear as ‘births’ that increase labeled clone size, whereas symmetric divisions, in which both daughter cells are exported into sBL, appear as ‘deaths’ that decrease BL clone size. Asymmetric divisions and symmetric divisions correlated with delamination of one daughter cell do not affect BL clone size; i.e., they are ‘neutral’ (Fig. 5A; Fig. S8).

The CBDM has been previously tested by comparing its prediction of long chase-time SR BL clone sizes with the lineage tracing data. It predicts that labeled SR BL clones with similar growth properties having size *n*′≤*n* with average clone size 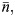 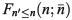
will asymptotically approach
(1)

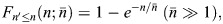
when 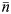 becomes large (Klein and Simons, 2011; Blanpain and Simons, 2013). This has been validated for multiple SR populations (Clayton et al., 2007; Mascre et al., 2012; Lim et al., 2013; Piedrafita et al., 2020). After the initial transient increase, the Dlx1-CreER^low^ ™-marked BL clones satisfy this prediction (Fig. 5B; Fig. S9A), so we conclude that they undergo neutral drift.

Even though the Aspm-CreER^low^ ™-marked BL progenitors are not maintained, implying that they eventually must make imbalanced fate choices (Fig. 4B), and their clones do not undergo neutral drift (Fig. 5D), their fate choices may still be stochastic and independent – i.e. their BL clones may undergo neutral competition (Fig. S9). The GBDM we developed to analyze this situation predicts that the cumulative size distribution of BL clones with similar growth properties that undergo neutral competition, with or without neutral drift, will be
(2)

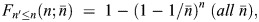
at all chase-times, even if the average clone size 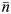 does not get large (supplementary Materials and Methods, ‘Inferring the Biological Properties of Evolving NSR Progenitors from Lineage Tracing Data’) – Eqn 1 approximates Eqn 2 if the average clone size becomes large. Eqn. 2 is a good approximation even if the birth and death rates change with TA clonal age and provides a robust test of neutral competition, with or without neutral drift. The experimental Dlx1-CreER^low^ ™- and Aspm-CreER^low^ ™-marked clone size distributions at all chase times agree with Eqn. 2 (Fig. 4C,D; Fig. S9). Therefore, applying the GBDM to our data confirms that the Dlx1-CreER^low^ ™ clones undergo neutral drift and, importantly, shows that the Aspm-CreER^low^ ™ clones undergo neutral competition without neutral drift (Figs 5B,C; Fig. S9).

### Modeling Aspm-CreER^low TM^ TA progenitor behavior in homeostasis

Using the GBDM to deconvolve the combined Aspm-CreER^low^ ™-marked BL fraction and average clone size data (Fig. 4B,D), we can infer and model biological properties of the nascent Aspm-CreER^low^ ™-marked progenitors and their descendants as they develop in homeostasis. Specifically, we calculate that the progenitor birth rate decreases at least twofold at the transition contributing, at least in part, to the change from amplification to extinction, and that the birth and death rates are constant in extinction phase. However, calculating additional properties of TA progenitor behavior depends on the potential variation of Aspm-CreER^low^ ™ driver labeling efficiency with clonal age. Therefore, we consider three cases: (1) all TA cells are labeled equally; (2) only pre-transition (i.e. in amplification phase) TA cells are labeled; and (3) only post-transition (i.e. in extinction phase) TA cells are labeled (Fig. 5D). The lineage tracing data for the third model will be the same as that of a monophasic NSR progenitor model because the labeled post-transition cells never go through further transition. The lineage tracing data indicates that the transition occurs (probably gradually) between 30 and 60 days (Fig. 4B,D) but, to avoid overparameterization, we model an abrupt transition at 45 days.

The best-fits of the three models to the experimental average BL labeled clone size data and cell fraction data are shown in Fig. 5D and Fig. S10, respectively (see supplementary Materials and Methods, ‘Analysis of Lineage Tracing Data’ for details). Both the equal- and pre-transition cell-labeling models provide good fits to both sets of data, but the post-transition cell-labeling/monophasic model does not. In the latter case, the Aspm-CreER^low^ ™-marked average BL clone size relaxes exponentially to an asymptotic value, as was observed for Involucrin-CreER-marked BL clones (Fig. S6B). Taking all the data into account, the likelihood-ratio test rejects the post-transition labeling model relative to either of the biphasic models with confidence levels ≥0.994, statistically confirming that Aspm-CreER^low^ ™ is a biphasic NSR progenitor, i.e. a TA cell. Importantly, both biphasic models predict that pre-transition cells favor SR over TD, implying that the average number of nascent TA cell descendants in the BL increases in amplification phase ∼2- to 5.6-fold before transition into extinction phase (Fig. 5E). This increase is diminished in lineage tracing observations because >70% labeled cells are already in extinction phase at the start of the experiment in both models. We conclude that, irrespective of any potential bias of Aspm-CreER^low^ ™ driver to label specific clonal ages, our modeling of the data indicates that the nascent TA cell indeed amplifies the BL cell number before transition and the majority of TA cells in the homeostatic population are in extinction phase.

## DISCUSSION

This study applied single cell genomics, genetic lineage tracing and mathematical clonal analysis to the mouse epidermis and provided evidence in support of a stochastic version of a classical SC-TA tissue homeostasis theory (Fig. S7) (Potten, 1974; Potten et al., 1982; Potten and Loeffler, 1990). This theory implies that, in addition to slow-cycling long-term SR progenitors (e.g. SCs), the epidermis is maintained by frequently dividing NSR progenitors with biphasic TA behavior (Fig. 5F; Fig. S7). Previously, H2B-GFP pulse-chase experiments showed that Dlx1 and Aspm mRNAs are upregulated in slow-cycling LRCs or in rapidly dividing non-LRCs BL cellular subsets, respectively (Tumbar et al., 2004; Sada et al., 2016). Here, clonal analysis identified Dlx1-CreER^low^ ™-marked progenitors as long-term SR SCs that indeed divide around three times less frequently than Aspm-CreER^low^ ™-marked NSR progenitors. This agreed with our scRNA-seq data, where over 90% of Aspm^+^ (but not Dlx1^+^) cells were actively in the cell cycle. The Dlx1-CreER^low^ ™-marked SCs were more active (∼1 division/13 days) than previously reported K14^low^ ™-CreER-marked SCs (4-6 divisions/year) (Mascre et al., 2012; Sanchez-Danes et al., 2016).

Most importantly, our study uncovered an Aspm-CreER^low^ ™-marked BL subset as the long-predicted, first identified NSR biphasic TA epidermal progenitor *in vivo*. To maintain homeostasis, NSRs must be maintained by the continuous introduction of BL nascent cells differentiated from a SR or other progenitor precursor. Unlike previously identified NSR progenitors, the descendants of the Aspm-CreER^low^ ™ nascent cell population initially undergo an amplification phase when their SR rates exceed their TD rates, increasing BL cell numbers. Later, the descendants undergo a timed transition when TD exceeds SR, resulting in their extinction from tissue (Fig. 5F; Fig. S7). This novel biphasic NSR TA progenitor differs from two previously reported epidermal BL NSR progenitors marked by Involucrin-CreER (Mascre et al., 2012; Sanchez-Danes et al., 2016) and K10-CreER (Cockburn et al., 2022) that are monophasic, have constant imbalance towards TD and display different cellular kinetics and lifespans.

We extended CBDM (Klein and Simons, 2011; Blanpain and Simons, 2013), which has been widely applied to clonal analysis of SR cells to GBDM, which is applicable to NSR and TA cells with biphasic behavior, and validated it by testing its labeled clone size distribution predictions against the Dlx1-CreER^low^ ™ and Aspm-CreER^low^ ™ data. This mathematical apparatus will serve as basis for broader analysis of NSR populations with variable behavior over time in other progenitor and tissue systems. Using the GBDM we concluded that over 70% of the TA cells were in extinction phase in homeostasis, and that nascent Aspm-CreER^low^ ™ cells are amplified by several fold in the BL while in their amplification phase. Furthermore, we determined that, to maintain homeostasis, this TA population only requires the rare introduction of a nascent cell by differentiation from a BL precursor at a rate of <0.5%/day. The rarity of this event is a consequence of the early BL amplification and slow extinction rate of this TA progenitor. This, together with the relative long time to transition of 30-60 days, explains why this unique TA behavior has not been observed in short-term (1-2 weeks) live imaging or in long-term lineage tracing with ubiquitous markers (Clayton et al., 2007; Lim et al., 2013; Rompolas et al., 2016; Mesa et al., 2018).

Unlike Involucrin-CreER-marked NSR progenitors (Mascre et al., 2012; Sanchez-Danes et al., 2016), the Aspm-CreER^low^ ™-marked TA progenitors contribute to wound healing, albeit still less robustly than the SCs. Interestingly, the behaviors of both our SR and NSR progenitors in lineage tracing were not influenced by their specific localization in scales versus interscale. Therefore, the differences between their population behaviors are driven either by cell-intrinsic mechanisms or specific micro-niches for each population that must be present in both scales and interscales.

The TA progenitor behavior identified here deviates from the classical SC→TA→TD hierarchical model, which presumes that SR and TD events are sequential and rigidly linked, i.e., not stochastic and independent (Potten, 1974; Potten et al., 1982; Potten and Loeffler, 1990). Our Dlx1-CreER^low^ ™ lineage tracing data agree with the modern neutral drift theory for SR progenitors (Clayton et al., 2007; Klein and Simons, 2011; Blanpain and Simons, 2013; Piedrafita et al., 2020), as these SCs make balanced stochastic and independent fate choices. Furthermore, our GBDM analysis of the Aspm-CreER^low^ ™-marked TA data shows that even this biphasic NSR population makes fate choices that are stochastic and independent (though imbalanced in opposing ways in their amplification and extinction phases). These flexible stochastic fate choices deviate from the early model (Potten, 1974; Potten et al., 1982; Potten and Loeffler, 1990) and from a related epithelial hair follicle lineage, where SC, TA and TD cell fates are linked in a strict sequence (Zhang et al., 2009). Finally, the short TA progenitor life span predicted by early models (∼2 weeks) (Potten, 1974; Potten et al., 1982; Potten and Loeffler, 1990) fits more the previously described K10-CreER-marked NSR population (Cockburn et al., 2022) than the Aspm-CreER^low^ ™ TA progenitor, which has a much longer extinction time (∼6 months).

Previous modeling of the epidermis BL as a single progenitor that undergoes neutral drift was based on the use of ubiquitous genetic drivers that reported the combined behavior of indiscriminately marked BL mixtures (Clayton et al., 2007; Lim et al., 2013; Rompolas et al., 2016; Mesa et al., 2018; Piedrafita et al., 2020). In contrast, accumulating evidence indicates that the epidermal BL is heterogeneous: several molecularly distinct BL cell states have been uncovered by scRNA-seq of mouse and human skin (Joost et al., 2016; Dekoninck et al., 2020; Haensel et al., 2020; Lin et al., 2020; Wang et al., 2020; Ghuwalewala et al., 2022) and by bulk RNA-seq of sorted subsets (Mascre et al., 2012; Sada et al., 2016; Sanchez-Danes et al., 2016; Ghuwalewala et al., 2022). Moreover, genetic clonal analysis and live imaging of the BL shows that it contains multiple progenitors including SR SCs, monophasic NSR progenitors, and now biphasic TA progenitors (Mascre et al., 2012; Sada et al., 2016; Sanchez-Danes et al., 2016; Cockburn et al., 2022; Ghuwalewala et al., 2022). An open question is whether these SR and NSR progenitors are stable or reversible cell states of a single BL progenitor. Clearly, these states do not rapidly fluctuate, or else stable clonal evolution profiles could not emerge in 1 year lineage tracing experiments. Another open question is that of the hierarchy among the various known (and yet to be discovered) SR and NSR BL cell states in homeostasis. This hierarchy cannot be unambiguously determined with traditional lineage-tracing approaches employed so far in the skin. Interestingly, although low TM dose induction (likely capturing cells highly expressing Aspm) labels an NSR TA population (this study), high TM dose induction captures cells with long-term SR ability (e.g. SCs) in both tail skin (this study) and back skin (Kang et al., 2020). As Aspm expression is upregulated in a large fraction of G2/M cells (Capecchi and Pozner, 2015), it is tempting to speculate that TA progenitors are high Aspm-expressing BL cells that are produced at rare divisions from BL SCs that are low Aspm expressing. This possibility and the inter-connection of other SR and NSR BL progenitors could be addressed in the future, using more sophisticated *in vivo* lineage mapping approaches combined with clonal bar coding (Bowling et al., 2020; Pei et al., 2020).

The discovery of an epidermal TA progenitor that transitions from amplification into extinction phase *in vivo* during normal homeostasis is arguably the most fascinating aspect of our work. The fate transition mechanism remains to be explored in the future, but we speculate that it involves dilution of a factor(s) initially produced either in the nascent cell or in its micro-niche that is diluted over time due to cell division or simple RNA/protein degradation until a critically low threshold is reached at ∼30-60 days, prompting an abrupt transition into the extinction phase. As envisioned by Potten et al. many decades ago (Potten, 1974; Potten et al., 1982; Potten and Loeffler, 1990), elucidating the mechanism of TA cell fate transition during homeostasis could bring us one step closer to understanding aging and cancer.

## MATERIALS AND METHODS

### Mouse care

All mouse work was executed according to Cornell University Institutional Animal Care and Use Committee guidelines (protocol number 2007-0125). Both male and female mice have been used in the study without discrimination.

### Single cell RNA-seq data analysis

The scRNA-seq library of high-quality BL (Sca1^+^/α6-integrin^+^) cells described in Ghuwalewala et al. (2022), with ∼50,000 reads/cell of ∼15,000 tail skin cells merged from two PD52 C57BL6 mice was analyzed. PCA and UMAP dimensionality reductions, clustering and feature plots (Fig. 1A-G) were performed using the Seurat v3 (Butler et al., 2018) R package (version 3.0.1) as previously described (Ghuwalewala et al., 2022). Seurat was used to identify cells with Aspm or Dlx1 expression values >0, and then to compute the fractions of cells in these groups that expressed Ki67>0, Involucrin>0, or K10>3.5 (Fig. 1B,C). In Fig. 1A the K10>3.5 cutoff was chosen to select the K10^high^ BL cells found in the K10^+^ cluster, which was shown to be differentiating by Cockburn et al. (2022).

### Cell cycle regression analysis

Percentages of cells in G1, S and G2/M in different basal subsets (Fig. 1E) were obtained by cell-cycle analysis using the Seurat package (Butler et al., 2018). Trajectory analysis was performed using Monocle 3 (Trapnell et al., 2014) after regressing the S and G2/M genes using the ScaleData function in Seurat (Fig. S2). Feature plots for the relevant markers were plotted for the given UMAP coordinates obtained after cell cycle regression.

### Pseudo-bulk differential gene expression analysis

To determine the genes that were differentially overexpressed by the Aspm^+^ cells, we selected the reads from all the 14,883 scRNA-seq tail BL cells having Aspm expression >0 and identified the DEGs in Aspm^+^ versus Aspm^−^ cells using the Seurat FindMarkers function (Fig. 1F).

We differentiated the cycling versus non-cycling Aspm populations by subdividing the Aspm^+^ population into Ki67^+^ and Ki67^−^ cells, and then calculated DEGs between them (Fig. S1F,G). Genes having 1.5- to 2-fold higher expression than the comparable group and an adjusted *P*-value <0.05 were selected as significant (Table S2).

To understand the biological significance and functional categories of the DEGs, we performed Gene Ontology (GO) analysis using EnrichR (Edward et al., 2013). Significant pathways with adjusted *P*-value <0.05 were represented as bubble plots using the R package.

To compare the Aspm^+^/Ki67^+^ and Dlx1^+^/Ki67^+^, we first selected all the Ki67^+^ cells from the total BL and then calculated DEGs between the Aspm^+^/Ki67^+^ and total Ki67^+^. Similarly, DEGs were calculated between Dlx1^+^/Ki67^+^ and total Ki67^+^ and these combined DEGs were used for GO predictions (Fig. 1G). The DEGs for only the Aspm-expressing cells within the chosen clusters were obtained after cell cycle regression (Fig. S2).

### TM injections for lineage tracing and FACS in homeostasis

For lineage tracing, Dlx1-CreER (C57BL6) (Taniguchi et al., 2011) (The Jackson Laboratory, 014551) or Aspm-CreER (Madisen et al., 2010; Marinaro et al., 2011; Kang et al., 2020) mice were crossed with Rosa-tdTomato reporter mice (Madisen et al., 2010) (The Jackson Laboratory, 007905). The K14CreER transgenic mice were provided by Dr Elaine Fuchs (Rockefeller University, New York, USA) and were genotyped as recommended by the manufacturer's primer and protocol. For efficient ‘high-dose’ labeling, Aspm-CreER mice were injected with 100 μg/g body weight TM (Sigma-Aldrich) per day beginning at postnatal day (PD) 49 for 5 consecutive days (Fig. 2) or beginning at PD34 for 2 consecutive days (Fig. S3). Dlx1-CreER mice were always injected five times to obtain efficient ‘high dose’ labeling. Non-TM oil injected CreER^+^/Rosa-tdTomato^+^ mice were used to test CreER leakiness. For clonal lineage tracing, we used ‘low-dose’ TM injections to ensure good spatial separation of the labeled cells: a single injection of TM of 100 μg/g body weight for Dlx1-CreER (one injection of 100 μg/g is ‘low dose’ for Dlx1-CreER) and 10 μg/g body weight for Aspm-CreER mice at PD34. Mice were euthanized at the times indicated after the last injection.

### TM injection for lineage tracing in wound healing

For wound healing experiments, we used the same ‘high TM’ dose and ‘low TM’ dose as in homeostasis. Specifically, for high dose we injected the Aspm-CreER transgenic mice at PD34-35 (1×/day for 2 days, 100 μg/g of body weight each day) and the Dlx1-CreER mice at PD30-34 (1×/day for 5 days, 100 μg/g of body weight each day). The low TM dose scheme for tail skin was the same as that for the clonal lineage tracing experiments described above (one injection of 10ug/g body weight for Aspm-CreER and one injection of 100 µg/g body weight for Dlx1-CreER). For back skin, K14-CreER mice were injected with 1 μg/g body weight and Aspm-CreER mice were injected with 100ug/g body weight each day, for 2 consecutive days. After 2 weeks, a 4 mm punch wound on the back or tail skin was made at the second telogen (PD49). Isoflurane was used for mouse anesthesia. Mice were injected with ketoprofen (2 μg/g body weight) and amoxicillin (100μg/g body weight) to prevent wound infection. Betadine was topically applied. The mice were then euthanized at various times – 0 day (no wound), 1 day, 1-, 3-, and 5-weeks – and the top view of the wound bed was imaged as described in the following sections. The tdTomato intensity per wound bed area was normalized according to initial labeling intensity before wounding and quantified using ImageJ.

### Preparation, staining, imaging and quantification of tail epidermal whole mounts

#### Whole mount preparation

To prepare whole-mount tail epidermis for immunofluorescence staining, we dissected the entire tail skin, cut it into smaller pieces (5 mm×5 mm) and incubated them in EDTA (20 mM)/PBS on a shaker at 37°C for 2 h. The epidermis was then separated from the dermis as an intact sheet and fixed in 4% paraformaldehyde (PFA) overnight at 4°C. The intact epidermal pieces of tail skin were washed, incubated in blocking buffer (1% bovine serum albumin, 2.5% donkey serum, 2.5% goat serum, 0.8% Triton in PBS) for 3 h at room temperature and incubated with primary antibodies/blocking buffer overnight at room temperature. Samples were washed four times in PBS with 0.2% Tween for 1 h at room temperature, and were incubated overnight with secondary antibodies at 4°C. After washing, samples were counterstained with Hoechst or DAPI for 1 h and mounted.

#### Antibody dilutions

Rabbit anti-K14 (1:100, BioLegend, 905301) or mouse anti-K10 (1:100, BioLegend, 904301) and rat anti-β4-integrin (1:200, BD Biosciences) or rabbit anti-Ki67 (1:100, Abcam, ab15580) were used. All secondary antibodies (FITC, 712-095-153; Cy5, 712-175-153; or Alexa-594, 712-585-153; Jackson ImmunoResearch) were used at a 1:500 dilution. For mouse primary antibodies, the MOM kit (Vector Laboratories) was used for blocking.

#### Imaging and quantification

Preparations were analyzed using confocal microscopy (Zeiss LSM710 or Zeiss LSM880) with Zen 2012 software using *z*-stack optical sectioning. All confocal data shown in figures are projected *z*-stack images viewed from the basal surface. Counting of cells and clones was performed manually in 3D-stacks of images of tail whole mounts obtained from lineage traced Dlx1-CreER or Aspm-CreER×Rosa-tdTomato mice induced at PD34 and stained for β4-integrin. Clones were analyzed for the number of labeled cells in basal or suprabasal layers at 3.5 days, 1 week, 2 weeks, 1 month, 2 months, 3 months, 6 months and 1 year post TM induction (Table S1). tdTomato^+^ clones in the tail epidermis were counted on *z*-stack confocal images (see data summary in Table S1). Orthogonal views were used to display images in three dimensions to visualize the tdTomato^+^ cells and quantify the number of basal and total cells per clone. Cells were considered as basal when their basal side was positive for β4-integrin. Each image was a stitch of 30 tiles (e.g. *xy* fields of view with their corresponding *z*-stacks). Clones were assigned in each tile and the basal and suprabasal cells were counted using Zeiss Zenblue 2.5 software. Quantifications were independently performed at the various time points for each genotype. More than 60 clones at each time point were counted, except for Aspm at 180 and 365 days, when clones were extremely rare or absent. Clone size beeswarm plots (Fig. 3D,E and Fig. S4C,D) were generated using R package 3.0.1.

### Immunofluorescence staining and imaging of mouse tail and human skin sections

Mouse back and tail skin and human skin sections were stained with rabbit anti-Aspm antibody (1:1000, Proteintech AB19013) to characterize the Aspm expression patterns. For visualizing tdTomato fluorescence, back and tail skin (with intact dermis) from Dlx1-CreER× or Aspm-CreER×tdTomato mice post TM induction at various chase time points, were prefixed in 4% PFA overnight and passed through a sucrose gradient (15% and 30%) before embedding in Optimal Cutting Temperature (OCT) compound (Tissue Tek, Sakura). Frozen OCT sections (10 μm) from mice or human were fixed with 4% PFA for 10 min at room temperature. After blocking in normal serum, sections were incubated with primary antibodies overnight at 4°C. The following day the sections were washed and incubated for 1 h with secondary antibodies at room temperature. After washing, the sections were counterstained with Hoechst 33342 or DAPI and mounted. Preparations were examined using a widefield fluorescent microscope (Nikon) and digitally imaged using a CCD (charge-coupled device) 12-bit digital camera (Retiga EXi; QImaging) and IP-Lab software (MVI). To analyze the level of Aspm expression in the basal layer cells, the Aspm^+^ cells with high or low levels of fluorescence intensity were quantified using ImageJ software. The scale/interscale regions were defined based on the retention of nuclei in the cornified layer in the scale region and/or K10 (interscale) expression. Ki67^+^ cells within tdTomato-labeled cells (Fig. 2) were counted to determine the fraction of proliferating cells in the Aspm-CreER- and Dlx1-CreER-marked lineages.

### Statistical methods

All experiments were independently performed at least twice with n≥2 mice and representative data are shown. The statistical test used was a two-tailed unpaired Student's *t*-test. See supplementary Materials and Methods, ‘Statistical Methods’ for full details.

## Supplementary Material



10.1242/develop.202389_sup1 Supplementary information

Table S2. Gene lists of Differentially expressed genes (DEGs) from various populations for GO analyses.
