# Supplementary material for: A transit-amplifying progenitor with biphasic behavior contributes to epidermal renewal
Source: Development. 2024 Jun 27;151(12):dev202389. doi: 10.1242/dev.202389 (PMC11234368; doi:10.1242/dev.202389)
Supplement: Supplementary information [file develop-151-202389-s1.pdf]

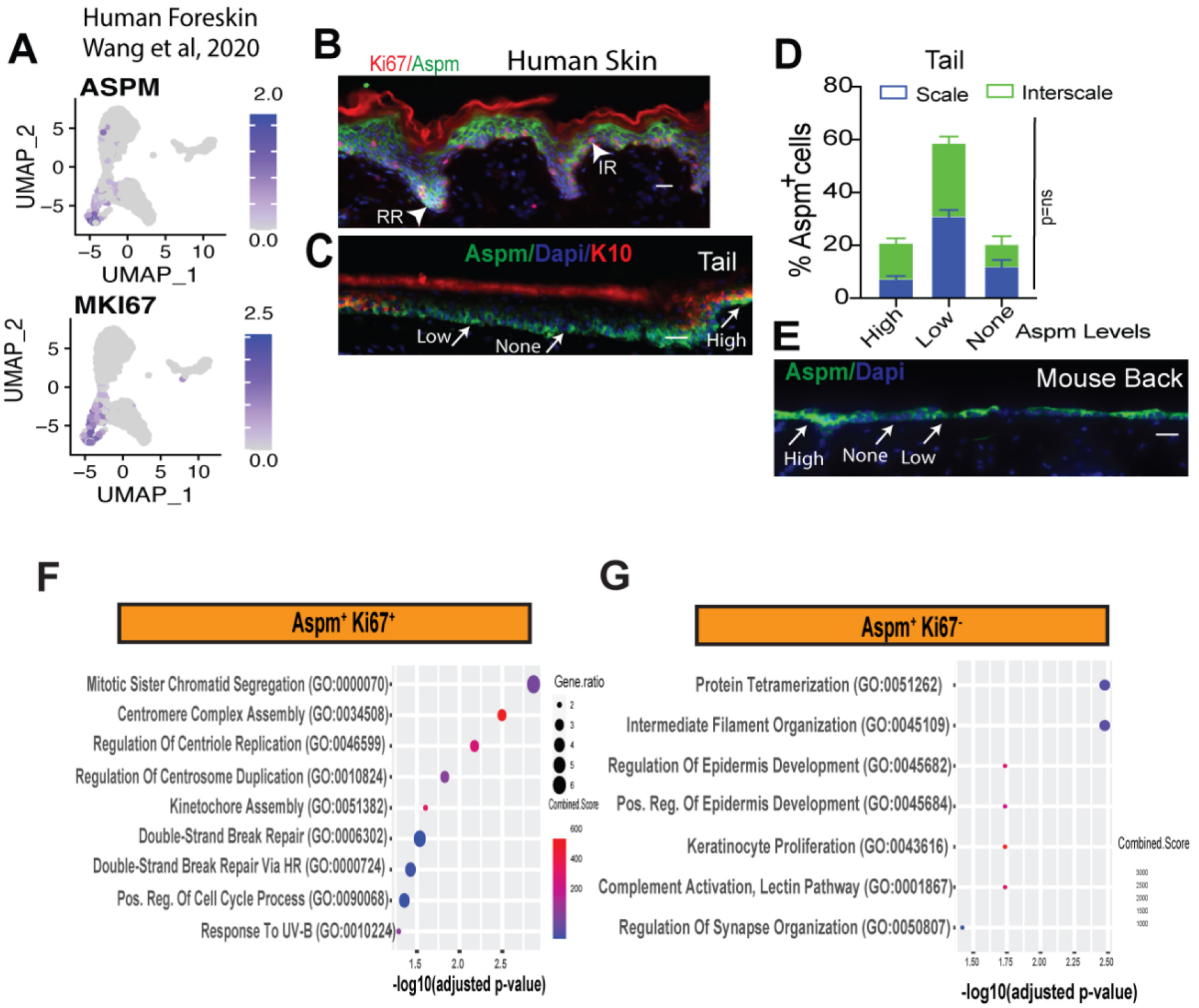

Fig. S1. Aspm expression in human, mouse tail and mouse back skin

(A, B) Aspm mRNA is expressed in human skin in Ki67^{+} proliferative cells, as indicated by scRNA-seq data of epidermal basal cells (A) and immunofluorescence (IF) staining of human skin sections (B) (Wang et al., 2020) (C, D) IF staining of mouse tail epidermis and quantification shows that ~80% of BL cells express some level of Aspm in both scale and interscale. (E) IF of mouse back skin shows Aspm expression in the basal layer. (F, G) Gene set enrichment analysis comparing the Aspm^{+}/Ki67^{+} to the Aspm^{+}/Ki67^{-} BL population. Each plot shows the most significantly enriched GO terms (using GO Biological Process 2023). The x-axis shows the negative $\log_{10}$ of the p-value adjusted for multiple testing (Benjamini-Hochberg correction); Gene.Ratio is proportional to the number of genes associated with the gene set and color represents the combined score calculated by the Enrichr GSEA algorithm (Edward et al., 2013).

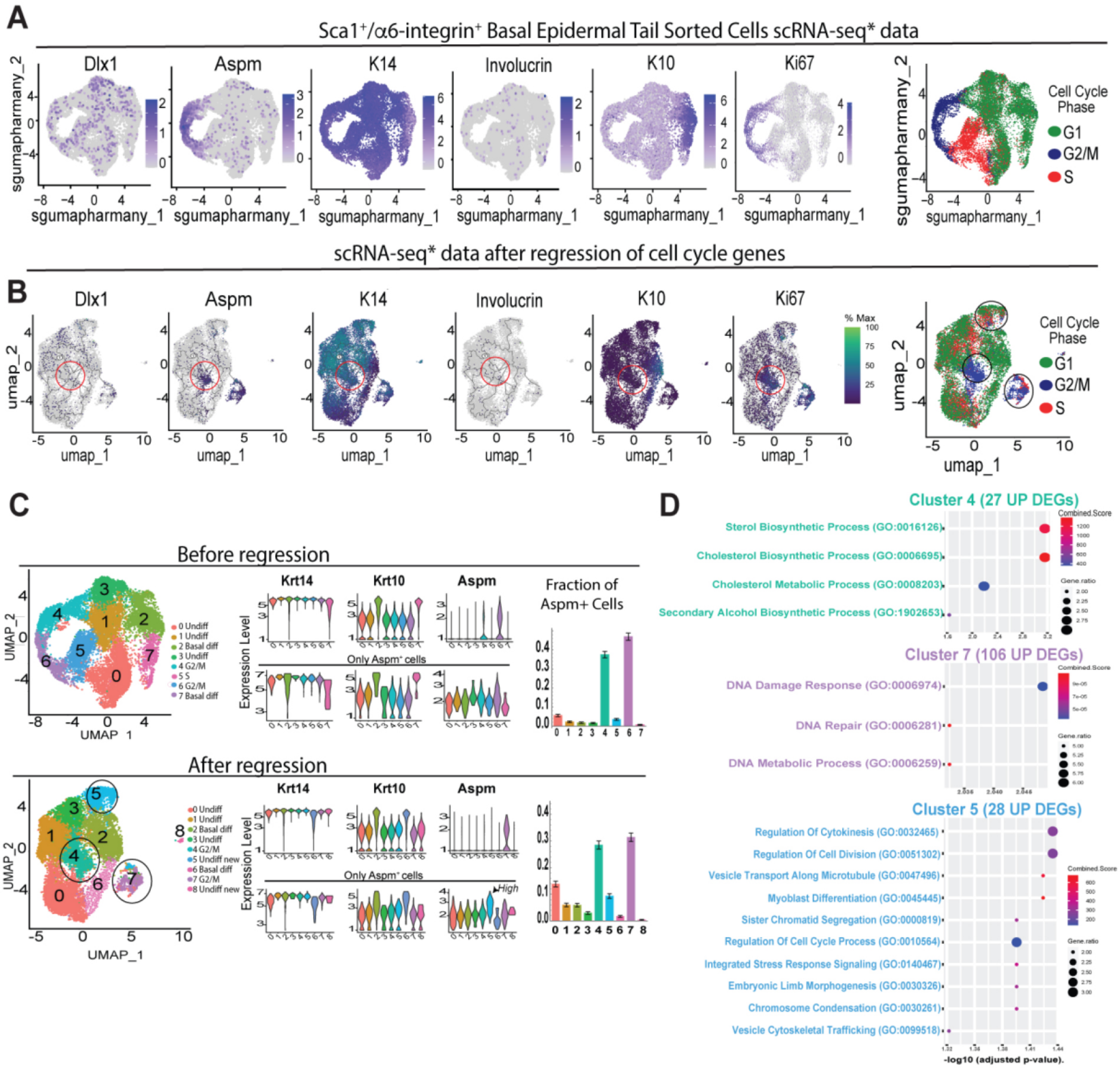

Fig. S2. Regression of cell cycle genes uncovers lineage trajectory and heterogeneity of Aspm⁺ basal cells

(A) scRNAseq feature plots of Sca1⁺/α6-integrin⁺ basal layer (BL) cells sorted from mouse tail skin at PD52 from Fig. 1. (B) Monocle 3 feature plots of the same Sca1⁺/α6-integrin⁺ BL after cell cycle regression show predicted developmental lineage trajectories (black lines); the trajectory's origin is highlighted by the red circles. (It is located in cluster #4 shown identified in the bottom of panel C. The other two circles in the far right panel highlight clusters 5 and 7 of

panel C) Shades of green and blue indicate expression levels as per the legend. (C) UMAP visualization of the Sca1^{+}/α6-integrin^{+} basal layer (BL) datasets before and after regression (left). Cluster cell type annotations are denoted by color and number in the legend. Violin plots of the indicated gene expression levels in the clusters and bar charts of the fractions of Aspm^{+} cells/cluster are shown. Note lower panel graphs showing violin plots of the Aspm+ subset from each cluster. (D) Gene set enrichment analysis for DEGs of Aspm^{+} cells from clusters 4, 5 and 7. Each plot shows the top significantly enriched GO terms (using GO Biological Process 2023). The x-axis shows the negative Log_{10} of the p-values adjusted for multiple testing (Benjamini-Hochberg correction); gene.ratio is proportional to the number of genes associated with the gene set and color represents the combined score calculated by Enrichr GSEA algorithm (Edward et al., 2013).

Development • Supplementary information

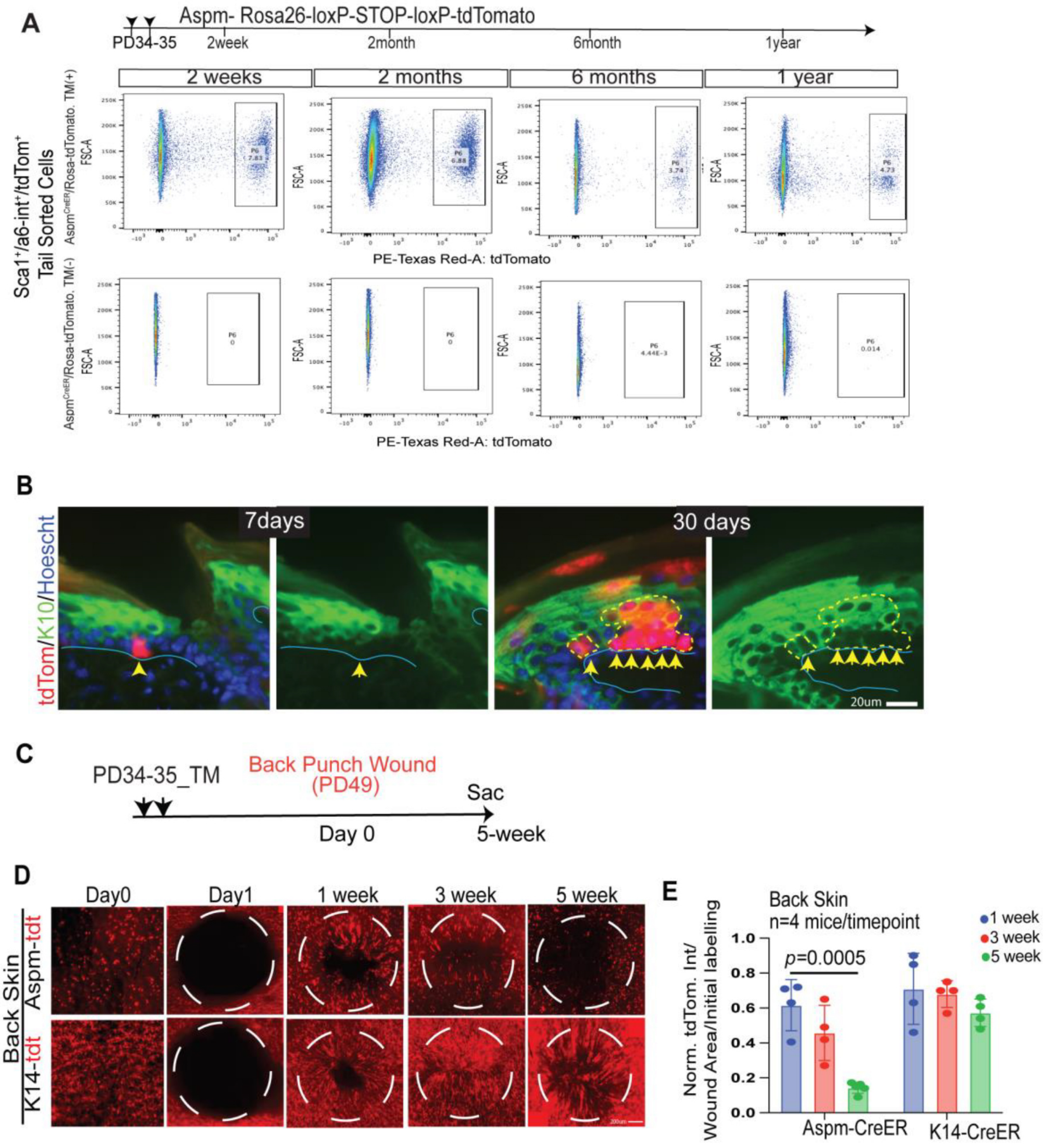

Fig. S3. The Aspm-CreER population marked with high TM dose contributes robustly to skin regeneration.

(A) Schematic of high TM dose lineage tracing in Aspm-CreER mice with two consecutive daily injections and the corresponding flow cytometric profiles of the tdTomato+ cells in the BL (gated

Development • Supplementary information

on Sca1^{+}/α6-integrin^{+}) fraction. P6 denotes the % tdTomato^{+} cells in the BL. Note the persistence of tdTomato^{+} basal cells up to 1-year. Non-injected Aspm-CreER mice (bottom) show no tdTomato^{+} cells at all timepoints analyzed. (B) Examples of IF staining of skin sections from mice in (A) at time points indicated show only rare tdTomato^{+} overlap with differentiating K10^{+} basal layer cells (Cockburn et al., 2022) and strong overlap in the suprabasal layers. (C) Schematic of the punch wound experiment for mouse back skin. (D) Top view images of wounds from back skin of Aspm-CreER and K14-CreER mice showing tdTomato^{+} cells. (E) TdTomato signal intensities within the wounded areas shown in (D) at the indicated times relative to the intensities at day 0. The p-value (n=4 mice, unpaired Student's t-test) compares the 1 and 5-week intensities. Scale bar=200 μm.

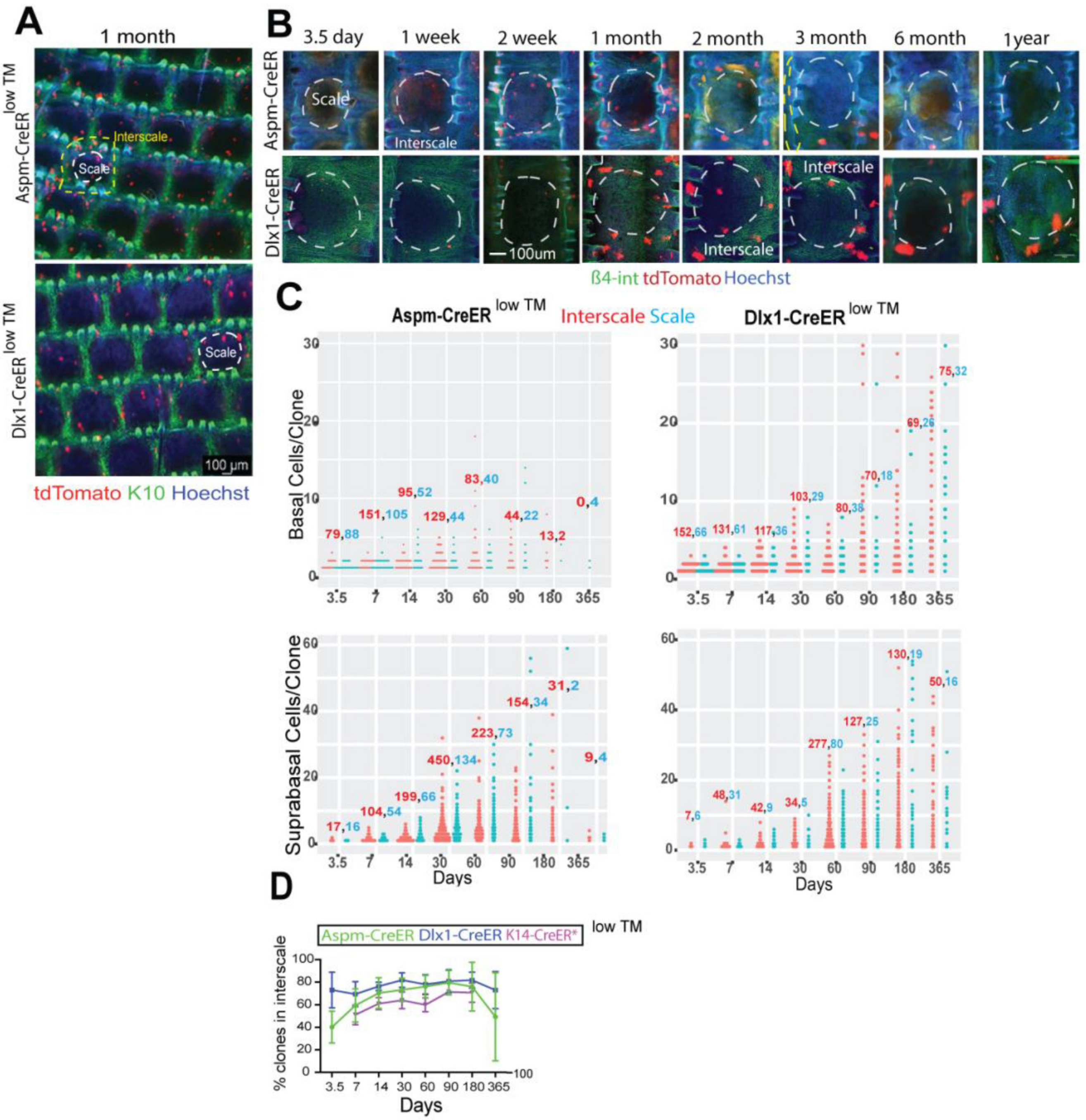

Fig. S4. Lineage tracing of clones in scale and interscale domains

Aspm-CreER and Dlx1-CreER mice were induced with low TM injection at PD35 as in Fig. 3A (A) Whole mount tail skin images from the induced mice stained with K10 antibody compare the clone distributions in scales vs interscales at 1-month post TM induction. (B) Optical Z-projections through confocal Z-stack images like those shown in Fig. 3, but at lower magnification, display

Development • Supplementary information

the clone localization in scale and interscale. (C) Beeswarm plots of $tdTomato^{+}$ clone and cell counts (Fig. 3C,D) separated according to location in scale and interscale. (D) Percent total (basal+suprabasal) clones in interscales for Aspm-$CreER^{low\ TM}$ and Dlx1-$CreER^{low\ TM}$ mice compared with K14-$CreER^{low\ TM}$ data extracted from (Sanchez-Danes et al., 2016) mice over the 1-year chase.

Development • Supplementary information

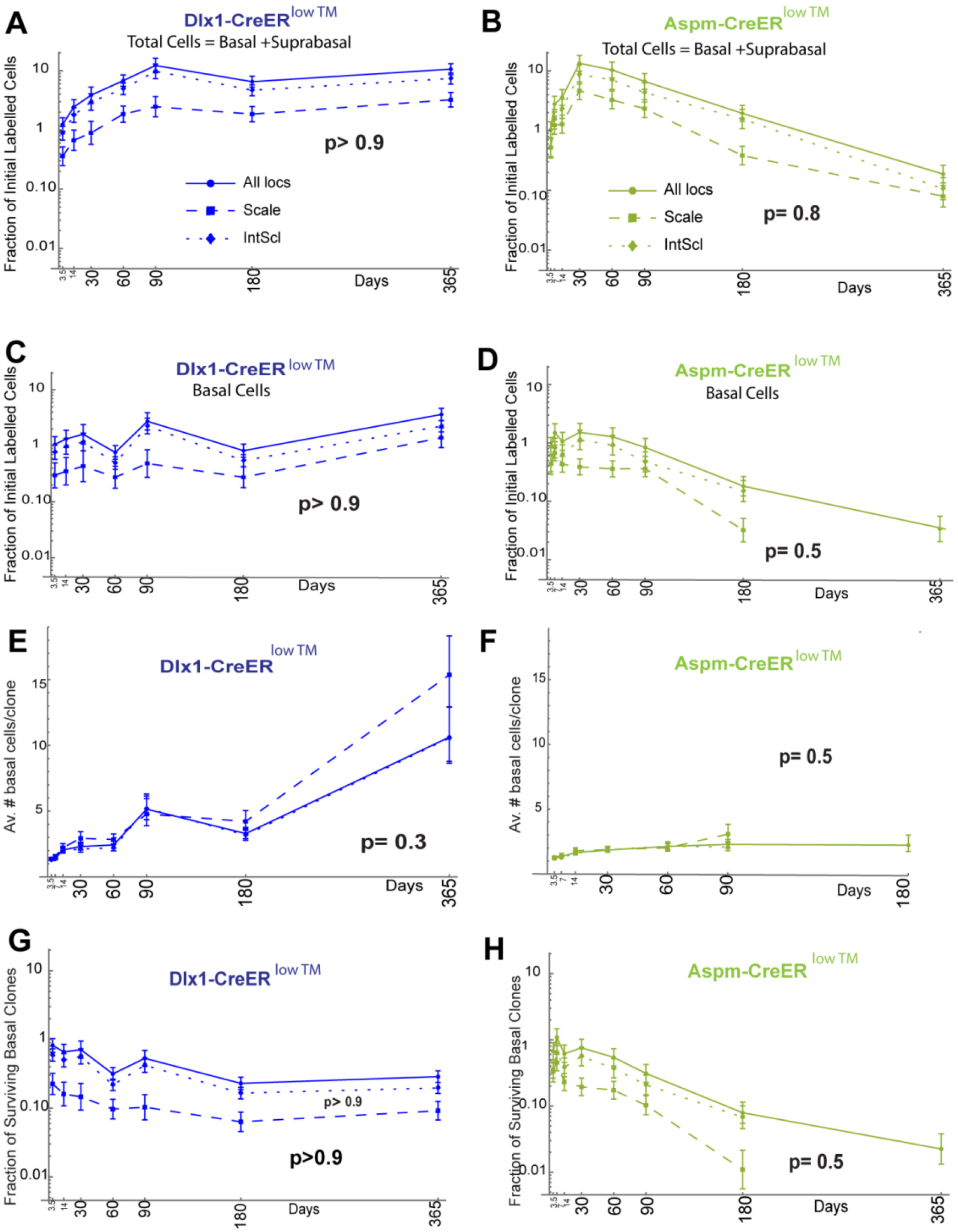

Development • Supplementary information

Fig. S5. Dlx1-CreER$^{low\ TM}$ and Aspm-CreER$^{low\ TM}$ clonal dynamics in scale and interscale. (A-H) The lineage tracing data shown in Fig. 3 is separated according to location in epidermal scale vs interscale. The profiles show that both populations exist in both domains with some preference to interscale. However, no location-specific differences in behavior were detected over the chase period for either population. The p-values test the similarity of the variation of the scale and interscale measurements over chase-time, ignoring differences in the overall numbers of cells in the two locations (supplementary Materials and Methods S2). Too few BL Aspm-CreER labeled cells and clones survived at 180 and 365 days for meaningful comparison.

Development • Supplementary information

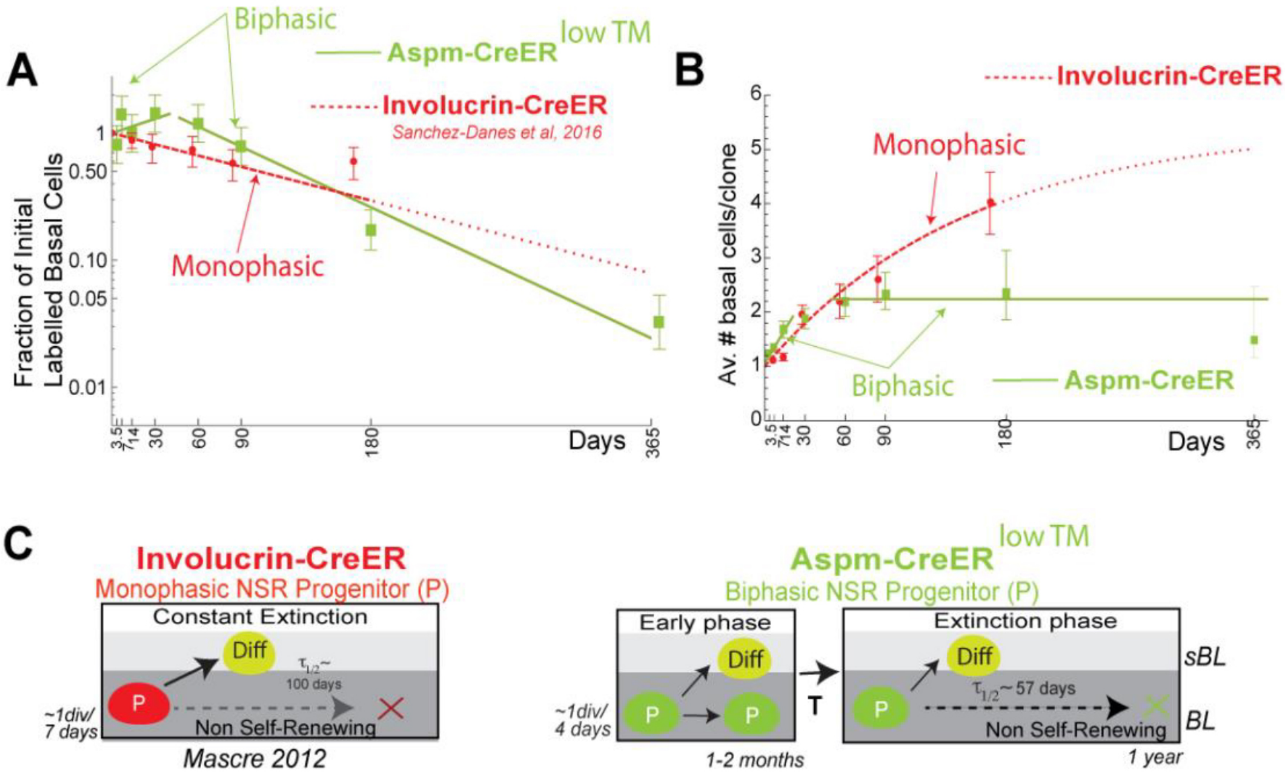

Fig. S6. Comparison of Involucrin-CreER interscale and Aspm-CreER$^{\text{low TM}}$ clonal lineage tracing. The Aspm-CreER$^{\text{low TM}}$ data and biphasic best-fits are copied from Fig. 4B. The Involucrin-CreER interscale data is from (Sanchez-Danes et al., 2016). (A) The Involucrin-CreER data (red circles) are fit to a single exponential with a best-fit terminal differentiation –renewal rate imbalance of (0.007 ± 0.003)/day excluding, as done in (Sanchez-Danes et al., 2016), the data at 180 days. In contrast, the Aspm-CreER$^{\text{low TM}}$ data (green squares) cannot be fit by a single exponential. (B) Average labeled BL clone size. The Involucrin-CreER data is well-fit by a function that relaxes exponentially with the same TD–SR rate imbalance towards its asymptotic value, as predicted for a monophasic NSR population that satisfies neutral competition without neutral drift (supplementary Materials and Methods S3). (C) Summary.

Development • Supplementary information

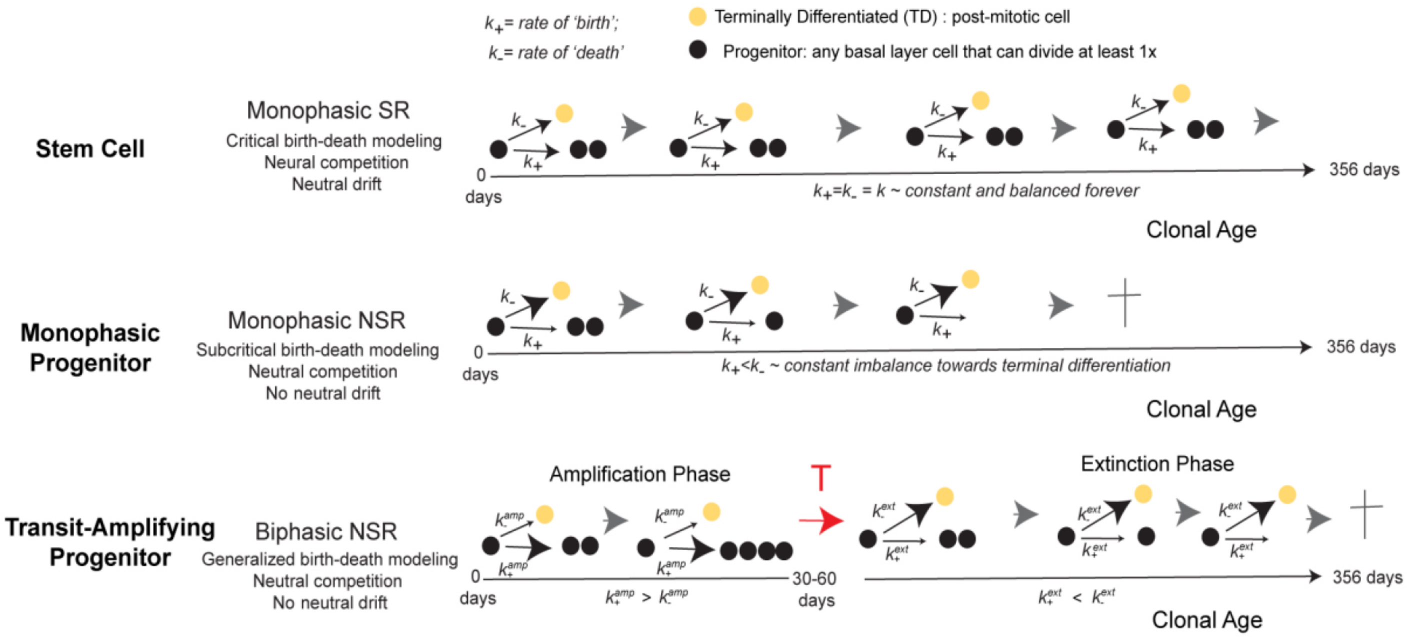

Fig. S7. Birth death modeling for three types of progenitors.

Black: dividing BL progenitor; yellow: terminally differentiated (TD) cell that is post-mitotic and would be exported to the sBL. (Top) The birth (k_+) and death (k_-) rates of monophasic self-renewing (SR) progenitors (e.g., stem cells) must be equal (e.g., balanced) for long-term maintenance of their BL numbers in homeostasis. If, in addition, the births and deaths are stochastic and independent (i.e., satisfy "neutral competition"), the changes in average labeled BL clone size and labeled BL clone survival are predicted by critical-birth death modeling to satisfy "neutral drift"—i.e., average labeled BL clone size will increase linearly and the cumulative labeled BL clone size distribution will satisfy Eq. 1 at long chase-times (Klein and Simons, 2011; Blanpain and Simons, 2013). (Middle) A monophasic NSR progenitor has constant birth and death rates favoring TD over SR. Because of this imbalance, its clones will not satisfy neutral drift and the critical birth-death model, which assumes balanced cell fates, cannot be used. However, as long as the progenitor satisfies the weaker condition of neutral competition, the "subcritical birth-death model" can be used (supplementary Materials and Methods S3). This predicts that the labeled BL cell fraction will decrease exponentially, the labeled average BL clone size will relax to an asymptotic value at the same exponential rate, and the cumulative labeled BL clone size distribution will satisfy Eq. 2 at all chase-times (supplementary Materials and Methods S3). (Bottom) A transit-amplifying (TA) progenitor is a biphasic NSR progenitor whose nascent

Development • Supplementary information

cells and their BL descendants initially have a rate imbalance favoring SR over TD in their amplification phase, which reverses to favor TD over SR after transition to their extinction phase. This progenitor's clone will not satisfy neutral drift but, as long as they evolve under neutral competition, can be modeled using the "generalized birth-death model" and will satisfy Eq. 2 at all chase-times (supplementary Materials and Methods S3).

Development • Supplementary information

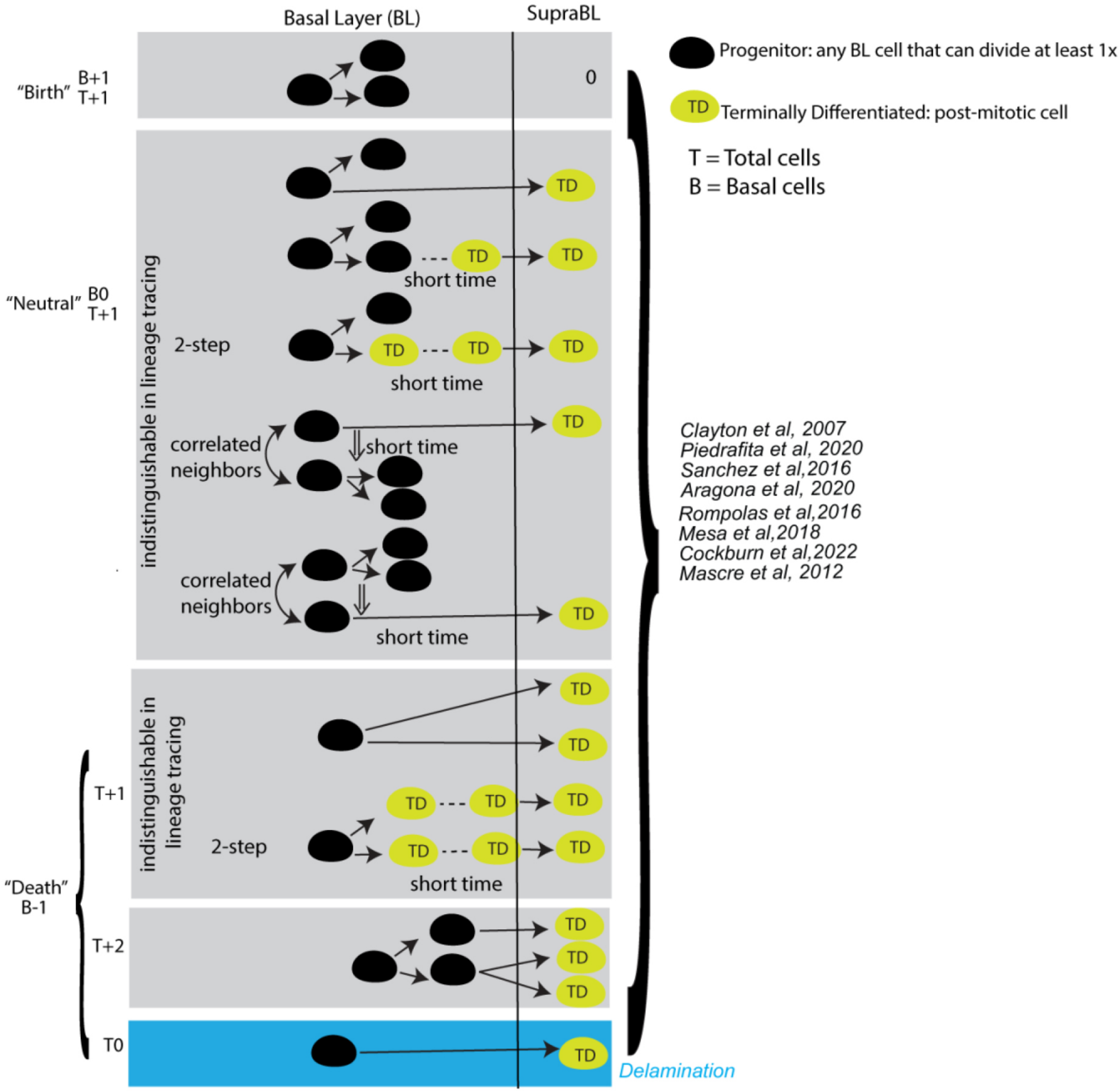

Fig. S8. Relationship between BL biological processes and births and deaths observed in lineage tracing.

BL biological events that are inferred or have been directly observed in short-term live-microscopy (Clayton et al., 2007; Mascre et al., 2012; Rompolas et al., 2016; Sanchez-Danes et al., 2016; Mesa et al., 2018; Aragona et al., 2020; Piedrafita et al., 2020; Cockburn et al., 2022) are grouped according to their effects on BL clone size that are observed in lineage tracing—i.e., birth (B+1), death (B-1), or neutral (B0) events. Correlated and sequential events that occur within the temporal

Development • Supplementary information

resolution of lineage tracing ($\gtrsim$ 1 week), appear as single events in lineage tracing and are grouped together. Biologically symmetric cell divisions (T+1 and T+2), which increase total (BL + sBL) cell fraction, can contribute to either births, deaths, or neutral lineage tracing events, while asymmetric divisions only contribute to observed neutral and death events. Terminal differentiation (TD) without division also is observed as a death event.

Development • Supplementary information

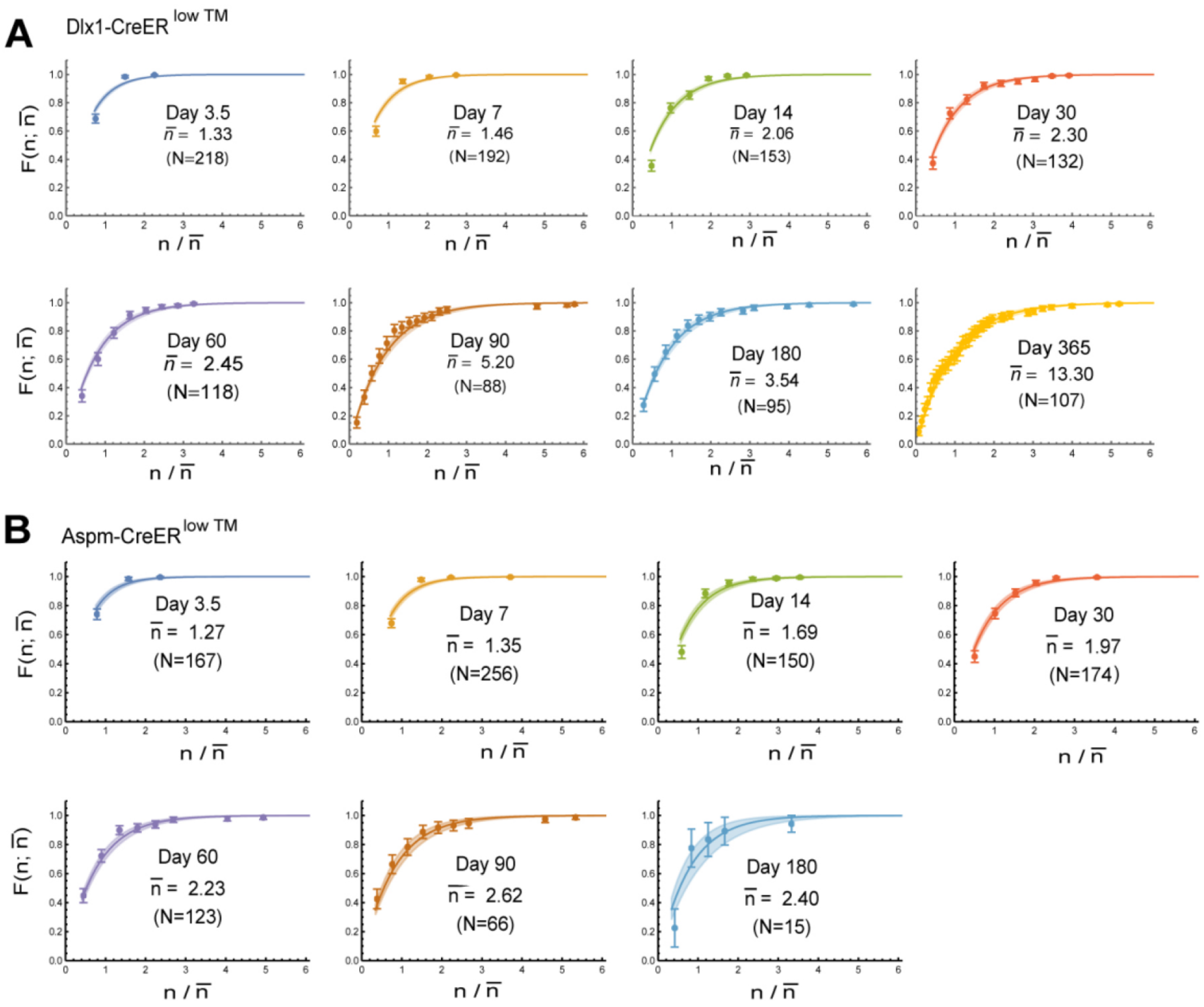

Fig. S9. Both Dlx1-CreER$^{low\ TM}$ and Aspm-CreER$^{low\ TM}$ marked clones display neutral competition at all chase times. The clone size cumulative distribution plots that are overlaid in Fig. 5B,C are displayed separately for each chase-time point. Dots are the experimental values; lines are the neutral competition predictions. The shaded regions represent the uncertainty in $\overline{n}$, which scales the abscissa. See supplementary Materials and Methods S1 and S2 for details.

Development • Supplementary information

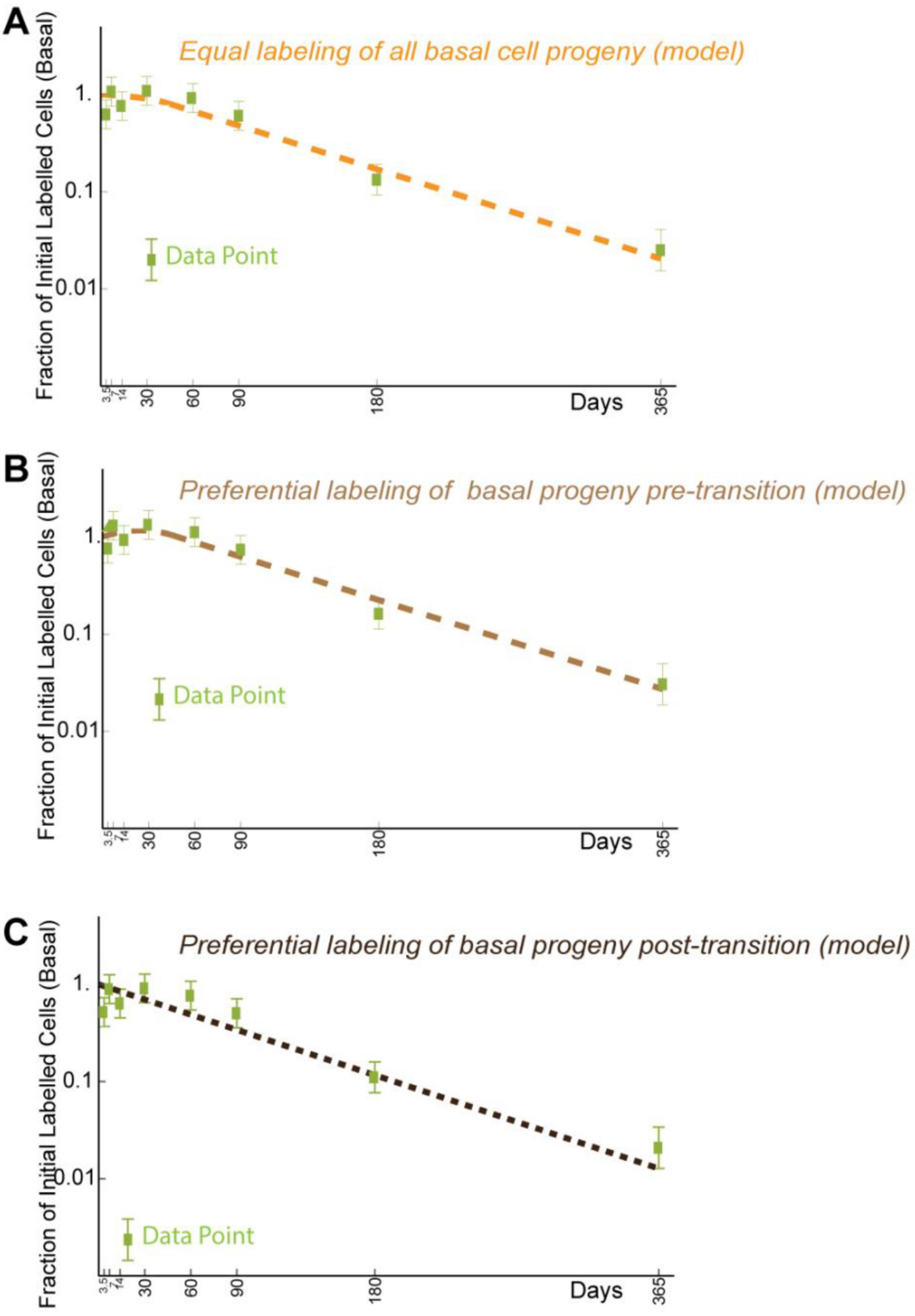
A
Equal labeling of all basal cell progeny (model)
Fraction of Initial Labelled Cells (Basal)
1.
0.1
0.01
Data Point
3.5
7
14
30
60
90
180
Days
365
B
Preferential labeling of basal progeny pre-transition (model)
Fraction of Initial Labelled Cells (Basal)
1.
0.1
0.01
Data Point
3.5
7
14
30
60
90
180
Days
365
C
Preferential labeling of basal progeny post-transition (model)
Fraction of Initial Labelled Cells (Basal)
1.
0.1
0.01
Data Point
3.5
7
14
30
60
90
180
Days
365

Fig. S10. Comparing the generalized birth-death model predictions with the experimental BL cell fraction clonal lineage tracing data for the equal-, pre-transition-, and post-transition cell-labeling models. The best-fit estimates of the birth and death rates of the pre- and post-transition cells in three models having a biological transition at a clonal age of 45 days were computed by weighted nonlinear regression of the generalized birth-death model predictions to the combination of the BL labeled cell fraction and average BL clone size data (supplementary Materials and Methods S1). Models that assumed (A) equal labeling of Aspm-CreER$^{low\ TM}$ progenitors in homeostasis, (B) only labeling of pre-transition progenitors (i.e., clone age <45 days), and (C) only labeling of post-transition progenitors (i.e., clone age >45 days) were tested. The predicted labeled BL cell fractions are compared with the experimental data (Fig. 4B) using the same rates as in the predictions of average labeled BL clone size (Fig. 5E) and nascent cell evolution (Fig. 5F). If only post-transition progenitors are labeled at the start of the experiment (panel C), the observed labeled BL cell fraction will decay exponentially at a constant rate; i.e., it will be indistinguishable in lineage tracing from a monophasic NSR progenitor.

Development • Supplementary information

Table S1. Summary of Quantitatively Analyzed Lineage Tracing Data

Aspm-CreER

	3.5 days	7 days	14 days	30 days	60 days	90 days	180 days	365 days
# Mice	4	4	4	4	4	4	3	3
# Images	5	5	5	5	5	5	5	5
# Tiles	150	150	150	150	150	150	150	150
Total # Clones	200	424	423	770	419	255	48	17
Clones Basal	167	256	147	173	123	66	15	4
Clones Suprabasal	33	168	276	597	296	189	33	13
Total Clones Scale	104	159	118	178	113	56	4	8
Total Clones Interscale	96	265	305	592	306	199	44	9

**Dlx1-CreER**	3.5 days	7 days	14 days	30 days	60 days	90 days	180 days	365 days
# Mice	3	5	3	2	5	3	5	4
# Images	5	7	4	3	7	4	8	9
# Tiles	146	211	121	85	210	115	240	276
Total # Clones	231	271	204	171	462	240	244	173
Clones Basal	218	192	153	132	118	88	95	107
Clones Suprabasal	13	79	51	39	344	152	149	66
Total Clones Scale	72	92	45	34	113	43	45	48
Total Clones Interscale	159	179	159	137	349	197	199	125

Basal clones are all clones that have at least 1 basal cell, and suprabasal clones are those with only suprabasal cells.

Table S2. Gene lists of Differentially expressed genes (DEGs) from various populations for GO analyses.

Available for download at
https://journals.biologists.com/dev/article-lookup/doi/10.1242/dev.202389#supplementary-data

Supplementary Materials and Methods

S1: Analysis of Lineage Tracing Data

All computations were performed using Mathematica (Vers. 13.2.1).

A. CALCULATION OF LABELED CELL FRACTIONS, BL CLONE SURVIVAL AND AVERAGE BL CLONE SIZES

The statistical methods described in supplementary Materials and Methods section S2 were used to compute the mean values from the raw data of Fig. 3D,E.

There was less than one labeled basal layer (BL) cell per 700 basal cells in all images at day 3.5. At this density, labeled cells that were randomly distributed in two-dimensions would be well-spaced relative to cell diameter and $\gtrsim 97\%$ of the clusters would be clonal. However, extrapolation of the best-fits of the mean labeled clone sizes (average number of labeled cells per clone) to $t = 0$ indicated that a slightly larger percentage ($\sim 5\%$) of both the Dlx1CreER$^{\text{low TM}}$ and Aspm-CreER$^{\text{low TM}}$ marked clones initially contained two labeled cells, possibly because both populations were preferentially located in the interscale, which constrained their spatial distribution. This small amount of non-clonality was unavoidable because of the need to mark a sufficient number of cells for lineage tracing. The mean clone size and clone survival best-fits included small adjustments for this.

The apparent total number of Aspm-CreER$^{\text{low TM}}$ colonies was larger at day 7 than at day 3.5 and continued to increase until day 30, after which time sBL cells were being lost by epithelial shedding. This was due to an apparent increase in the number of sBL colonies above the number of BL clones at day 3.5. Since there are no new labeled clones, this implies that the grouping of sBL cells into sBL clones and/or the grouping of sBL and BL cells into total clones could not be done accurately. Presumably this was because of rapid horizontal migration of differentiated cells in the sBL as has previously been observed (1). Therefore, we only quantitatively analyzed BL, and not total, clone survival and clone size.

Labeled cell fractions and clone survival were adjusted for the small increase in tail area with chase-time as discussed in supplementary Materials and Methods section S2 Sec. A 5.

An abnormally low number of clones were counted in some of the Dlx1-CreER$^{\text{low TM}}$ mouse images from day 7, probably because of a problem with their tamoxifen injections. Since this timepoint was closely flanked by the days 3.5 and 14, this data was not essential. Therefore, we

Development • Supplementary information

took the conservative option of not using the clone and cell count data at this timepoint. Since the clone size data was not affected,it was included.

B. EMPIRICAL BEST-FITS AND PARAMETER ESTIMATION

While the distinction between biological clones founded by nascent cells in homeostasis and the labeled clones observed in lineage tracing is important for interpreting the lineage tracing measurements (supplementary Materials and Methods section S3), since here we only discuss experimental measurements, unless otherwise noted references in this section to "clones" and "cells" refer to the labeled ones.

The analysis in this section uses only conventional stochastic modeling of dynamics in terms of division (k_{div}), self-renewal ("birth", k_+), and terminal differentiation/export ("death", k_-) rates; e.g., as in Refs. 2–4. Error-weighted best-fits to model functions were computed using two-level restricted maximum likelihood as described in supplementary Materials and Methods section S2, Sec. D.

1. BL and total labeled cell fractions

Relative BL and total cell fractions and their normalization-invariant covariance matrices at the chase timepoints t_a, $f_{\text{BL}}(t_a)$ and $f_{\text{tot}}(t_a)$, respectively, were computed as described in supplementary Materials and Methods S2, Sec. A.

a. Dlx1-CreER$^{low\ TM}$

The Dlx1-CreER$^{\text{low TM}}$ BL relative cell fractions $f_{\text{BL}}(t_a)$ increased for the first $20-30$ days, probably because of transient accumulation of non-self renewing (NSR) progeny until their steady-state fraction in the BL was reached. The stability after this indicates that Dlx1-CreER$^{\text{low TM}}$ marks a self renewing (SR) lineage. $f_{\text{BL}}(t)$ was modeled using a shifted logistic function

$$f_{\text{BL}}(t) = 1 + \tanh(k_{\text{NSR}}\, t/\Delta f_{\text{BL}})\, \Delta f_{\text{BL}}\,,$$

where $f_{\text{BL}}(0) = 1$, $d f_{\text{BL}}(t)/dt|_{t=0} = k_{\text{NSR}}$ is the rate of creating NSR BL progeny, and $1 + \Delta f_{\text{BL}}$ is the asymptote of $f_{\text{BL}}(t)$ as $t \to \infty$. The best-fit parameters were $k_{\text{NSR}} = (0.031 \pm 0.004)$/day and $\Delta f_{\text{BL}} = 0.7 \overset{\times}{\div} 2.4$.

Development • Supplementary information

Before a significant fraction of cells were shed from the epidermal surface ($0 \le t \le 30$ days), the Dlx1-CreER$^{\text{low TM}}$ total cell fraction $f_{\text{tot}}(t)$ was modeled using

$$\frac{df_{\text{tot}}(t)}{dt} = k_{\text{div}} f_{\text{BL}}(t) \tag{1a}$$

$$f_{\text{tot}}(0) = f_{\text{BL}}(0) = 1\,. \tag{1b}$$

The best-fit was $k_{\text{div}} = (0.08 \pm 0.02)$/day, corresponding to a mean division time of $1/k_{\text{div}} = 13 \pm 4$ days. Because both $f_{\text{BL}}(t)$ and $f_{\text{tot}}(t)$ include an increasing number of Dlx1-CreER$^{\text{low TM}}$ NSR progeny, k_{div} is a composite of the division rates of both Dlx1-CreER$^{\text{low TM}}$ SCs and its descendants. By $t = 60$ the rate of epithelial shedding matched the rate of BL $\rightarrow$ sBL export and $f_{\text{tot}}(t)$ reached its asymptote $f_{\text{tot}}^{\infty} = 8.6 \pm 1.3$.

b. *Aspm-CreER$^{low\ TM}$*

The abrupt changes in the Aspm-CreER$^{\text{low TM}}$ marked cell and clone dynamics at the transition from early to extinction phase that occurred between 30 and 60 days implies that the average imbalance between the rates of self-renewal and terminal differentiation of the marked BL cells is different in these phases. Therefore, the data in these experimental phases were independently fit to exponential functions $f_{\text{BL}}(t) = c\,e^{-\Delta k\,t}$, where

$$\Delta k = k_{-} - k_{+}$$

denotes the average death$-$birth BL cell rate imbalance.

Because the temporal resolution was limited, we calculated the early phase rate imbalance in two different ways—using either the data for $t \le 30$ or for $t \le 60$. These gave $\langle \Delta k \rangle|_{t\le 30} = -(0.014 \pm 0.017)$/day and $\langle \Delta k \rangle|_{t \le 60} = -(0.004 \pm 0.007)$/day, respectively. The early phase fit shown in Fig. 4B uses the weighted mean $\Delta k^{\text{early}} = -(0.01 \pm 0.01)$/day. The extinction phase rate imbalance, computed for $t \ge 60$, was $\Delta k^{\text{ext}} = (0.012 \pm 0.002)$/day. This corresponds to a half-life of $\tau_{1/2}^{\text{ext}} = 57 \pm 9$ days.

The division rate in early phase $k_{\text{div}}^{\text{early}}$ was estimated from the BL and total cell fractions for $t \le 30$, during which period the increase was still close to linear, using Eqs. 1 with $f_{\text{BL}}(t) = e^{-\Delta k^{\text{early}}\,t}$. This gave $k_{\text{div}}^{\text{early}} = (0.24 \pm 0.06)$/day, corresponding to a mean division time of 4 ± 1 days. In

extinction phase, the total cell fraction exponential decay rate, (0.013 ± 0.001)/day, was constant and essentially the same as Δk^{ext}. The constancy and equality (up to experimental accuracy) of the BL and total cell decay rates indicates that the extinction phase division rate was constant.

2. BL mean labeled clone size and survival

Mean BL clone sizes and their standard error intervals at the chase timepoints t_a, $\bar{n}(t_a)$, were computed from the data as described in supplementary Materials and Methods S2, B. The BL clone log-survivals $\log P_{\text{srv}}(t_a)$ and their error covariance matrix were computed as described in supplementary Materials and Methods S2, A. $P_{\text{srv}}(t) = f_{\text{BL}}(t)/\bar{n}(t)$ was not fit independently because it is a completely dependent function of the rela-tive cell fraction and mean clone size.[1]

a. *Dlx1-CreER$^{low\ TM}$*

As specified by the critical birth-death model (2, 5, 6), the birth=death rate k of the Dlx1-CreER$^{\text{low TM}}$ marked lineage was computed by weighted linear regression to the $\bar{n}(t_a)$ for $t \geq 30$; i.e., after $f_{\text{BL}}(t)$ had reached steady-state. This value, $k = (0.018 \pm 0.009)$/day, is similar to the birth$-$death rate imbalance of the mixture of epidermal progenitors labeled using the ubiquitous EYFP-CreER marker, $k = (0.016 \pm 0.01)/\text{day}$ (2), but is $\sim 10\times$ larger than that of the SR K14-CreER marked progenitor, $k \approx 0.0014$/day (3). Comparing this with $k_{\text{div}} = (0.08 \pm 0.02)$/day shows that, like those SR progenitors, only a small fraction of the divisions are births.

b. *Aspm-CreER$^{low\ TM}$*

At the start of the experiment almost of all the marked clones were singlets and their terminal differentiation (deaths) just removed them from the clone count without affecting the observed mean clone size. Therefore, the initial linear increase in $\bar{n}(t)$ observed for $t \leq 14$ was determined primarily by the mean birth-rate the labeled cells k_+^{early}.[2] Weighted linear regression gave $k_+^{\text{early}} \approx d\bar{n}(t)/dt|_{t \leq 30} = (0.04 \pm 0.01)$/day.

[1] We treated $P_{\text{srv}}(t)$ as the dependent variable because (1) relative cell fractions were more accurately estimated because there were more cells than clones, and (2) mean clone sizes were more accurately estimated because they did not depend on variations in efficiency of labeling the targeted cells, which was probably the largest source of error.

[2] See supplementary Materials and Methods S3, Sec. B 2 c.

Development • Supplementary information

Even though the number of clones was decreasing exponentially (Fig. 4D), the mean clone size in extinction phase was approximately constant $\bar{n}^{\mathrm{ext}} = \bar{n}|_{t\geq 60} = 2.2 \pm 0.02$. This value depends on the imbalance between births and deaths, and equilibrium kinetics implies that $\bar{n}^{\mathrm{ext}} = k_{-}^{\mathrm{ext}}/\Delta k^{\mathrm{ext}}$.[3] Combining this with the extinction phase rate imbalance Δk^{ext} (see above) gave $k_{+}^{\mathrm{ext}} = 0.015^{+0.005}_{-0.003}$/day and $k_{-}^{\mathrm{ext}} = 0.027^{+0.005}_{-0.004}$/day. k_{+}^{ext} is about $2-3\times$ smaller that k_{+}^{early}, implying that a decrease in the self-renewal rate is at partly responsible for the change in rate imbalance at the transition.[4]

C. GENERALIZED BIRTH-DEATH MODELING OF THE ASPM-CreER$^{\mathrm{low\ TM}}$ BL LINEAGE TRACING DATA

1. Biological and labeled cell clones

Maintenance of the cell fraction of a NSR progenitor in homeostasis requires the constant input of *nascent* BL cells by differentiation from a SR progenitor.[5] Each nascent cell founds a *biological clone* of BL descendants that is eventually extinguished because the rate of terminal differentiation exceeds that of self-renewal. Therefore, NSR homeostasis depends on a constant flux of developing clones whose *biological clonal age (BCA)*—the time since its nascent cell ancestor was introduced—keeps increasing, and the homeostatic population is a mixture of cells of different BCAs. This mixture is labeled at the start of an experiment, and labeled cell founds a *labeled cell clone* whose BCA at the time of labeling increases with chase-time.

The behavior of this labeled cell clone mixture will match that of a biological clone if the growth rates (i.e., division, self-renewal, and terminal differentiation rates) of the cells do not change with development.[6] This appears to be the case for the previously described interscale Inv-CreER marked NSR progenitors whose BL fraction decreases at a constant exponential rate (Ref. 4 and Fig. S7A). However, it is not so for the Aspm-CreER$^{\mathrm{low\ TM}}$ marked cells:

---

[3] See supplementary Materials and Methods S3 Sec. B 2 d.

[4] Since, as discussed in the next section, $k_{+}^{e}arly$ is the average birth rate of a mixture of labeled BL cells that may include cells that have already passed through the biological transition, this may be an underestimate of the change in birth rate.

[5] It is also possible that the nascent cells are generated by differentiation of another NSR progenitor in a hierarchy that is ultimately founded by a SR stem cell.

[6] In principle the behaviors could match if only nascent cells were labeled. However, this is unlikely in practice (and in these experiments) because the labeling conditions are empirically adjusted to label enough cells for accurate quantitation. This requires that cells having a significant range of BCAs are labeled.

Development • Supplementary information

The transition that occurs between to 30 to 60 days after tamoxifen injection (Fig. 4) implies that the rate imbalance of these biological clones must change as they develop. This might result from internal maturation of the developing clones or their migration to a different micro-niche some time after the introduction of their nascent cell founder. In any case, the lineage tracing results report averages over a mixture of cells having different initial BCAs that increase with chase-time. A further complication is that the efficiency of tamoxifen-induced labeling might depend on the BCA of the homeostatic BL cell; this would change the relationship between the homeostatic distribution of BCAs and that of the labeled cells.

Recognizing the difference between biological and labeled cell clones and the importance of the labeling-efficiency-dependent averaging was not previously needed when analyzing lineage tracing of SR stem cells and NSR progenitors that have constant growth rates—i.e., as in the critical and subcritical birth-death modeling of Refs. 2–4. However, it is critical for determining the biological pre- and post-transition rates of the Aspm-Cre$^{\mathrm{low\ TM}}$ marked progenitors in homeostasis from the lineage tracing data. The generalization of birth-death modeling needed for this is described in detail in supplementary Materials and Methods S3 and is applied here to the Aspm-CreER$^{\mathrm{low\ TM}}$ data.

2. Inferring the biological properties of the Aspm-CreER$^{\mathrm{low\ TM}}$ progenitors in homeostasis

To allow for the possibility that the initial labeling efficiency depends on BCA, we examine three possibilities: (1) cells of all BCA were labeled equally, (2) only pre-transition cells were labeled, (3) only post-transition cells were labeled. The data shows that the transition occurs (probably gradually) between 30 and 60 days so, to avoid overparameterization, we assume it occurs abruptly at the midpoint, $\tau = 45$ days. The experimental measurements show that the rates are constant after the transition and, to avoid overparametrization, we assume that they are also constant, although different, before the transition. Therefore, the only adjustable parameters for the equal labeling and pre-transition cases are the pre- and post-transition birth and death rates, k_+^{pre}, k_-^{pre}, k_+^{post}, and k_-^{post}. If only post-transition cells are labeled, the pre-transition rates k_+^{pre} and k_-^{pre} do not affect the data. Lineage tracing data cannot distinguish this case from that of a monophasic NSR progenitor and, as in that case, there are only two relevant parameters, k_+^{post} and k_-^{post}.

The predicted labeled BL cell fraction $f_{\mathrm{BL}}(t)$, mean clone size $\bar{n}(t)$, and clone survival $P_{\mathrm{srv}}(t)$ were computed for each choice of the parameters using the methods of supplementary Materials and Methods S3, Secs. A and B for the pre-transition- and equal-labeling models,

Development • Supplementary information

and using the monophasic NSR analysis of supplementary Materials and Methods S3, Sec. C 1 for the monophasic post-transition-labeling model. Because the labeled BL cell fractions and mean clone sizes both depend on all the rate parameters, all the parameters were fit simultaneously by minimizing the combined error-weighted sums of squared errors between the experimental measurements of f_{BL} and n^{-} and their model-predicted values.[7] $P_{\mathrm{srv}}(t)$ was not included in the minimization because it is not an independent variable.

The best-fits to the mean labeled BL clone size and BL cell fraction data are shown in Figs. 5D and S11, respectively. The best-fit biological rates and biological lineage properties were

	Progenitors Labeled		
	pre-transition	equal	post-transition
labeled	$\tau \leq 45$	all	$\tau > 45$
k_{+}^{pre}	0.041	0.24	–
k_{+}^{post}	0.013	0.012	0.032
k_{-}^{pre}	0.027	0.20	–
k_{-}^{post}	0.024	0.023	0.043
Δk^{pre}	-0.014	-0.038	–
Δk^{post}	0.011	0.011	0.011
f^{pre}	0.28	0.20	–
$\langle n \rangle(45)$	1.9	5.6	–
r	0.0045	0.0017	0.011
τ_e	187	282	91
$\langle \tau \rangle$	103	112	91

where τ is measured in days and rates in inverse-days, f^{pre} is the fraction of BL cells having $\tau \leq 45$ in homeostasis (i.e., pre-transition cells), r is the fractional replacement rate during homeostasis, τ_e is the "extinction time" of a nascent cell's lineage (i.e., the time when the clone survival probability decreases to $1/e$), $\langle \tau \rangle$ is the mean lifetime of a nascent cell's lineage, and $\langle n \rangle(45)$ is the mean size of a clone founded by one nascent cell at the end of the early phase—i.e., the "amplification factor."

[7] This was the sum over all chase-times t_a of the normalization-invariant squared Mahalanobis distance between the $f_{\mathrm{BL}}(t_a)$ and their model-predicted values (supplementary Materials and Methods S2, Sec. A 6) and the inverse variance-weighted sum of squared errors between the logit-transformed $g[n^{-}(t_a)]$ and their model-predicted values (supplementary Materials and Methods S2, Sec. B 3).

Development • Supplementary information

The two-parameter monophasic post-transition-labeling model is hierarchically nested within the four-parameter biphasic pre-transition- and equal-labeling models. Therefore, we used the likelihood-ratio test to compare the goodness of fit between it and the four-parameter models taking into account the difference in the number of adjustable parameters. The p-values for $H0$, which assesses the goodness-of-fit of the post-transition-labeling/monophasic model relative to the pre-transition- and equal-labeling models (large values are good), were 0.004 and 0.006, respectively. Therefore the post-transition-labeling/monophasic model is strongly rejected, consistent with the conclusion that the Aspm-CreER$^{\text{low TM}}$ lineage is biphasic.

The best-fits to k_+^{post}, k_-^{post}, and Δk^{post} in both biphasic models matched the corresponding experimental values k_+^{ext}, k_-^{ext}, and Δk^{ext}, and in agreement with the experimental prediction, in both cases $k_+(\tau)$ decreased more than three-fold at the transition. In both models only $f^{\text{pre}} \sim 20-28\%$ of the progenitors were pre-transition in homeostasis. In either case, the predicted nascent progenitor amplification was between 1.9 to 5.6-fold, so we conclude that it is strongly likely that Aspm-CreER$^{\text{low TM}}$ marks a TA progenitor. Fig. 5E displays the predicted expectation value of the number of cells in a biological clone as BCA increases in the pre-transition- and equal-labeling models.

As discussed in supplementary Materials and Methods S3, Sec. A 2, $f_{\text{BL}}(t)$ will decrease monotonically if there is equal-labeling (Fig. S11A) even though the number of nascent progenitor descendants increases $\sim$ 5.9-fold during amplification phase (Fig. 5E). While the best-fit log-slope of the experimental $f_{\text{BL}}(t)$ was positive (Fig. 4B), a small negative log-slope and, correspondingly, equal-labeling, are not excluded within experimental error.

Development • Supplementary information

S2: Statistical Methods

All experiments were independently performed at least twice with $n \geq 2$ mice (Supplementary Table 1) and representative data are shown. Sample sizes were dictated by experimental considerations and not by a statistical method. The experiments were not randomized. The investigators were not blinded to allocation and outcome assessment during the experiments.

While the distinction between biological clones founded by nascent cells in homeostasis and the labeled clones observed in lineage tracing is important for interpreting the lineage tracing measurements (supplementary Materials and Methods S3), since here we focus on the statistical analysis of experimental mea-surements, unless otherwise noted references in this supplementary Materials and Methods to "clones" and "cells" are to the labeled ones.

A. ESTIMATING RELATIVE CELL AND CLONE FRACTIONS

Summary: Image counts at individual measurement timepoints were overdispersed relative to Poisson statistics, presumably because of physiological variations between images and both physiological variations and variations in tamoxifen-induced labeling efficiency between mice. Within individual experiments (i.e., in which mice were contemporaneously injected with tamoxifen) at each timepoint the overdispersion between the pooled images from all the replicate mice was not significantly larger than that between the images from individual mice. This indicated that inter-image variations were dominant. Therefore, data from the replicate mice within individual experiments were pooled. In addition to the stochastic variations, the data indicated that there were differences between the overall labeling efficiencies in the Dlx1-CreER$^{\text{low TM}}$ (but not the Aspm-CreER$^{\text{low TM}}$) experiments that affected all timepoints coordinately. Therefore, the labeling efficiencies in the Dlx1-CreER$^{\text{low TM}}$ experiments were treated as nuisance parameters and marginalized to estimate the density ratios between timepoints. Finally, the estimated ratios were adjusted for the small increase in tail area with mouse age to compute the relative cell and clone fractions at the timepoints.

Development • Supplementary information

1. Two-level hierarchical estimation of mean labeled cell and clone densities using the compound Poisson-Gamma distribution in a single experiment.

We first discuss the estimation of cell densities. Except as noted, the same methods were used to estimate clone densities.

We denote the count of labeled cells in and the area of the ith image in mouse m at timepoint a as n_i and s_i, respectively, and denote the corresponding dataset as $D^{(a,m)} = \{(n_i, s_i) : 1 \leq i \leq N^{(a,m)}\}$. The probability of observing n_i labeled cells in an image of area s_i sampled from an epidermal region with mean density of labeled cells η_i is given by the Poisson distribution $\mathcal{P}$:

$$p(n_i|s_i,\eta_i) = \mathcal{P}(n_i; s_i\eta_i) = \frac{(\eta_i s_i)^{n_i} e^{-s_i\eta_i}}{n_i!}. \tag{1}$$

The maximum likelihood estimator (MLE) of η_i is $\hat{\eta}_i = n_i/s_i$. The differences between the $\hat{\eta}_i$ within individual datasets $D^{(a,m)}$ were much larger than expected from the corresponding Poisson standard errors, indicating that the data was overdispersed, presumably due to biological variations between images in each mouse. This precluded the use of Poisson statistics alone for estimation.

Instead we used a two-level hierarchical model that accounted for both biological dispersion and counting statistics. Overdispersion was not increased by pooling the data from replicate mice, which indicated that inter-image variations were more important than inter-mouse variations. Therefore, we pooled the images at timepoint a to create the dataset $D^a = \cup_m D^{(a,m)}$ for analysis.

In the first level of the model, image i $(1 \leq i \leq N^a = \sum_m N^{(a,m)})$ having area s_i is randomly selected at chase-time t^a from the epidermis, which has a varying labeled cell spatial density with overall mean $\bar{\eta}^a$. The probability that the labeled cell density within image i is η_i is given by a Gamma distribution, the conjugate prior to the Poisson distribution:[8,9]

$$p(\eta_i|\bar{\eta}^a, k) = \Gamma(\eta_i, \bar{\eta}^a, k) = \frac{(\eta_i/\bar{\eta}^a)^{k-1} e^{-k\eta_i/\bar{\eta}^a} k^k}{\bar{\eta}^a \Gamma(k)} \qquad (k > 0, \bar{\eta}^a > 0).$$

The scaled standard deviation is $\sigma_\eta/\bar{\eta}^a = 1/k$; therefore, the "shape factor" k parameterizes the overdispersion. We expect that the biological factors that determine the shape of the distribution are the same at every timepoint even though the scale set by $\bar{\eta}^a$ varies. Therefore, to avoid

[8] For convenience, we have parameterized the Gamma distribution in terms of $\bar{\eta}^a$ and k; the conventional parameters are k and $\theta = \bar{\eta}/k$.

[9] When the meaning is obvious from context, we overload the notation for probability distributions $p(\cdot)$ and log-likelihoods $\mathcal{L}(\cdot)$.

Development • Supplementary information

overfitting we assumed that k is the same for all a.

In the second level, image i is counted, giving n_i counts with the Poisson probabilities of Eq. 1. Marginalizing over the latent variable η_i gives

$$\begin{aligned} p(n_i|s_i,\bar{\eta}^a,k) &= \int_0^\infty \mathcal{P}(n_i;s_i\eta_i)\,\Gamma(\eta_i;\bar{\eta}^a,k)\,d\eta_i \\ &= \frac{(s_i\bar{\eta}^a/k)^{n_i}\,\Gamma(k+n_i)}{(1+s_i\bar{\eta}^a/k)^{k+n_i}\,n_i!\,\Gamma(k)} \\ &= \mathcal{P}\text{-}\Gamma(n_i;s_i\bar{\eta}^a,k)\,, \end{aligned}$$

where $\mathcal{P}$-Γ is the Poisson-Gamma compound distribution using our parametrization. When the biological dispersion is small, $\lim_{k\to\infty}\mathcal{P}\text{-}\Gamma(n;s\bar{\eta},k) = \mathcal{P}(n;s\bar{\eta})$ as expected.

The log-likelihood of $\bar{\eta}^a$ and k, conditioned on data D^a, is the sum of the individual image log-probabilities:

$$\begin{aligned} \mathcal{L}_\ell^{\bar{\eta}}(\bar{\eta}^a,k|D^a) &= \sum_{i=1}^{N^a} \log \mathcal{P}\text{-}\Gamma(n_i;s_i\bar{\eta}^a,k) \\ &= \sum_i^{N^a}\left[n_i\log(s_i\bar{\eta}^a/k) - (k+n_i)\log(1+s_i\bar{\eta}^a/k) + \log\left(\frac{\Gamma(k+n_i)}{n_i!}\right)\right] - N^a\log\Gamma(k)\,, \end{aligned} \tag{2}$$

where the subscript ℓ emphasizes that the likelihood is over labeled cell parameters.

Probably because most of the sources of estimation error (e.g., differences in labeling efficiency and biological dynamics) induce multiplicative variations in the $\bar{\eta}^a$, the $\mathcal{L}_\ell^{\bar{\eta}}(\bar{\eta}^a,k|D^a)$ were approximately log-normal. Therefore, we reexpressed Eq. 2 using the log-mean labeled cell density variables $\bar{\lambda}_\ell^a$:

$$\begin{aligned} \mathcal{L}_\ell(\bar{\lambda}_\ell^a,k|D^a) &= \mathcal{L}_\ell^{\bar{\eta}}(e^{\bar{\lambda}_\ell^a},k|D^a) \\ \bar{\lambda}_\ell^a &= \log\bar{\eta}^a\,. \end{aligned} \tag{3}$$

k appears in the log-likelihood for each D^a, so we must estimate it and the $\{\bar{\lambda}^a\}$ together using the log-likelihood conditioned on the complete experimental dataset $D = \cup_a D^a$.

$$\mathcal{L}_\ell(\{\bar{\lambda}_\ell^a\},k|D) = \sum_a \mathcal{L}_\ell(\bar{\lambda}_\ell^a,k|D^a)\,. \tag{4}$$

Development • Supplementary information

2. Combining data from multiple experiments

If the fractions of targeted cells that were initially labeled following tamoxifen injection—the *labeling efficiencies*—were equal for all the mice in the experiments, then

$$\bar{\lambda}_\ell^a = \bar{\lambda}^a + \varphi\,, \tag{5}$$

where $\bar{\lambda}^a$ is the fraction of targeted cells at chase timepoint a and φ is the log-(labeling efficiency). In this case, the difference between the target cell log-densities at timepoints a and b would equal the difference between the corresponding labeled cell log-densities:

$$\bar{\lambda}^a - \bar{\lambda}^b = \bar{\lambda}_\ell^a - \bar{\lambda}_\ell^b\,.$$

However, when there are N^x experiments involving the injection of different groups of mice at different times, the average log-(labeling efficiencies) φ^x ($i = 1 \ldots N^x$) may differ between experiments. If we could use a different overdispersion parameter k for each experiment, we could estimate the $\bar{\lambda}_\ell^a - \bar{\lambda}_\ell^b$ separately in each experiment and then combine the estimates. However, this would overparameterize the data because the individual experiments did not have enough data for good estimation of k. Moreover, we expect the random biological factors responsible for the overdispersion to be the same in all experiments, so the same k should be used. Instead, we introduced the φ^x as nuisance variables, estimated the joint likelihood of the $\bar{\lambda}^a$ and the φ^x conditioned on the data from all these experiments, and marginalized over the φ^x to estimate the $\bar{\lambda}^a - \bar{\lambda}^b$.

Significant differences between the φ^x were not observed in the Aspm-CreER$^{\text{low TM}}$ experiments. Therefore, we pooled the data from all the experiments and used the log-likelihood of Eq. 4 for analysis. However, since most subprocedures were the same in both situations, for brevity we first describe the case of different labeling efficiencies and then treat equal $\varphi^x = \varphi$ $(\forall x)$ as a special case.

a. *Likelihood of targeted cell log-mean densities*

Defining $\bar{\lambda}_\ell^{(a,x)}$ as the labeled cell log-mean density at timepoint a observed in experiment x,

$$\bar{\lambda}_\ell^{(a,x)} = \bar{\lambda}^a + \varphi^x\,.$$

Development • Supplementary information

Therefore, denoting $D^{(a,x)}$ as the data at timepoint a in experiment x and $D^x = \cup_a D^{(a,x)}$ as the complete data from experiment x, the log-likelihood is

$$\mathcal{L}(\bar{\lambda}^a, \varphi^x, k|D^x) = \mathcal{L}_\ell(\bar{\lambda}_\ell^{(a,x)} - \varphi^x, k|D^x)\,,$$

where the right-hand-side is defined by Eq. 3 with the substitutions $\bar{\lambda}_\ell^a \to \bar{\lambda}_\ell^{(a,x)} - \varphi^x$ and $D \to D^x$. The log-likelihood for all the parameters conditioned on the combined data $D = \cup_x D^a$ is

$$\begin{aligned}
\mathcal{L}(\{\bar{\lambda}^a\}, \{\varphi^x\}, k|D) &= \sum_{a,x} \mathcal{L}(\bar{\lambda}^a, \varphi^x, k|D^x) \\
&= \sum_{a,x} \mathcal{L}_\ell(\{\bar{\lambda}^a + \varphi^x\}, k|D^x) = \sum_{(a,x)} \sum_{i=1}^{N^{(a,x)}} \log \mathcal{P}\text{-}\Gamma(n_i; s_i e^{\bar{\lambda}^a + \varphi^x}, k) \\
= \sum_{(a,x)} \sum_{i}^{N^{(a,x)}} \Bigg[n_i^{(a,x)} \log\left(s_i^{(a,x)} e^{\bar{\lambda}^a + \varphi^x}/k\right) &- (k + n_i^{(a,x)}) \log\left(1 + s_i^{(a,x)} e^{\bar{\lambda}^a + \varphi^x}/k\right) + \log\left(\frac{\Gamma(k + n_i^{(a,x)})}{n_i^{(a,x)}!}\right)\Bigg] - \\
&N \log\Gamma(k)\,,
\end{aligned}$$

where $N^{(a,x)}$ is the number of measurements at timepoint a in experiment x and $N = \sum_{a,x} N^{(a,x)}$.

b. Removing the degeneracy

$\mathcal{L}(\{\bar{\lambda}^a\}, \{\varphi^x\}, k|D)$ is degenerate because it is invariant under the transformation

$$\bar{\lambda}^a \to \bar{\lambda}^a + c,\ \varphi^x \to \varphi^x - c;\ (\forall a, x)\,, \tag{6}$$

which corresponds to an unobservable correlated change in all of the mean targeted cell densities with a compensating inverse change in all the labeling efficiencies. This reflects the fact that the absolute target cell densities are unknown and only differences between their ratios are meaningful. Without loss of generality, we remove the degeneracy by using φ^{N^x} as a normalization reference and use the $N^a + N^x - 1$ differences $\{\Delta\bar{\lambda}^a = \bar{\lambda}^a - \varphi^{N^x} : 1 \leq a \leq N^a)$ and $\{\Delta\varphi^x = \varphi^x - \varphi^{N^x} : 1 \leq x \leq N^x - 1\}$ as independent variables. For convenience, we define the shifted N^a- and $(N^x - 1)$-

Development • Supplementary information

dimensional vectors

$$\Delta\bar{\boldsymbol{\lambda}} = (\bar{\lambda}^1 - \varphi^{N^x}, \bar{\lambda}^2 - \varphi^{N^x}, \ldots, \bar{\lambda}^{N^a} - \varphi^{N^x}) \tag{7a}$$

$$\Delta\boldsymbol{\varphi} = (\varphi^1 - \varphi^{N^x}, \varphi^2 - \varphi^{N^x}, \ldots, \varphi^{N^x-1} - \varphi^{N^x}), \tag{7b}$$

and define

$$\mathcal{L}(\Delta\bar{\boldsymbol{\lambda}}, \Delta\boldsymbol{\varphi}, k|D) = \mathcal{L}(\{\bar{\lambda}^a\}, \{\varphi^x\}, k|D)\big|_{\varphi^{N^x}=0}\,.$$

c. *Bayesian maximum marginal likelihood estimation*

While we could simply use the MLE and Hessian of $\mathcal{L}(\Delta\bar{\boldsymbol{\lambda}}, \Delta\boldsymbol{\varphi}, k|D)$ to estimate $\Delta\bar{\boldsymbol{\lambda}}$ and its covariance matrix, we can get better estimates of $\Delta\bar{\boldsymbol{\lambda}}$ and $\Delta\boldsymbol{\varphi}$ using their natural uninformative priors to compute their Bayesian posterior.

i. Uninformative priors for $\Delta\bar{\boldsymbol{\lambda}}$ *and* $\Delta\boldsymbol{\varphi}$. The independence of the $\bar{\lambda}^a$,[10] φ^x, and k implies that the uninformative prior $p(\{\bar{\lambda}^a\}, \{\varphi^x\}, k)$ must factorize into $\prod_a p(\bar{\lambda}^a) \times \prod_x p(\varphi^x) \times p(k)$. Moreover, the invariance of Eq. 6 and the prior exchangeability of the $\bar{\lambda}^a$ and the φ^x amongst themselves implies that each $p(\bar{\lambda}^a)$ and $p(\varphi^x)$ must individually be constant. Therefore, the appropriate (improper) priors are

$$p(\Delta\bar{\boldsymbol{\lambda}}) = 1$$
$$p(\Delta\boldsymbol{\varphi}) = 1$$
$$p(\{\bar{\lambda}^a\}, \{\varphi^x\}, k) = p(k)\,.$$

ii. Maximum marginal likelihood estimator of k. We do not know the natural uninformative prior for k so we just used its maximum marginal likelihood estimate (7)

$$\tilde{k} = \underset{k}{\operatorname{argmax}} \mathcal{L}\,(k|D)\,|_{k>0}$$
$$\mathcal{L}\,(k|D) = \log \int e^{\mathcal{L}(\Delta\bar{\boldsymbol{\lambda}}, \Delta\boldsymbol{\varphi}, k|D)}\, d\Delta\bar{\boldsymbol{\lambda}}\, d\Delta\boldsymbol{\varphi}\,. \tag{8}$$

[10] This is true for the labeled cell densities, but not strictly true for the labeled clone densities because we know *a priori* that $\bar{\lambda}^a \leq \bar{\lambda}^b$ if $a < b$. Incorporating this small amount of information would break independence and greatly complicate the analysis for little gain, so we do not do this.

Each $\mathcal{L}(k, \Delta\bar{\lambda}^a, \Delta\varphi^x | \{n_i^a, s_i^a\})$ was close to quadratic over $\Delta\bar{\lambda}^a$ and $\Delta\varphi^x$ near its maximum, so we used a quadratic approximation to evaluate the integral in Eq. 8 while computing $\tilde{k}$ numerically.

iii. The best-estimate of $\Delta\bar{\boldsymbol{\lambda}}$ *and its covariance matrix.* Because the priors over $\Delta\bar{\boldsymbol{\lambda}}$ and $\Delta\boldsymbol{\varphi}$ are constants, the posterior distribution with $k = \tilde{k}$ is

$$p(\Delta\bar{\boldsymbol{\lambda}}, \Delta\boldsymbol{\varphi} | D) \propto e^{\mathcal{L}(\Delta\bar{\boldsymbol{\lambda}}, \Delta\boldsymbol{\varphi}, \tilde{k}|D)} \,.$$

$\mathcal{L}(\Delta\bar{\boldsymbol{\lambda}}, \Delta\boldsymbol{\varphi}, \tilde{k}|D)$ was close to quadratic at the maximum *a posteriori* (MAP) estimate

$$\widetilde{\Delta\bar{\boldsymbol{\lambda}}} \oplus \widetilde{\Delta\boldsymbol{\varphi}} = \underset{\Delta\bar{\boldsymbol{\lambda}} \oplus \Delta\boldsymbol{\varphi}}{\operatorname{argmax}} \mathcal{L}(\Delta\bar{\boldsymbol{\lambda}}, \Delta\boldsymbol{\varphi}, \tilde{k}|D)) \,,$$

so we computed the marginalized covariance matrix $\Sigma_{\tilde{\boldsymbol{\lambda}}}$ from the inverse Hessian. To this end, we first combined $\Delta\bar{\boldsymbol{\lambda}}$ and $\Delta\boldsymbol{\varphi}$ into a single $(N^a + N^x - 1)$-vector $\Delta\boldsymbol{\gamma} = \Delta\bar{\boldsymbol{\lambda}} \oplus \Delta\boldsymbol{\varphi}$

$$\Delta\gamma_i = \begin{cases} \Delta\bar{\lambda}^i & (1 \le i \le N^a) \\ \Delta\varphi^{i-N^a} & (N^a + 1 \le i \le N^a + N^x - 1) \end{cases}$$

and defined $\mathcal{L}(\Delta\boldsymbol{\gamma}|D) = \mathcal{L}(\Delta\bar{\boldsymbol{\lambda}}, \Delta\boldsymbol{\varphi}|D$. $\Delta\boldsymbol{\gamma}$ has the MAP estimate and covariance matrix

$$\widetilde{\Delta\boldsymbol{\gamma}} = \widetilde{\Delta\bar{\boldsymbol{\lambda}}} \oplus \widetilde{\Delta\boldsymbol{\varphi}}$$

$$\Sigma_{\widetilde{\Delta\boldsymbol{\gamma}}} = \left(- \left. \frac{\partial^2 \mathcal{L}(\Delta\boldsymbol{\gamma}|D)}{\partial\Delta\boldsymbol{\gamma}\partial\Delta\boldsymbol{\gamma}} \right|_{\Delta\boldsymbol{\gamma}=\widetilde{\Delta\boldsymbol{\gamma}}} \right)^{-1/2} .$$

The components of $\Sigma_{\widetilde{\Delta\bar{\boldsymbol{\lambda}}}}$ are the relevant components of $\Sigma_{\widetilde{\Delta\boldsymbol{\gamma}}}$:

$$(\Sigma_{\widetilde{\Delta\bar{\boldsymbol{\lambda}}}})_{ij} = \langle (\Delta\gamma_i - \widetilde{\Delta\gamma}_i)\, (\Delta\gamma_i - \widetilde{\Delta\gamma}_j) \rangle = (\Sigma_{\widetilde{\Delta\boldsymbol{\gamma}}})_{ij} \,,$$

where $\langle \cdot \rangle$ denotes the expectation over the posterior distribution of $\Delta\boldsymbol{\gamma}$.

3. Relative labeling efficiencies of data subsets

The complete dataset $D^{\text{tot,all}}$, which includes the total (BL+sBL) counts in all locations (scale+interscale) provided the most accurate estimate of $\Delta\boldsymbol{\varphi}$. Therefore, the posterior $p(\Delta\boldsymbol{\varphi}|D^{\text{tot,all}}) =$

Development • Supplementary information

$\int p(\Delta\bar{\boldsymbol{\lambda}}, \Delta\boldsymbol{\varphi}|D^{\text{tot,all}})\, d\Delta\bar{\boldsymbol{\lambda}}$ was used to compute the posteriors over $\Delta\boldsymbol{\lambda}$ for the BL, BL scale, and BL interscale data subsets. Using D^{sub} and $\boldsymbol{\lambda}^{\text{sub}}$ to denote the restricted data subset and its parameter vector, and applying the probability chain rule gives

$$p(\Delta\bar{\boldsymbol{\lambda}}^{\text{sub}}|D^{\text{sub}}, D^{\text{tot,all}}) = \int p(\Delta\bar{\boldsymbol{\lambda}}^{\text{sub}}|D^{\text{sub}}, \Delta\boldsymbol{\varphi})\, p(\Delta\boldsymbol{\varphi}|D^{\text{tot,all}})\, d\Delta\boldsymbol{\varphi}$$
$$= \int \frac{p(\Delta\bar{\boldsymbol{\lambda}}^{\text{sub}}, \Delta\boldsymbol{\varphi}|D^{\text{sub}})}{\int p(\Delta\bar{\boldsymbol{\lambda}}, \Delta\boldsymbol{\varphi}|D^{\text{sub}})\, d\Delta\bar{\boldsymbol{\lambda}}} \left[\int p(\Delta\bar{\boldsymbol{\lambda}}, \Delta\boldsymbol{\varphi}|D^{\text{tot,all}})\, d\Delta\bar{\boldsymbol{\lambda}}\right] d\Delta\boldsymbol{\varphi}.$$

The multivariate integrals were evaluated using the quadratic approximations justified above, which reduced the integrations to linear algebra. $\tilde{\bar{\boldsymbol{\lambda}}}$ and its covariance matrix were then computed as described in Sec. A 2 c iii.

Because there were more labeled cells than clones, and hence smaller counting variances, the $p(\Delta\boldsymbol{\varphi}|D^{\text{tot,all}})$ computed from the cell fractions was also used when computing BL clone density posterior distributions. In this case, D^{sub} denotes the clone count dataset of interest.

4. Combining experiments with similar labeling efficiencies

The full machinery described above was not needed for the Aspm-CreER$^{\text{low TM}}$ experiments, since they had similar labeling efficiencies whose small variations were subsumed within the dispersion in the Poisson-Gamma distribution. Therefore, we pooled the data from all experiments and computed the Bayesian maximum marginal likelihood estimate using Eq. 4. This is equivalent to setting $N^x = 1$ and $\Delta\boldsymbol{\gamma} = \Delta\boldsymbol{\lambda}$ in the procedure described above.

5. Adjustment for increasing tail surface area

Measurements of tail diameter and length from chase-times t from 14 to 365 days showed that the mean tail epidermal surface areas of both male and female mice increased by (6 ± 0.1)%/month for the first six months of chase before stabilizing. (The mean female area was $\sim 9\%$ less than the mean male area, throughout.) The average increase over the full year of chase was $\sim 3\%$/month, in agreement with Ref. 2. The increase dilutes the measured labeled cell and clone densities and, although the effect is small compared to the biological changes, we adjusted for it. Defining the logarithm of mean tail area at chase-time a as S^a, its standard error as s_{S^a}, the N_a-vector $\boldsymbol{S} = (S_a, \ldots, S_{N^a})$, and the diagonal covariance matrix $\Sigma_{\boldsymbol{S}}$ with $(\Sigma_{\boldsymbol{S}})_{a,b} = \delta_{a,b}\,(s_{S^a})^2$, the adjusted

Development • Supplementary information

cell/clone fractions and their covariance matrix are

$$\boldsymbol{f} = \widetilde{\Delta\bar{\boldsymbol{\lambda}}} + \boldsymbol{S} \tag{9a}$$

$$\Sigma_{\boldsymbol{f}} = \Sigma_{\widetilde{\Delta\bar{\boldsymbol{\lambda}}}} + \Sigma_{\boldsymbol{S}}\,. \tag{9b}$$

6. Normalization-invariant degenerate covariance matrix

$\boldsymbol{f}$ depends on the normalization of $\widetilde{\Delta\bar{\boldsymbol{\lambda}}}$, which was arbitrarily fixed by Eq. 7a, and the units used to measure tail area. Even though this does not affect the estimates of the variables of interest, the $f^a - f^b$, it complicates the estimation of model parameters by regression. We eliminate this complication by projecting $\boldsymbol{f}$ and $\Sigma_{\boldsymbol{f}}$ into the normalization-invariant $N^a - 1$ dimensional subspace using the projection operator P:

$$\boldsymbol{f}_{\mathsf{P}} = \mathsf{P}\,\boldsymbol{f}$$

$$\Sigma_{\boldsymbol{f}_{\mathsf{P}}} = \mathsf{P}\,\Sigma_{\boldsymbol{f}}\,\mathsf{P} \tag{10}$$

$$\mathsf{P} = \mathsf{I} - \mathbf{1} \otimes \mathbf{1}/N^a\,, \tag{11}$$

where $\mathbf{1}$ is the N^a vector with all unity components. $\boldsymbol{f}_{\mathsf{P}}$ and $\Sigma_{\boldsymbol{f}_{\mathsf{P}}}$ are invariant under changes in overall normalization $\boldsymbol{f}_{\mathsf{P}} \to \boldsymbol{f}_{\mathsf{P}} + c\mathbf{1}$.

Regressions to fit the data for the plots in Fig. 4 used the normalization-invariant inverse covariance-weighted sum of squared residuals (the squared Mahalanobis distance) between the experimental values and the empirical fit functions $\boldsymbol{f}_{\text{model}}$: $(\boldsymbol{f}_{\text{model}} - \boldsymbol{f}_{\mathsf{P}})^T\ \Sigma^{+}_{\boldsymbol{f}_{\mathsf{P}}}\ (\boldsymbol{f}_{\text{model}} - \boldsymbol{f}_{\mathsf{P}})$, where $(\cdot)^{+}$ denotes the pseudoinverse. The relative cell fractions and clone survival probabilities displayed in the figures have been normalized to the fit-extrapolated values at $t = 0$. The standard errors of the f^a_{P} are the diagonal elements $\sqrt{(\Sigma_{\boldsymbol{f}_{\mathsf{P}}})_{aa}}$.

B. ESTIMATING MEAN LABELED CLONE SIZES

Summary: Differences in experimental labeling efficiency did not affect clone size, so we did not distinguish experiments for clone size analysis. The clone sizes are restricted to be ≥ 1 and their distributions were highly skewed, so using normal distribution statistics to estimate the mean clone sizes the timepoints $\bar{n}(t_a)$ would have been inaccurate. Instead, we computed likelihoods us-

Development • Supplementary information

ing the shifted geometric clone size distribution that was predicted by the generalized birth-death model (supplementary Materials and Methods S3, Sec. B) and was validated in Figs. 5B,C and S11. Applying the logit trans-form gave log-likelihoods that were close to quadratic. The differences between the mean clone size estimates from different mice were often much bigger than expected from their standard er-rors, indicating that clone sizes, like cell and clone densities, were overdispersed due to biological variations and required two-level statistical analysis. The inter-mouse variation was modeled using a Beta distribution (the conjugate prior to the geometric distribution), and the resultant compound Beta-binomial distribution was used as a two-level hierarchical model to estimate the $\bar{n}(t_a)$ using the complete dataset.

1. Shifted geometric distribution likelihood

We denote the dataset of sizes n_i of the $N^{(a,m)}$ clones measured in mouse m at timepoint a as $D^{(a,m)} = \{n_i : 1 \leq i \leq N^{(a,m)}\}$. Following supplementary Materials and Methods S3, Sec. B, we modeled the probability distribution of the n_i within $D^{(a,m)}$ as a shifted geometric distribution with success parameter $\pi = 1/\bar{n}$:

$$p(n_i;\pi) = (1-\pi)^{n-1}\pi \qquad\qquad (n_i \geq 1, 0 < \pi \leq 1)\,.$$

$T^{(a,m)} = \sum_{i=1}^{N^{(a,m)}} n_i$ is a sufficient statistics for $p(D^{(a,m)}|\pi)$, and the likelihood of π is

$$p(D^{(a,m)}|\pi) \propto p(T^{(a,m)}|\pi, N^{(a,m)}) = \binom{T^{(a,m)}-1}{N^{(a,m)}-1} (1-\pi)^{T^{(a,m)}-N^{(a,m)}} \pi^{N^{(a,m)}}\,. \qquad (12)$$

Up to the normalization factor,[11] this is a binomial distribution.

This has MLE $\hat{\pi}^{(a,m)} = N^{(a,m)}/T^{(a,m)}$ with asymptotic standard error $s_{\hat{\pi}^{(a,m)}} = \sqrt{\frac{\hat{\pi}^{(a,m)}(1-\hat{\pi}^{(a,m)})}{T^{(a,m)}}}$. However, both the $p(D^{(a,m)}|\pi)$ and the transformed likelihood over $\bar{n}$ were both far from normal distributions, implying that estimating the standard error using either $s_{\hat{\pi}^{(a,m)}}$ or the corresponding $s_{\hat{\bar{n}}^{(a,m)}}$ as parameters would be inaccurate. To get better estimates we used the canonical link of the

[11] The binomial factor does not affect the estimation and is presented only for interest. It is number of distinguishable distributions of T items (cells) into N bins (clones) (8) so that $\sum_{T=N}^{\infty} p(T|\pi,N) = 1$.

binomial distribution, the logit transform, to define

$$g(\pi) = -\log\frac{\pi}{1-\pi} = \log(\bar{n}-1) \tag{13a}$$

$$\pi(g) = \frac{1}{1+e^{g}}\,. \tag{13b}$$

This gave log-likelihoods

$$\mathcal{L}(g|D^{(a,m)}) = -T^{(a,m)}\log(1+e^{-g}) - N^{(a,m)}\,g \tag{14}$$

that were approximately quadratic, so accurate standard errors could be computed from the second-derivative at the MLE, $\hat{g}^{(a,m)}$:

$$\hat{g}^{(a,m)} = \log\left(T^{(a,m)}/N^{(a,m)} - 1\right) \tag{15a}$$

$$s_{\hat{g}^{(a,m)}} = \left(-\frac{d^2\mathcal{L}(g|D^{(a,m)})}{dg^2}\bigg|_{g=\hat{g}^{(a,m)}}\right)^{-1/2} = \sqrt{\frac{T^{(a,m)}/N^{(a,m)}}{T^{(a,m)}-N^{(a,m)}}}\,. \tag{15b}$$

2. Two-level hierarchical estimation of mean labeled clone size

Echoing the results with cell and clone densities, the differences in the $\hat{g}^{(a,m)}$ between mice were often larger than expected from the $s_{\hat{g}^{(a,m)}}$. This indicated that there were significant biological variations between the replicate mice, so a two-level hierarchical analysis was needed. To this end, we modeled the inter-mouse variation of π at each timepoint using the Beta distribution, the conjugate prior to the geometric distribution.

$$p(\pi|\alpha,\beta) = \text{Beta}(\pi;\alpha,\beta) = \frac{\pi^{\alpha-1}(1-\pi)^{\beta-1}}{B(\alpha,\beta)} \qquad (0<\alpha, 0<\beta, 0<\pi\leq 1)\,, \tag{16}$$

Development • Supplementary information

where $B(\alpha,\beta)$ is the Beta function. The $\pi^{(a,m)}$ are now latent variables. Combining Eqs. 12 and 16 with the chain rule and marginalizing over π gives the compound distribution

$$\begin{aligned} p(T|\alpha,\beta,N) &= \int_0^1 p(T|\pi,N)\, p(\pi|\alpha,\beta)\, d\pi \\ &= \binom{T-1}{N-1} \int_0^1 (1-\pi)^{T-N} \pi^N \,\mathrm{Beta}(\pi;\alpha,\beta)\, d\pi \\ &= \binom{T-1}{N-1} \frac{B(T-N+\alpha, N+\beta)}{B(\alpha,\beta)}, \end{aligned} \tag{17}$$

which is proportional to a Beta-binomial distribution.

We reparameterized this in terms of the variables of interest—$\bar{g}$ and σ_g, the mean and standard deviation of g, respectively. Using Eqs. 13,

$$\bar{g}(\alpha,\beta) = \int_0^1 g(\pi)\mathrm{Beta}(\pi;\alpha,\beta)\, d\pi = \psi(\beta) - \psi(\alpha) \tag{18a}$$

$$\sigma_g^2(\alpha,\beta) = \int_0^1 [g(\pi)-\bar{g}]^2\, \mathrm{Beta}(\pi;\alpha,\beta)\, d\pi = \psi'(\alpha) + \psi'(\beta), \tag{18b}$$

where $\psi(x) = \Gamma'(z)/\Gamma(z)$ is the digamma function. Inverting these numerically gives the implicit functions $\alpha(\bar{g},\sigma_g)$ and $\beta(\bar{g},\sigma_g)$.

Using this parametrization, the complete log-likelihood at timepoint a, conditioned on the data from all mice $D^a = \cup_m D^{(a,m)}$ is

$$\mathcal{L}(\bar{g}^a, \sigma_g^a | D^a) = \sum_m \log p\left[T^{(a,m)} \middle| \alpha(\bar{g}^a,\sigma_g^a), \beta(\bar{g}^a,\sigma_g^a), N^{(a,m)} \right].$$

If overdispersion is negligible so that $\sigma_g(\alpha,\beta) \to 0$, Eqs. 18 imply that $\lim_{\sigma_g \to 0} \mathrm{Beta}[\pi;\alpha(\bar{g},\sigma_g),\beta(\bar{g},\sigma_g)] = \delta[\pi - \pi(\bar{g})]$, so $\lim_{\sigma_g \to 0} p[T|\alpha(\bar{g},\sigma_g),\beta(\bar{g},\sigma_g),N] = p[D^{(a,m)}|\pi(\bar{g})]$ and, up to a constant

$$\lim_{\sigma_g^a \to 0} \mathcal{L}(\bar{g}^a,\sigma_g^a|D^a) = \sum_m \mathcal{L}(\bar{g}^a|D^{(a,m)}) = -\sum_m T^{(a,m)} \log(1+e^{-\bar{g}^a}) - \sum_m N^{(a,m)}\, \bar{g}^a. \tag{19}$$

This equals the (single-level) log-likelihood of the pooled dataset.

Development • Supplementary information

3. Empirical maximum likelihood estimation

The inter-mouse variations of $g^a = \log(\bar{n}^a - 1)$ that cause the overdispersion depended primarily on variations between mice of $\log k_+$ and $\log \Delta k$, where k_+ is the birth rate and Δk is the death−birth rate imbalance (see supplementary Materials and Methods S3, Eqs. 15b and 16c), and we expect these to be simi-lar at all timepoints. Therefore, to avoid overfitting we assume that the overdispersion is the same at all timepoints, i.e., ${\sigma^a}_g = \sigma_g$. The complete likelihood for the entire dataset of clone sizes in all mice at all timepoints $D = \cup_a D^a$ is

$$\mathcal{L}(\{\bar{g}^a\}, \sigma_g | D) = \sum_a \mathcal{L}(\bar{g}^a, \sigma_g | D^a)\,.$$

Because we do not have a natural uninformative prior for g, we fixed σ_g empirically at its maximizer

$$(\{\hat{\bar{g}}^a\}, \hat{\sigma}_g) = \underset{(\{\bar{g}^a\}, \sigma_g \geq 0)}{\operatorname{argmax}} \; \mathcal{L}(\{\bar{g}^a\}, \sigma_g | D)\,.$$

The individual timepoint log-likelihood functions $\mathcal{L}(\bar{g}^a, \hat{\sigma}_g | D^a)$ were close to quadratic around $\hat{\bar{g}}^a$, so the asymptotic formula for the standard error was sufficiently accurate:[12]

$$s_{\hat{\bar{g}}^a} = \left(- \left. \frac{\partial^2 \mathcal{L}\left[\bar{g}^a, \hat{\sigma}_g | D^a\right]}{\partial (\bar{g}^a)^2} \right|_{\bar{g}^a = \hat{\bar{g}}^a} \right)^{-1/2}.$$

The inverse-variance weighted sum of squared residuals between the $\hat{\bar{g}}^a$ and model predictions $g(\bar{n}^a_{\text{model}})$ were used for regression.

$\hat{\bar{g}}^a$ and $\hat{\bar{g}}^a \pm s^a_{\hat{\bar{g}}}$ were back-transformed using Eq. 13b to compute $\hat{\bar{n}}^a = 1 + e^{\hat{\bar{g}}^a}$ and its asymmetric standard error interval

$$1 + e^{\hat{\bar{g}}^a - s^a_{\hat{\bar{g}}}} = \hat{\bar{n}}^a_- \leq \hat{\bar{n}}^a \leq \hat{\bar{n}}^a_+ = 1 + e^{\hat{\bar{g}}^a + s^a_{\hat{\bar{g}}}}\,. \qquad (20)$$

C. ESTIMATING CUMULATIVE CLONE SIZE DISTRIBUTIONS

There was not enough data to compute cumulative clone size distributions for the mice separately, so the pooled datasets D^a were analyzed. If k clones of size $n' \leq n$ are observed in a

[12] Because there were only four Aspm-CreER$^{\text{low TM}}$ marked clones at day 365, we corrected its standard error using Student's t-distribution. This correction was unnecessary at the other timepoints.

Development • Supplementary information

population of N labeled clones, the likelihood that the cumulative probability of n is Π_n is

$$p(k|\Pi_n, N) = \mathrm{Bin}\left[k; \Pi_n, N\right] = \binom{N}{k} \Pi^n \left(1-\Pi\right)^{N-k},$$

where Bin is the binomial distribution. The MLE, $\Pi_n = k/N$, is highly biased when $k \ll N$ or $k \approx N$, so we did not use it. Instead, in Figs. 5B,C and S11 we display the Wilson score intervals with the recommended exact correction (9) for $k = 0$ and $k = N$ with $\alpha = 0.32$ to determine standard error bars, and indicate the mean of the interval with dots.

For consistency, the $\hat{\bar{n}}^a$ used to scale the abscissa were computed using the D^a and Eq. 19. The region corresponding to scaling with $\bar{n}$ between $\hat{\bar{n}}^a_-$ and $\hat{\bar{n}}^a_+$ in Eq. 20 is shaded in Fig. S11.

D. PARAMETER ESTIMATION USING TWO-LEVEL WEIGHTED NONLINEAR REGRESSION

The inverse-variance weighted squared residuals at adjacent timepoints were sometimes large, possibly because of variations in the efficiency of labeling, sample location, or other biological factors. Therefore, both the magnitudes of these fluctuations as well as the measurement errors needed to be incorporated when determining the relative weighting of the measurements in model fitting. To this end, linear and nonlinear regressions were performed using restricted maximum marginal likelihood random effects analysis (10). This reduces to weighted regression when the measurement errors are sufficient to explain the residuals and reduces to non-weighted regression when the additional variations are dominant. It provides conservative parameter error estimates that are bounded below by the larger of the estimates provided by weighted or unweighted regression alone.

E. P-VALUES

P-values comparing Dlx1-CreER$^{\mathrm{low\ TM}}$ and Aspm-CreER$^{\mathrm{low\ TM}}$ cell fractions, mean clone sizes, and clone survivals were computed using Hotelling's multivariate T-squared distribution. The normalization-invariant squared Mahalanobis distance (Sec. A)was used as the T-squared statistic for relative log-fraction differences; the inverse variance-weighted sum of squared differences between the logit-transformed $g[\bar{n}(t_a)]$ was used for studying mean clone size differences.

Development • Supplementary information

S3: Inferring the Biological Properties of Evolving NSR Progenitors from Lineage Tracing Data

By definition, NSR progenitor lineages require a constant input of BL *nascent* progenitors from the parental progenitor to replace the cells lost by terminal differentiation. During homeostasis each nascent progenitor founds a *biological clone* of descendants that is eventually eliminated by terminal differentiation and export to the sBL. Previously described NSR progenitors (e.g., interscale Inv-CreER marked cells) displayed an exponential loss of marked cells with increasing lineage tracing chase-time with a constant rate determined by a constant imbalance between self-renewal and terminal differentiation (3, 4). In this case, as long as labeling is random, the behavior of the *labeled cell clones* observed in lineage tracing is the same as that of the biological clones, and the measured BL cell division, self-renewal, and terminal differentiation rates equal the corresponding biological rates.

In contrast, the growth properties of a biological clone of a transient amplifying NSR progenitor change as the clone develops—e.g., because of altered mRNA expression and/or migration to different microenvironments. Soon after introduction of the nascent cell, the mean self-renewal rate within a clone exceeds the terminal differentiation rate, which results in amplification until the imbalance is reversed and extinction begins. As with NSR progenitors having a constant rate imbalance, the homeostatic population is a steady-state mixture of biological clones that have been initiated at different times by the continuous input of nascent cells. However, in this case the cells in the mixture have different growth rates and rate imbalances that depend on their *biological clone age (BCA)*—the time since the introduction of their nascent ancestor. This mixture is labeled following tamoxifen injection, initiating the labeled cell clones observed in the experiment. It is important to recognize that these clones are different from the biological clones, and the relationship between their behavior—observed over chase-time—and the biologically relevant behavior of the biological clones—which develop over BCA—can be complicated.

Previous studies have shown that, at least on the time-scale of lineage tracing measurements,[13] the self-renewal and terminal differentiation fate choices of SR BL progenitors are stochastic and independent—i.e., they satisfy cell-autonomous "neutral competition" (2, 3, 5, 6, 13, 14) Therefore, their clonal dynamics can be analyzed using stochastic "critical birth-death modeling" in

[13] Video microscopy studies indicate that there can be correlations between self-renewal and terminal differentiation and multi-step progression through terminal differentiation to BL→SL export on the time-scale of a few days (11, 12). However, these short time-scale correlated events are unresolved and appear as single events on the longer lineage time-scale.

Development • Supplementary information

which self-renewal events, which increase clone size and BL cell fraction, and called “births” and terminal differentiation events are called ”‘deaths”. This has been extensively discussed (5, 6), but the corresponding analysis for analysis for NSR lineages that satisfy neutral competition has not. To analyze this more complex type of data, we developed *generalized birth-death modeling* and used it to quantitatively analyze the Aspm-CreER$^{\text{low TM}}$ lineage tracing data.

In Sec. A we calculate the relationship between the biological BCA-dependent rate imbalance of nascent cell clones and the observed lineage tracing data; in Sec. B we extend critical birth-death modeling to the analysis of the labeled clones observed in lineage tracing data of BCA-dependent NSR lineages, in Sec. C we show how the subcritical and critical birth-death models of constant-rate, monophasic NSR lineages and SR lineages, respectively, emerge as special cases of the generalized model, and in Sec. D we extend the predicted asymptotic “scaling limit” for the clone size distributions of SR lineages that satisfy neutral to predict the clone size distributions of both NSR and SR lineages that undergo neutral competition at all chase-times.

A. VARYING BIOLOGICAL RATE IMBALANCE AND THE LABELED BL CELL FRACTIONS OBSERVED IN LINEAGE TRACING

1. Distribution of BL cell maturities in homeostasis

We denote the BCA of a biological clone as τ, the mean birth and death rates of the BL cells in the clone as $k_+(\tau)$ and $k_-(\tau)$, respectively, and the BCA-dependent death−birth rate imbalance as $\Delta k(\tau) = k_-(\tau) - k_+(\tau)$. These rates are averages across all the cells in the lineage that will include any heterologous progenitor progeny.

$\langle n \rangle(\tau)$, the expectation value of the number of cells in a biological BL clone satisfies

$$\frac{d\langle n \rangle(\tau)}{d\tau} = -\Delta k(\tau)\, \langle n \rangle(\tau) \qquad (\tau \geq 0)$$
$$\langle n \rangle(0) = 1\,,$$

where clones of size zero (i.e., that do not survive to BCA τ) are included in the average. This has

Development • Supplementary information

the solution

$$\langle n\rangle(\tau) = e^{-\rho(\tau)} \qquad (\tau \geq 0) \tag{1a}$$

$$\rho(\tau) = \int_0^{\tau} \Delta k(\tau')\, d\tau'\,. \tag{1b}$$

The constancy of the number of NSR BL cells N is maintained in homeostasis by the input of nascent cells that are differentiated from the parental progenitor of the lineage at a constant rate R. Therefore, the BL population is a steady-state mixture of biological clones founded at earlier times, and

$$N = R\int_{-\infty}^{t} \langle n\rangle(t-t')\, dt' = R\int_0^{\infty} e^{-\rho(\tau)}\, d\tau\,.$$

Because the population is NSR,

$$\rho(\tau) \to \infty \qquad (\tau \to \infty) \tag{2}$$

fast enough for convergence. The *fractional replacement rate* is

$$r = \frac{R}{N} = \frac{1}{\int_0^{\infty} e^{-\rho(\tau)}\, d\tau}\,, \tag{3}$$

the homeostatic probability density over cell BCAs is

$$p_{\mathrm{h}}(\tau) = \frac{e^{-\rho(\tau)}}{\int_0^{\infty} e^{-\rho(\tau')}\, d\tau'}\,,$$

and the mean lifetime of a nascent cells' lineage is

$$\langle \tau \rangle = \int_0^{\infty} \tau\, p_h(\tau)\, d\tau\,.$$

The "extinction time" τ_e is the solution of

$$\langle n\rangle(\tau_e) = 1/e\,.$$

Development • Supplementary information

2. Labeled BL cell fraction observed in lineage tracing

$\eta(\tau;t)$, the number of labeled BL cells of BCA τ at chase-time t, satisfies

$$\frac{\partial\eta(\tau;t)}{\partial t} = -\Delta k(\tau)\,\eta(\tau;t) - \frac{\partial\eta(\tau;t)}{\partial\tau} \qquad (\tau > 0)\,, \tag{4}$$

with boundary conditions

$$\begin{aligned} \eta(\tau_0;0) &= \eta(0)\,\xi(\tau_0)\,p_{\mathrm{h}}(\tau_0) && (0 \le \tau_0) \\ \eta(\tau;t) &= 0 && (\tau < t)\,. \end{aligned}$$

The first condition describes the initial distribution of BCAs of the labeled cells. The second reflects the fact that all labeled cells at chase-time t must have BCA $\tau \geq t$. Eq. 4 has the solution

$$\eta(\tau;t) = \eta(0)\,\theta(\tau - t)\,\xi(\tau - t)\,p_{\mathrm{h}}(\tau)\,.$$

$\eta(t) = \int_0^\infty \eta(\tau,t)\,d\tau$ is the total number of labeled BL cells at chase-time t. Relative to the number at the start of the chase,

$$\begin{aligned} \frac{\eta(t)}{\eta(0)} &= \int_t^\infty \xi(\tau - t)\,p_{\mathrm{h}}(\tau)\,d\tau \\ &= \int_0^\infty e^{-\rho^{\tau_0}(t)}\,\xi(\tau_0)\,p(\tau_0)d\tau_0 \\ &= \int_0^\infty \langle n\rangle^{\tau_0}(t)\,\xi(\tau_0)\,p(\tau_0)d\tau_0\,, \end{aligned} \tag{5}$$

where

$$\rho^{\tau_0}(t) = \rho(\tau_0 + t) - \rho(\tau_0) \tag{6a}$$

$$\langle n\rangle^{\tau_0}(t) = e^{-\rho^{\tau_0}(t)}\,. \tag{6b}$$

$\langle n\rangle^{\tau_0}(t)$ is the mean size at chase-time t, including clones of size zero, of a labeled BL clone that was founded by a cell that had BCA τ_0 when labeled at the start of the experiment. Eq. 5 expresses the relative labeled BL cell fraction at time t as the labeling efficiency-weighted integral of these mean sizes over the homeostatic distribution of the τ_0s. We call τ_0 the *labeling BCA* and call the

Development • Supplementary information

last line of Eq. 5 a *homeostasis-labeled average*.

If all homeostatic cells are equally labeled at the start of the chase, Eq. 5 reduces to

$$\frac{\eta(t)}{\eta(0)} = 1 - r\int_0^t e^{-\rho(\tau)}\,d\tau < 1\,. \qquad [\xi(\tau) = \text{constant}]\,.$$

In this case, the labeled BL cell fraction decreases monotonically at a rate corresponding to the missing contribution from the nascent cells that were not labeled as the experiment progressed. $\eta(\tau)/\eta(0)$ will decrease even if "young" cells (i.e., with small τ) have more births than deaths [i.e., $\Delta k(\tau) < 0$], because the loss of "old" cells [i.e., sufficiently large τ so that $\Delta k(\tau) > 0$] always dominates the total.

B. VARYING BIOLOGICAL BIRTH AND DEATH RATES AND THE BL CLONE SIZES AND SURVIVALS OBSERVED IN LINEAGE TRACING: THE GENERALIZED BIRTH-DEATH MODEL

As in critical birth-death modeling of SR lineages, generalized birth-death modeling of BCA-dependent NSR progenitors assumes that, up to the temporal resolution of lineage tracing, their BL clones evolve under neutral competition—i.e., the progenitors are equipotent with stochastic and independent birth and death rates. Therefore, they can be modeled as a Markov process.

1. Evolution of nascent cell BL clone sizes in homeostasis

$\pi_n(\tau)$, the probability that a biological clone of BCA τ has size n (including $n = 0$) satisfies

$$\begin{aligned}\frac{d\pi_n(\tau)}{d\tau} &= k_+(\tau)(n-1)\,\pi_{n-1}(\tau) + k_-(\tau)(n+1)\pi_{n+1}(\tau) - \\ &\quad [k_+(\tau) + k_-(\tau)]\,n\,\pi_n(\tau) \qquad \text{(7a)}\\ \pi_n(0) &= \delta_{n,1}\,. \qquad \text{(7b)}\end{aligned}$$

These BCA-dependent generalized birth-death equations hold regardless of the specific biological mechanisms responsible for the changing $k_+(\tau)$ and $k_-(\tau)$ (see Figs. 5A and S9). Kendall (15)

Development • Supplementary information

solved these equations, getting

$$\pi_0(\tau) = \frac{\zeta(\tau)}{1+\zeta(\tau)} \tag{8a}$$

$$\pi_n(\tau) = \left\{1 - e^{\rho(\tau)}/[1+\zeta(\tau)]\right\}^{n-1} \frac{e^{\rho(\tau)}}{[1+\zeta(\tau)]^2} \qquad (n \geq 1) \tag{8b}$$

$$\zeta(\tau) = \int_0^t e^{\rho(\tau')} k_-(\tau')\, d\tau' = e^{\rho(\tau)} + \int_0^t e^{\rho(\tau')} k_+(\tau')\, d\tau' \,. \tag{8c}$$

$\pi_n(\tau)$ is a shifted geometric distribution. $\pi_0(\tau)$ is the probability that a nascent cell's clone does not survive to BCA τ. As expected, the clone size expectation value computed using the $\pi_n(\tau)$ is the same as Eq. 1a:

$$\langle n \rangle(\tau) = \sum_{n=0}^{\infty} n\, \pi_n(\tau) = e^{-\rho(\tau)} \,.$$

2. Evolution of labeled BL clone sizes with chase-time

a. Labeled clones of specified labeling BCA

The BCA of the cells in a clone with labeling BCA τ_0 increases to $t+\tau_0$ at chase-time t. $\pi_n^{\tau_0}(t)$, the probability that a labeled clone of labeling τ_0 has size $n \geq 0$ at chase-time t, satisfies the modification of Eqs. 7

$$\frac{d\pi_n^{\tau_0}(t)}{dt} = k_+(\tau_0+t)(n-1)\pi_{n-1}^{\tau_0}(t) + k_-(\tau+t)(n+1)\pi_{n+1}^{\tau_0}(t) - [k_+(\tau_0+t) + k_-(\tau_0+t)]n\,\pi_n^{\tau_0}(t) \tag{9a}$$

$$\pi_n^{\tau_0}(0) = \delta_{n,1} \,. \tag{9b}$$

The solution is the modification of Eqs. 8:

$$\pi_0^{\tau_0}(t) = \frac{\zeta^{\tau_0}(t)}{1+\zeta^{\tau_0}(t)} \tag{10a}$$

$$\pi_n^{\tau_0}(t) = \left\{1 - e^{\rho^{\tau_0}(t)}/[1+\zeta^{\tau_0}(t)]\right\}^{n-1} \frac{e^{\rho^{\tau_0}(t)}}{[1+\zeta^{\tau_0}(t)]^2} \qquad (n \geq 1) \tag{10b}$$

$$\zeta^{\tau_0}(t) = \int_0^t e^{\rho^{\tau_0}(t')} k_-(\tau_0+t')\, dt' = e^{\rho^{\tau_0}(t)} + \int_0^t e^{\rho^{\tau_0}(t')} k_+(\tau_0+t')\, dt' \,, \tag{10c}$$

Development • Supplementary information

where $\rho^{\tau_0}(t) = \rho(t+\tau_0) - \rho(\tau_0)$ as defined in Eq. 6a. $\pi_n^{\tau_0}(t)$ is also a shifted geometric distribution and, as expected, its clone size expectation value has the same form as Eq. 6b:

$$\langle n \rangle^{\tau_0}(t) = \sum_{n=0}^{\infty} n\,\pi_n^{\tau_0}(t) = e^{-\rho^{\tau_0}(t)}\,.$$

The probability that a labeled clone of labeling BCA τ_0 is observed (i.e., survives with $n \geq 1$) at chase-time t is

$$P_{\mathrm{srv}}^{\tau_0}(t) = 1 - \pi_0^{\tau_0}(t) = \frac{1}{1+\zeta^{\tau_0}(t)}\,. \tag{11}$$

Therefore, the observed clone size expectation value $\bar{n}^{\tau_0}(t)$ and probabilities $p_n^{\tau_0}(t)$ are

$$\bar{n}^{\tau_0}(t) = \frac{\langle n \rangle^{\tau_0}(t)}{P_{\mathrm{srv}}^{\tau_0}(t)} = e^{-\rho^{\tau_0}(t)}\left[1+\zeta^{\tau_0}(t)\right] \geq 1 \tag{12a}$$

$$p_n^{\tau_0}(t) = \frac{\pi_n^{\tau_0}(t)}{P_{\mathrm{srv}}^{\tau_0}(t)} = \frac{\left[1-1/\bar{n}^{\tau_0}(t)\right]^{n-1}}{\bar{n}^{\tau_0}(t)} \qquad (n \geq 1) \tag{12b}$$

$$\sum_{n=1}^{\infty} p_n^{\tau_0}(t) = 1\,. \tag{12c}$$

$p_n^{\tau_0}(t)$ is also a shifted geometric distribution.

Assuming that $k_-(\tau)$ remains finite [i.e., $P_{\mathrm{srv}}(t) > 0$ for all finite t], that there are not biologically unrealistic oscillations as $t \to \infty$, and that $\lim_{t\to\infty} \Delta k(t) > 0$, it follows that

$$\lim_{t\to\infty} \bar{n}^{\tau_0}(t) = \lim_{t\to\infty} \frac{k_-(t)}{\Delta k(t)} = 1 + \lim_{t\to\infty} \frac{k_+(t)}{\Delta k(t)} \geq 1\,. \tag{13}$$

This depends on $\lim_{t\to\infty} \Delta k(t) > 0$ and does SR lineages; these are treated as a special case in Sec. C 2.

Development • Supplementary information

b. Observed mixtures of labeled clones

The observed labeled BL cell fraction, clone survival, mean clone size, and clone size probability distribution are the homeostasis-labeled averages

$$\frac{\eta(t)}{\eta(0)} = \int_0^\infty \langle n \rangle^{\tau_0}(t)\,\xi(\tau_0)\,p(\tau_0)d\tau_0 \tag{14a}$$

$$P_{\text{srv}}(t) = \int_0^\infty P_{\text{srv}}^{\tau_0}(t)\,\xi(\tau_0)\,p_{\text{h}}(\tau_0)\,d\tau_0 \tag{14b}$$

$$\bar{n}(t) = \sum_{n=1}^{\infty} n p_n(t) = \frac{\int_0^\infty \langle n \rangle^{\tau_0}(t)\,\xi(\tau_0)\,p_{\text{h}}(\tau_0)\,d\tau_0}{P_{\text{srv}}(t)} = \frac{\eta(t)}{\eta(0)\,P_{\text{srv}}(t)} \tag{14c}$$

$$p_n(t) = \frac{\int_0^\infty \pi_n^{\tau_0}(t)\,\xi(\tau_0)\,p_{\text{h}}(\tau_0)\,d\tau_0}{P_{\text{srv}}(t)} \qquad (n \geq 1)\,. \tag{14d}$$

c. Small t

Expanding Eqs. 14 in a Taylor's series,

$$\eta(t)/\eta(0) = 1 - \langle \Delta k \rangle^{\text{HL}} t + O(t^2) \tag{15a}$$

$$\bar{n}(t) = 1 + \langle k_+ \rangle^{\text{HL}} t + O(t^2) \tag{15b}$$

$$P_{\text{srv}}(t) = 1 - \langle k_- \rangle^{\text{HL}} t + O(t^2) \tag{15c}$$

$$p_n(t) = \begin{cases} 1 - \langle k_+ \rangle^{\text{HL}} t + O(t^2) & n = 1 \\ \langle k_+ \rangle^{\text{HL}} t + O(t^2) & n = 2 \\ O(t^2) & n > 2 \end{cases} \tag{15d}$$

where $\langle \Delta k \rangle^{\text{HL}}$, $\langle k_+ \rangle^{\text{HL}}$, and $\langle k_- \rangle^{\text{HL}}$ are the homeostatic labeled (HL) expectations of the respective growth parameters at the start of the chase:

$$\langle \Delta k \rangle^{\text{HL}} = \int_0^\infty k(\tau_0)\,\xi(\tau_0)\,p_h(\tau_0)\,d\tau_0 \qquad (k \in \{\Delta k, k_+, k_-\})\,.$$

If the homeostatic cells are equally labeled [i.e., $\xi(\tau_0) = \text{const}$], these are just the averages of the biological rates in homeostasis. In this case, $\langle \Delta k \rangle^{\text{HL}} = r$, the fractional replacement rate specified in Eq. 3.

Development • Supplementary information

d. Large t asymptotic behavior

If $k_+(\tau) = k_+^\infty$ and $\Delta k(\tau) = \Delta k^\infty$ for $\tau > T$, then using Eqs. 6, 10–12, and 14 the $t \gg T$ asymptotic behaviors are

$$\frac{\eta(t)}{\eta(0)} \propto e^{\Delta k^\infty (t-T)} \tag{16a}$$

$$P_{\rm srv}(t) \propto e^{\Delta k^\infty (t-T)} \tag{16b}$$

$$\bar{n}^\infty \sim 1 + k_+^\infty / \Delta k^\infty \tag{16c}$$

$$p_n(t) \sim \frac{(1-1/\bar{n}^\infty)^{n-1}}{\bar{n}^\infty} \tag{16d}$$

$$[t \gg T,\ k_+(\tau > T) = k_+{}^\infty,\ \Delta k(\tau > T) = \Delta k^\infty]\,. \tag{16e}$$

C. MONOPHASIC NSR AND SR LINEAGES: THE SUBCRITICAL AND CRITICAL BIRTH-DEATH MODELS

When a lineage is monophasic—i.e., the birth and death rates and labeling efficiency do not depend on BCA, τ and τ_0 are irrelevant, Eq. 4 reduces to a first-order differential equation, and the solution of Eq. 9 can be directly obtained using generating functions or other simple methods. However, it is informative to see how the solutions emerge as special cases of the generalized birth-death model.

1. Monophasic NSR progenitors with constant rate imbalance; the subcritical birth-death model

If k_+, k_-, and ξ are constant with $k_- - k_+ = \Delta k > 0$,

$$\rho(t) = \rho^{\tau_0}(t) = \Delta k t$$

$$\zeta(t) = \zeta^{\tau_0}(t) = \frac{e^{\Delta k t} - 1}{\Delta k}\,,$$

Development • Supplementary information

and Eqs. 14 reduce to

$$\frac{\eta(t)}{\eta(0)} = e^{-\Delta k t} \tag{17a}$$

$$\bar{n}(t) = 1 + (1 - e^{-\Delta k t})(\bar{n}^{\infty} - 1) \tag{17b}$$

$$\bar{n}^{\infty} = 1 + \frac{k_+}{\Delta k} \tag{17c}$$

$$P_{\text{srv}}(t) = \frac{1}{1 + \frac{k_-}{\Delta k}\left(e^{\Delta k t} - 1\right)} \tag{17d}$$

$$p_n(t) = \frac{[1 - 1/\bar{n}(t)]^{n-1}}{\bar{n}(t)} \qquad (n \geq 1)\,. \tag{17e}$$

This is the *subcritical birth-death model*. In this case, the biological rates can be directly inferred from the observed rate: $\eta(t)$ decays exponentially with rate Δk, and $\bar{n}(t)$ exponentially relaxes to its asymptotic value $\bar{n}^{\infty}$ with rate Δk. These equations were used in Fig. S7 to fit the BL cell fractions and mean clone sizes of Inv-CreER marked cells measured by Sánchez-Danés et. al. (4).

2. SR progenitors with balanced rates: the critical birth-death model

In this case, k_+, k_-, and ξ are constant with $k_- - k_+ = \Delta k = 0$. Taking the limit $\Delta k \to 0$ of Eqs. 17 gives

$$\frac{\eta(t)}{\eta(0)} = 1 \tag{18a}$$

$$\bar{n}(t) = 1 + kt \tag{18b}$$

$$P_{\text{srv}}(t) = \frac{1}{1 + kt} \tag{18c}$$

$$p_n(t) = \frac{[1 - 1/\bar{n}(t)]^{n-1}}{\bar{n}(t)} \qquad (n \geq 1)\,. \tag{18d}$$

This is the well-known critical birth-death model (5, 6).

D. NEUTRAL COMPETITION AND NEUTRAL DRIFT

If the birth and death rates are constant, i.e., as for SR or monophasic NSR progenitors, the labeled clone size probability distribution is the shifted geometric distribution shown in Eqs. 17e

Development • Supplementary information

and 18d. The corresponding cumulative distribution is

$$F_{n' \leq n}(n;\bar{n}) = \sum_{n'=1}^{n} p_n(t) = 1 - \left(1 - \frac{1}{\bar{n}}\right)^n . \qquad (19)$$

This is Eq. 2 in the main text and depends only on n and $\bar{n}$ with no explicit dependence on t. It is a hallmark of cell-autonomous "neutral competition"—independent, random birth and deaths of equipotent cells—and provide a test of the applicability of a Markovian birth-death model to BL clone dynamics.

If $\bar{n}$ becomes large, as it does for an SR lineage and for an NSR lineage having $\bar{n}^{\infty} \gg 1$, the cumulative distribution reaches the asymptotic form

$$F(n;\bar{n})_{n' \leq n} \sim 1 - e^{-n/\bar{n}} . \qquad (\bar{n} \gg 1) . \qquad (20)$$

This familiar "scaling limit" (Eq. 1 in the main text) only depends on the ratio $n/\bar{n}$. It, but not Eq. 19, has previously been derived from the critical birth-death model and, together with the linear increase of $\bar{n}(t)$ and inverse decrease of $P_{\text{srv}}(t)$, is a characteristic of neutral drift in lineage tracing (2–6).

When the birth and death rates depend on BCA, the $p_n(t)$ will be averages over mixtures of clones with different labeling BCAs and Eq. 19 will not be exact. However, it will hold at long chase times when Eq. 16d holds, and will also be an excellent approximation as long as the dispersion of $\bar{n}^{\tau_0}(t)$ is not large. This was the case for the Aspm-CreER$^{\text{low TM}}$ marked clones (Figs. 5C and S10B) and may be the case for other biphasic transit-amplifying cells because, as exemplified by the Aspm-CreER$^{\text{low TM}}$ modeling, the majority of labeled cells will either have similar labeling BCAs (pre-transition cell-labeling, Fig. 5E) or will be post-transition with $\bar{n}^{\tau_0}(t) = \bar{n}^{\infty}$ (equal-labeling model).

Eq. 19 is more general than Eq. 20 because it holds as long as clone fate is determined by neutral competition, even if the mean clone size never grows large. It also provides a stricter test of neutral competition than Eq. 20 since it can be tested at all chase-times and is always an exact in the large t limit, even when $\bar{n}^{\infty}$ is not large and the scaling limit is not reached.

Development • Supplementary information

REFERENCES

1. S. Ro and B. Rannala, Evidence from the stop-EGFP mouse supports a niche-sharing model of epidermal proliferative units, Exper. Dermatol. **14**, 838 (2005).
2. E. Clayton, D. Doupé, A. Klein, D. Winton, B. Simons, and P. Jones, A single type of progenitor cell maintains normal epidermis, Nature **446**, 185 (2007).
3. G. Mascre, S. Dekoninck, B. Drogar, K. Youssef, S. Brohee, P. Sotiropoulou, B. Simons, and C. Blanpain, Distinct contribution of stem and progenitor cells to epidermal maintenance, Nature **489**, 257 (2012).
4. A. Sánchez-Danés, E. Hannezo, J.-C. Larsimont, M. Liagre, K. Youssef, B. Simons, and C. Blanpain, Defining the clonal dynamics leading to mouse skin tumour initiation, Nature **536**, 298 (2016).
5. A. Klein and B. Simons, Universal patterns of stem cell fate in cycling adult tissues, Development **138**, 3103 (2011).
6. C. Blanpain and B. Simons, Unravelling stem cell dynamics by lineage tracing, Nat. Rev. Mol. Cell Biol. **14**, 489 (2013).
7. J. O. Berger, B. Liseo, and R. L. Wolpert, Integrated likelihood methods for eliminating nuisance parameters, Statist. Sci. **14**, 1 (99).
8. W. Feller, *An Introduction to Probability Theory and Its Applications*, Vol. 1 (John Wiley & Sons, 1957) pp. 36–37.
9. J. Zar, Biostatistical analysis (Prentice Hall, 2010) pp. 543–546, Fifth ed.
10. S. Raudenbush, Chapter 16, analyzing effect sizes: random-effects models, in *The Handbook of Research Synthesis and Meta-analysis*, edited by H. Cooper, L. Hedges, and J. Valentine (Russell Sage Foundation, 2009) pp. 295–315.
11. P. Rompolas, K. Mesa, K. Kawaguchi, S. Park, D. Gonzalez, S. Brown, J. Boucher, A. Klein, and V. Greco, Spatiotemporal coordinal of strem cell commitment during epidermal homeostasis, Science **352**, 1471 (2016).
12. K. Cockburn, K. Annusver, D. G. Gonzalez, S. Ganesan, D. P. May, K. R. Mesa, K. Kawaguchi, M. Kasper, and V. Greco, Gradual differentiation uncoupled from cell cycle exit generates heterogeneity in the edimermal stem cell layer, Nature Cell Biol. **24**, 1692 (2022).
13. X. Lim, S. H. Tan, W. L. C. Koh, R. M. W. Chau, K. S. Yan, C. J. Kuo, R. van Amerongen, A. M. Klein,

Development • Supplementary information

and R. Nusse, Interfollicular epidermal stem cells self-renew via autocrine Wnt signaling, Science **342**, 1226 (2013).

14. G. Piedrafita, V. Kostiou, A. Wabik, B. Colon, F. Fernandex-Antoran, A. Herms, K. Murai, B. Hall, and P. Jones, A single-progenitor model as the unifying paradigm of epidermal and esophageal ephithelial maintenance in mice, Nature Comm. **11**, 1 (2020).
15. D. Kendall, On the generalized “birth-and-death” process, Ann. Math. Statist. **19**, 1 (1948).
