# Supplementary material for: A transit-amplifying progenitor with biphasic behavior contributes to epidermal renewal
Source: Development. 2024 Jun 27;151(12):dev202389. doi: 10.1242/dev.202389 (PMC11234368; doi:10.1242/dev.202389)
Supplement: Supplementary file 1 [file develop-151-202389_review_history.pdf]

A transit-amplifying progenitor with biphasic behavior contributes to epidermal renewal

Sangeeta Ghuwalewala, Kevin Jiang, Sara Ragi, David Shalloway and Tudorita Tumbar
DOI: 10.1242/dev.202389

Editor: Hanna Mikkola

Review timeline
Original submission: 2 October 2023
Editorial decision: 17 November 2023
First revision received: 2 April 2024
Editorial decision: 7 May 2024
Second revision received: 14 May 2024
Accepted: 20 May 2024

Original submission

First decision letter

MS ID#: DEVELOP/2023/202389

MS TITLE: A Transit-amplifying progenitor with biphasic behavior contributes to epidermal renewal

AUTHORS: Sangeeta Ghuwalewala, Kevin Jiang, Sara Ragi, David Shalloway, and Tudorita Tumbar

I have now received all the referees' reports on the above manuscript, and have reached a decision. The referees' comments are appended below, or you can access them online: please go to BenchPress and click on the 'Manuscripts with Decisions' queue in the Author Area.

As you will see, the referees express considerable interest in your work, but have some significant criticisms and recommend a substantial revision of your manuscript before we can consider publication. If you are able to revise the manuscript along the lines suggested, which may involve further experiments, I will be happy receive a revised version of the manuscript. Your revised paper will be re-reviewed by one or more of the original referees, and acceptance of your manuscript will depend on your addressing satisfactorily the reviewers' major concerns. Please also note that Development will normally permit only one round of major revision. If it would be helpful, you are welcome to contact us to discuss your revision in greater detail. Please send us a point-by-point response indicating your plans for addressing the referees' comments, and we will look over this and provide further guidance.

Please attend to all of the reviewers' comments and ensure that you clearly highlight all changes made in the revised manuscript. Please avoid using 'Tracked changes' in Word files as these are lost in PDF conversion. I should be grateful if you would also provide a point-by-point response detailing how you have dealt with the points raised by the reviewers in the 'Response to Reviewers' box. If you do not agree with any of their criticisms or suggestions please explain clearly why this is so.

Reviewer 1

Advance summary and potential significance to field

© 2024. Published by The Company of Biologists under the terms of the Creative Commons Attribution License (https://creativecommons.org/licenses/by/4.0/).

Reviewer comments DEVELOP/2023/202389:

This manuscript from Gjuwalewala et al., characterize and model the clonal behavior of Aspm-labelled basal cells in the epidermis. The authors suggest that Aspm-targeted basal cells represent a distinct transit amplifying (TA) cell population in the epidermis, distinct from previous reports using quantitative lineage tracing. This work is a continuation of earlier work from the Tumbar lab, pinpointing molecular identities of slow- and fast-cycling epidermal basal cells. Identifying the molecular identity of an epidermal TA-cell population would significantly impact our understanding of epidermal tissue maintenance.

Comments for the author

Characterization of Aspm-labelled cells:
Overall, I find it a missed opportunity not to characterize the identify of Aspm-positive cells better. The authors have single-cell data from tail basal cells (Figure 1) which would allow them to address several important questions:

Are Aspm-labelled cells a distinct progenitor/stem cell population or does Aspm-expression reflect a certain cell state i.e. do all cells express Aspm when in G2/M? How are Dlx1+ Ki67+ or Slc1a3+Ki67+ cells distinct from Aspm+Ki67+ cells? How are Aspm+Ki67+ and Aspm+Ki67- cells different etc etc?

The Monocle analysis (Figure S1F) likely demonstrates that Ki67+ cycling cells are at the root of epidermal differentiation. By regressing out cell cycle genes, the authors could probe if the argument holds for Aspm.

If Aspm-expressing cells are highly proliferative, injection of EdU into Aspm-CreER and Dlx1-CreER could demonstrate the differences in EdU incorporation in reporter-positive cells. This comparison would be more convincing than the single-cell data correlation and also serve to support the computational estimation of proliferation.

If Aspm-labelling marks a specific cell population rather than a cell state, repetitive weekly injections of tamoxifen at high doses would be expected not to increase the % of labelled basal cells.

Is it not surprising that so many cells express Aspm protein using immunofluorescence (Figure S1C)? Comparing to the single-cell data, Aspm protein would be expected to correlate to Ki67+ basal cells? A direct protein correlation (Ki67/Aspm) would be information, again to verify the proliferative nature of the Aspm+ cells.

Lineage tracing and modelling:
The authors compare total clone sizes and basal clone sizes from Dlx1-CreER and Aspm-CreER lines. The observation that the Aspm-derived clones are different from Dlx1-derived clones is very interesting.

Aspm-basal clones never exceed an average of 2 basal cells, and the total clone size expansion is largely due to an increased number of labelled suprabasal cells. The authors argue that Aspm+ cells are neutrally competitive, which would imply stochastic fate choice and equal probabilities of generating a basal progenitor and basal committed cell; in contrast to the clone size compositions that are reported by the authors.
Analyzing the total clone compositions at early tracing time points could serve to understand if fate choices are altered when comparing Dlx1 and Aspm clones. Similarly, double tracing with Tam/Edu (24 hours) could shed light on if the fate choices of Dlx and Asmp daughter cells are indeed stochastic or if Asmp-daughters are skewed toward a specific fate. In other systems, Aspm is suggested to regulate symmetric fate choice.

The argument that there are “old” and “new” TA cells in the in vivo epidermis is hard to experimentally verify with the data at hand, and as such this part of the modelling becomes largely speculative.

© 2024. Published by The Company of Biologists under the terms of the Creative Commons Attribution License (https://creativecommons.org/licenses/by/4.0/).

Figure 2 could display Aspm-clones and not Dlx1-clones. Illustrating the shape of the Aspm-clones would be informative and highlight their suprabasal contribution. The graphs (Figure 2D-E as well as similar graphs in supplement) are also somewhat miss leading when all clones are displayed without normalization. Average (basal, suprabasal) clone sizes (as in Figure 3) would be more illustrative and allow for statistical comparison between animal cohorts. The Dlx1-clone size data from 7 days seems to be missing (2D-E).

The clonal analysis shows that almost all clones are lost after 365 days of tracing (Figure 3), data which is not recapitulated by the FACS analysis in Figure S1, where the % of labelled tail skin only drops from 7.8 (1 day) to 4.7 (1 year). The discrepancy between the two forms of analysis is concerning and would need to be clarified.

The clonal data also suggest that Aspm-derived clones are different depending on if they localize to scale or interscale (Figure S3). How would this impact the modelling which takes all these clones into account? Can the authors uncouple the effect on location from cell intrinsic properties?

The wounding model is interesting, but the design of the experiment leaves more to ask:

The authors suggest that both Dlx1-derived and Aspm-derived cells contribute to wound healing. The quantifications are done by quantifying the reporter+ area in the wound, which would likely correlate or at least reflect also to the starting labelling efficiency of the two lines. This data would need to be included to be able to interpret the result.

Furthermore, it would be interesting to characterize the basal contribution of Dlx1 and Aspm in wound healing. During epidermal homeostasis, Aspm-derived progeny is almost exclusively suprabasal – is this pattern changed upon wounding?

The timeline of tail wounding should be extended to see if Aspm-derived cells indeed undergo an “extinction phase” as suggested by the authors.

It is hard to make any conclusions of the back skin wounding model, since characterization of Aspm+ cells during homeostasis is only demonstrated in the tail. In addition, similar back skin wounding experiments in the Aspm-line are already published from the group (Kang, Stem Cell Reports, 2020).

N=2 is too little to allow for statistical analysis and would need to be expanded.

Reviewer 2

Advance summary and potential significance to field

The authors have been able to identify the elusive epidermal self-renewing transit-amplifying progenitor population in vivo and the interesting finding from this study was the biphasic behavior exhibited by the TA progenitor population by using genetic lineage tracing and mathematical modeling.

Comments for the author

Overall, the article was well-written though it might be best if the authors avoided extensive abbreviations; for example, BL could be written as basal layer and TD as terminal differentiation. The abbreviations are distracting and would make it difficult for a non-skin scientist to understand.
The authors demonstrate that the Aspm population was highly proliferative with more than 90% of the cells in S/G2/M in the mouse skin (Fig 1E). On the other hand, in Fig S1B the immunofluorescence staining of human skin with Ki67 and Aspm does not demonstrate a significant colocalization of the two proteins. An image with a higher resolution might be more convincing.
Fig 2D and E is a quantification of tdTomato+ clones in Aspm-CreER and Dlx1-CreER lineage traced mice. There seems to be a datapoint missing for the Dlx1-CreER clones on day 7.
The switch between proliferation to extinction phase is highly interesting, but the authors could have delved a little more into the mechanism behind the switch.

© 2024. Published by The Company of Biologists under the terms of the Creative Commons Attribution License (https://creativecommons.org/licenses/by/4.0/).

For Fig. S1H the authors did perform flow cytometry to assess the percentage of tdTomato+ population from Aspm-CreER, they could have flow sorted that fraction of cells from different timepoints to assess the transcriptomic or epigenetic changes that occur during the shift from proliferation to extinction.

Reviewer 3

Advance summary and potential significance to field

The manuscript by Ghuwalewala et al aims to understand the biphasic behavior of Transit-amplifying progenitor which contributes to epidermal renewal. Here, the authors use multiple lineage tracing experiments with mathematical modelling and single -cell RNA sequencing to identify the TA progenitor with biphasic behavior. By using the clonal analysis and modelling authors identified the non-self-renewing biphasic TA progenitor which might be important to understand their contribution in tissue repair and homeostasis.
The manuscript is clearly written, and the data well presented. The experiments appear to be well conducted. Overall, the results are nicely explained and discussed thoroughly with appropriate literature form the field. This manuscript will appeal the broad readership of Development. Below are few minor comments to further improve the quality of the paper.

Comments for the author

Several aspects that might improve the manuscript:
1. The authors showed that “Aspm+ cells were highly enriched in proliferative Ki67 cells”. It will be nice to show the other markers which are specifically expressed in the Aspm+ cluster and explain how these markers are different and important in functions apart from all cell cycle G2-M markers.
2. The authors mentioned that “Aspm-CreER marks a novel biphasic NSR population that initially divides rapidly in an early growth phase before a subsequent transition to an extinction phase”. It would be interesting to discuss about the mechanism behind this biphasic behavior.
3. In Figure 4, authors showed the Wounding response from SR and non-SR populations in tail vs back skin and how these Aspm and K14 derived cells behave differently in tissue repair. It is important to understand the contribution of basal and suprabasal cells in wounding response specifically from Aspm derived cells. Is there any specificity in contribution in tissue repair for basal vs suprabasal cells form Aspm lineage?

First revision

Author response to reviewers' comments

We thank all the reviewers for all the time and effort to review our paper and for providing thoughtful and constructive criticism, which we address point by point below. This has greatly enhanced the quality of our manuscript.

Reviewer 1 Advance Summary and Potential Significance to Field:

This manuscript from Gjuwalewala et al., characterize and model the clonal behavior of Aspm-labelled basal cells in the epidermis. The authors suggest that Aspm-targeted basal cells represent a distinct transit amplifying (TA) cell population in the epidermis, distinct from previous reports using quantitative lineage tracing. This work is a continuation of earlier work from the Tumbar lab, pinpointing molecular identities of slow- and fast-cycling epidermal basal cells. Identifying the molecular identity of an epidermal TA-cell population would significantly impact our understanding of epidermal tissue maintenance.

Reviewer 1 Comments for the Author:

© 2024. Published by The Company of Biologists under the terms of the Creative Commons Attribution License (https://creativecommons.org/licenses/by/4.0/).

Characterization of Aspm-labelled cells:
Overall, I find it a missed opportunity not to characterize the identify of Aspm-positive cells better.
We have now characterized the Aspm+ population from scRNAseq data and added new panels to Figure 1, as well as new supplementary data in Figure S1 and S2. The data added is detailed below underneath each point.

The authors have single-cell data from tail basal cells (Figure 1) which would allow them to address several important questions:
Are Aspm-labelled cells a distinct progenitor/stem cell population or does Aspm-expression reflect a certain cell state i.e. do all cells express Aspm when in G2/M?
Sub-setting the G2/M basal population demonstrates that only 47% of these cells express Aspm (Figure 1E). This is now mentioned in the text 137-138

How are Dlx1+ Ki67+ or Slc1a3+Ki67+ cells distinct from Aspm+Ki67+ cells?
The Aspm+ basal cells show increased features of proliferative cells when compared with the rest of the basal cells (Figure 1F). When Aspm+/Ki67+ were compared with Dlx1+/Ki67+ (Figure 1G), we found enrichment of several distinct pathways (Figure 1G).

Because we do not focus on the Slc1a3 in this paper we worried that partly characterizing it here would be distracting.

Figure and text removed at authors' request

How are Aspm+Ki67+ and Aspm+Ki67- cells different etc etc?

Comparing Aspm+/ki67+ vs Aspm+/Ki67- basal populations shows they are distinct cell states (Figure S1F,G). Not surprisingly Aspm+/Ki67+ cells are enriched in cell cycle and proliferation pathways. Interestingly, the Aspm+/Ki67- cells show enrichment in intermediate filaments organization and epidermis differentiation pathways.

The Monocle analysis (Figure S1F) likely demonstrates that Ki67+ cycling cells are at the root of epidermal differentiation. By regressing out cell cycle genes, the authors could probe if the argument holds for Aspm.

We thank the reviewer for this suggestion, which revealed a new dimension to our study that we previously had not sufficiently developed (see below). Unfortunately, we were no longer able to utilize pseudotime analysis with Monocle 2, since these genomic tools have not been updated and the field moved to Monocle 3 in the meantime. Therefore, we also moved to Monocle 3, regressed the cell cycle genes, and utilized the newly updated lineage prediction tools (Figure S2 A,B). Strikingly, w/o cell cycle genes the Aspm cells become redistributed among the other clusters, with new clusters 4&7 showing the highest fraction of Aspm+ cells while cluster 5 contained the highest Aspm-expressing cells (S2B&C). Cluster 4 is now predicted at the root of the lineage hierarchy. The Aspm+ cells in the 3 clusters are molecularly distinct (Fig. S2D). This new analysis suggests that within the Aspm population, there may be multiple sub-populations, some putative stem cells (in cluster 4) and other potentially more committed in cluster 5 (the one with the highest Aspm-expressing cells) and cluster 7. This prediction is in fact nicely corroborated by our lineage tracing results, at different tamoxifen induction doses. Specifically, injecting low (clonal) dose TM that may selectively target Aspm^{high} expressing cells via induction of the Aspm-CreER uncovered a subset of committed basal progenitors that do not self-renew in the long-term and behave as TA cells (Figures 3-5). In contrast, high TM dose (2x or 5x injections) that likely capture not only Aspm^{high} but also some Aspm^{low} cells results in long-term labeling during homeostasis, as expected of this population containing some stem cells (new Figure 2 and S3A). Wound healing in tail skin also uncovered differences in the Aspm vs Dlx marked population behavior when injecting mice with high TM vs low TM (Figure 2E,F). Overall, the data suggests heterogeneity in the Aspm+ basal population, which contains both stem and TA cells with the latter likely being enriched in the Aspm^{high} expressing cells. This data and discussion is now added to the paper.

© 2024. Published by The Company of Biologists under the terms of the Creative Commons Attribution License (https://creativecommons.org/licenses/by/4.0/).

If Aspm-expressing cells are highly proliferative, injection of EdU into Aspm-CreER and Dlx1-CreER could demonstrate the differences in EdU incorporation in reporter-positive cells. This comparison would be more convincing than the single-cell data correlation and also serve to support the computational estimation of proliferation.

Our initial finding that led to the discovery of Aspm and Dlx1 in epidermis was that Aspm was upregulated in the H2B-GFP non-label retaining cells while Dlx1 was present in label retaining cells (Sada et al, 2016). This indicated that Aspm-expressing cells divide more frequently. Our subsequent clonal labeling and careful quantification of basal and suprabasal progeny evolution over time (this work) clearly demonstrate that the Dlx1 marked population divides ~1x/13 days while the Aspm-marked population does so every ~4 days, an ~ 3x difference in division rates (Figure 4). Quantitative analysis of clonal data is much more sensitive than the EdU proliferation assay, which would only capture instances of fraction of cells in S-phase, but cannot provide quantitative division rates. Moreover, we are currently unable to produce new mice for this study to inject de novo with EdU. However, to investigate this question we analyzed our existing lineage tracing samples via Ki67 staining instead, followed by quantification in n=3 mice per lineage. This data demonstrates once again that the Dlx1 marked population is less proliferative than the Aspm marked population (Figure 2C). Thus, not only the scRNAseq but in fact all of our results so far (H2BGFP pulse-chase, clonal lineage tracing, and Ki67 staining) strongly support the conclusion that the Aspm population is generally more proliferative than the Dlx1 population.

If Aspm-labelling marks a specific cell population rather than a cell state, repetitive weekly injections of tamoxifen at high doses would be expected not to increase the % of labelled basal cells.

To be precise, the repeated injections are every day for 5 days, and not weekly.

We do not rule out the 'cell state' theory for the Aspm population. However, it must be noted - as we explained in the discussion - that the states that Aspm and Dlx1 mark at the beginning of the lineage tracing experiment consistently create one non-self-renewing (NSR) and one SR lineage, respectively. Hence the Dlx1+ and Aspm+ cell states we capture by CreER marking must be stable over the 1-year tracing. Otherwise, constant flickering between basal cell states would result in noisy indistinguishable behaviors of basal subsets; it would not uncover distinct population behavior in long-term lineage tracing, which we in fact uncovered here.

Second, all the many lineage tracings we have done to date inducing Cre recombinases expressed in the hair follicle, epidermis, and the vasculature lineage showed that increasing not only TM dose but also the frequency of injections increases the fraction of labeled cells. This is true even for the strongly expressed K14-CreERT2 and Cdh5-CreERT2 drivers. There is some stochastic nature to the Cre induction causing this effect, as any one injection often does not capture all the cells that express the marker. This may have to do with the state of chromatin during cell cycle and the accessibility of the reporter to the Cre at the time of injection, or other hidden factors that we do not currently understand. The case of Aspm is even more special because this population may be constantly evolving; new G2/M ($Aspm^{high}$) progenitors are likely constantly generated in skin during proliferation of the basal stem cells. This would likely be captured with additional TM injections resulting in increased labeling.

Is it not surprising that so many cells express Aspm protein using immunofluorescence (Figure S1C)?
Comparing to the single-cell data, Aspm protein would be expected to correlate to Ki67+ basal cells? A direct protein correlation (Ki67/Aspm) would be information, again to verify the proliferative nature of the Aspm+ cells.

The immunofluorescence (IF) protein expression and the scRNAseq data together are informative, but not only do not report on the same thing but they might have different sensitivity. For many genes we studied so far, we have observed that scRNAseq data only

© 2024. Published by The Company of Biologists under the terms of the Creative Commons Attribution License (https://creativecommons.org/licenses/by/4.0/).

captures the high expressing cells and misses cells with lower expression. The IF and the Aspm-CreER induction data at high TM dose suggest that the Aspm-expressing cells are frequent in the basal layer. In contrast, scRNAseq only detects rare basal cells, namely those that express the highest level of Aspm. Aspm-CreER induction at low TM level (likely capturing only high Aspm-expressing cells) and hence labels a small fraction of basal cells. Moreover, as also described above, according to our lineage tracing data (both FACS -Fig S3A- and microscopy analysis in homeostasis and wound healing – Fig. 2) the high-dose TM labelled cells contain some long-lasting self-renewing cells or stem cells (in addition to TA cells). The low dose TM labelled cells do not contain stem cells because they do not self-renew in the long term. The Aspm IF staining is not high quality enough nor quantitative enough to detect levels accurately, but the lineage tracing data uncovers an enrichment for Ki67+ staining with Aspm-CreER marked cells when compared with the Dlx1-CreER marked cells (Figure 2C).

Lineage tracing and modelling:
The authors compare total clone sizes and basal clone sizes from Dlx1-CreER and Aspm-CreER lines. The observation that the Aspm-derived clones are different from Dlx1-derived clones is very interesting.

Aspm-basal clones never exceed an average of 2 basal cells, and the total clone size expansion is largely due to an increased number of labelled suprabasal cells. The authors argue that Aspm+ cells are neutrally competitive, which would imply stochastic fate choice and equal probabilities of generating a basal progenitor and basal committed cell; in contrast to the clone size compositions that are reported by the authors.

Analyzing the total clone compositions at early tracing time points could serve to understand if fate choices are altered when comparing Dlx1 and Aspm clones. Similarly, double tracing with Tam/Edu (24 hours) could shed light on if the fate choices of Dlx and Asmp daughter cells are indeed stochastic or if Asmp-daughters are skewed toward a specific fate. In other systems, Aspm is suggested to regulate symmetric fate choice.

A search of the literature suggested to us that Aspm can have complex functions that imply more than spindle orientation and symmetric fate choices. We are also intrigued by how Aspm-CreER marked cells are lost overtime and how – if at all - Aspm may control it. However, overall cell fate choices that lead to cell loss only become truly apparent over relatively long chase time. According to live imaging from the Greco lab, basal cell delaminations and division are not always temporally correlated, and division planes are not always stable and determinant of subsequent fates. Contrary to the way the terms have been used in some publications, it is important to note that “symmetric” and “asymmetric” divisions in the context of the polarity of cell division (e.g., Xu et al. , 2012, PLOS One, 7:e49303) are not directly correlated with the changes in BL clone size observed in lineage tracing. As discussed in our paper, some symmetric divisions increase BL clone size, while symmetric divisions correlated with separate delamination (as observed in video microscopy by the Greco laboratory; see Fig. S9) are “neutral” and do not affect clone size on the lineage tracing time-scale. On the other hand, some symmetric divisions decrease clone size if both daughter cells delaminate, and clone size can also be decreased by division-independent delaminations.

As emphasized in the paper, in contrast with video microscopy, lineage tracing only reports on the long time-scale dynamics of the system and, as in previous studies, the conclusion that fate choices are stochastic only refers to time-averages over at least a week. Correlated non-stochastic behavior on the time scale of days (as reported by Cockburn et al) is “invisible” to lineage tracing (e.g., see Fig. S8). As previously discussed by others (Clayton et al, 2007; Klein and Simons, 2011; Blanpain and Simons, 2013), long time-scale non-stochastic choices (e.g., as suggested by Potten et al are excluded by the “scaling behavior” of the clone-size distribution of SR populations at long chase-times (i.e., Eq. 1 in the text) and the hypothesis of stochastic fate choices provides the simplest explanation (Occam’s razor) of this observation. In this paper, we show that a stricter test of stochasticity (on the lineage tracing time-scale) is provided by the clone-size distribution of Eq. 2, which applies to both NSR and SR populations at all chase-times, and show that both the Dlx1-CreER- and Aspm-CreER-marked populations satisfy this prediction (Figs. 5 and S9). We are not aware of any stricter test.

© 2024. Published by The Company of Biologists under the terms of the Creative Commons Attribution License (https://creativecommons.org/licenses/by/4.0/).

To fully address this point, we must better qualify the distinction between 'neutral competition' and 'neutral drift'. 'Neutral competition' means that within a given progenitor population (e.g. the Aspm-CreER marked cells) all cells compete equally to each other, or in other words they have equal overall potential TO EACH OTHER (not equal probability to self-renew and differentiate). The fate choice of generating basal progenitor (e.g. self-renew) vs basal committed cell (e.g. differentiate) are always stochastic for both self-renewing (SR) and non-self-renewing (NSR) populations, but the probabilities of undergoing these choices are not always equal. In fact, these probabilities are only equal for a SR population [e.g., the total IFE which averages over all populations (Clayton et al, 2007) or those marked by K14-CreER^{lowTM} (Mascre et al, 2012) and our Dlx1-CreER^{lowTM} marked cells]; when the probabilities are equal then the neutral competing population undergoes neutral drift in long-term. In contrast, the probability of making a basal committed cell is generally high for a non-self-renewing (NSR populations) like those marked by Inv-CreER- (Mascre et al, 2012) and Aspm-CreER (this work) and exceed the probability of self-renewal; that is why they lose cells over time and are 'NSR'. The members of an NSR population compete equally with each other (e.g. neutral competition), but do not undergo neutral drift; hence their lineage is lost over time. We have tried again to explain this concept further in the text, and we hope that this attempt will clear out the confusion.

The argument that there are "old" and "new" TA cells in the in vivo epidermis is hard to experimentally verify with the data at hand, and as such this part of the modelling becomes largely speculative.

We agree with this reviewer to modify the terms 'young' and 'old' for the TA cells, but we emphasize that there is clearly an early phase and a late phase for TA cells, and a relatively sharp transition between them. This type of progenitor behavior that changes over time (it is not constant, like that of other populations described to date) cannot be modeled by the previously published neutral drift approach, which is a simplistic subset of the birth-death theory in which population behavior is constant over time.
To implement a generalized birth-death model to lineage tracing data required extensive effort by our experienced theoretical biophysicist (Dr. Shalloway). This approach can now be applied to any progenitor population whose behavior changes over time in any tissue. The work also makes the important distinction that for populations whose behavior is not constant over time, lineage tracing data does not directly report on the biological behavior of the population in homeostasis; this dependency is related with the labeling efficiency of cell progeny at different stages of their evolution in the lineage. In fact, modeling is absolutely required to deconvolve the true biological behavior of the nascent cell as it enters the lineage from the experimental lineage tracing data. This modeling approach will broadly benefit other developmental biology groups that attempt to quantitatively model progenitor populations with phased behavior, and we believe is one of the most important contributions of our study.

With all above said, we do agree with this reviewer that the term 'young' and 'old' to name TA cells on the early vs late phases of their lifespan may be too speculative and somewhat misleading. Hence, we have modified the text to introduce new terms that are more neutral now calling the NSR progeny in the early phase 'pre-transition' TA cells and those in the late phase 'post-transition'. In addition, we eliminated the term 'maturation', which implies that the nascent cell introduced in the lineage become 'old' over time. We now use the term 'clonal age' (i.e., the time after introduction of the nascent cell in the lineage) instead of 'maturation' to avoid speculation about the nature of the change in progeny cell behavior over time.

Figure 2 could display Aspm-clones and not Dlx1-clones. Illustrating the shape of the Aspm-clones would be informative and highlight their suprabasal contribution.

Figure 2 has now become Figure 3. We have now added examples of clones from both Dlx1 and Aspm lineage tracing, and indeed the shape of the clones is interesting and revealing, as anticipated by this reviewer. Thank you for your suggestion.

© 2024. Published by The Company of Biologists under the terms of the Creative Commons Attribution License (https://creativecommons.org/licenses/by/4.0/).

The graphs (Figure 2D-E as well as similar graphs in supplement) are also somewhat miss leading when all clones are displayed without normalization. Average (basal, suprabasal) clone sizes (as in Figure 3) would be more illustrative and allow for statistical comparison between animal cohorts.

The graphs in these figures simply show the extent of the raw data, which is subsequently normalized when the same data is plotted in Figs. 4 and S6. Showing the raw, unnormalized data simply graphically illustrates the numbers of cells and clones that were counted.

As now explained in Appendix I, we only quantify BL clones and not sBL or total clones because there was too much ambiguity in the assignment of sBL cells to clones: The total number of Aspm-CreER^{highTM} "colonies" was larger at day 7 than at day 3.5 and continued to increase until day 30, after which time sBL cells were being lost by epithelial shedding. This was due to an apparent increase in the number of sBL "colonies" above the number of BL clones at day 3.5. Since there are no new labeled clones, this implies that the grouping of sBL cells into sBL clones and/or the grouping of sBL and BL cells into total clones could not be done accurately. Presumably this was because of rapid horizontal migration of differentiated cells in the sBL (suprabasal clone splitting) as has previously been observed (Ro and Rannala, Exper. Dermatol. (2005) 14:838-843) and was also reported in some of the live imaging studies from the Greco laboratory.

The Dlx1-clone size data from 7 days seems to be missing (2D-E).

An abnormally low number of clones were counted in some of the Dlx1-CreER mouse images from day 7, probably because of a problem with tamoxifen injection in these mice. Therefore, we excluded the cell and clone fraction data at this datapoint, since it was closely flanked by the data at days 3.5 and day 14. The ratio between scale and interscale BL clones or mean clone sizes are not affected by the TM issue, so this data was still useable. The raw data (bee-swarm plots) and interscale/scale ratio data were included in old Fig. S2 (now S4) but the mean clone size data was inadvertently excluded. It is now included in the revised Figs. 4C, 5B, S5E, and S10A, and the day 7 raw data is included in Fig. 3. We also added text to the to the appendix to clarify this point.

The clonal analysis shows that almost all clones are lost after 365 days of tracing (Figure 3), data which is not recapitulated by the FACS analysis in Figure S1, where the % of labelled tail skin only drops from 7.8 (1 day) to 4.7 (1 year). The discrepancy between the two forms of analysis is concerning and would need to be clarified.

This is a good point that was previously confusing and insufficiently developed in our early manuscript. We have now added data and clarified in text that high dose TM (2x and 5x injections) in the Aspm-CreER mice capture some self-renewing basal cells that persist in long term (e.g. some stem cells that likely express low levels of Aspm); this is apparent in both lineage tracing (new Figure 2) and in FACS (new Figure S4A, old S1). These stem cells are likely not labelled at low dose TM, when we only mark a subset of cells (likely those with the highest Aspm expression) that do not self-renew in long term, our TA population (Figs 3-5).

The clonal data also suggest that Aspm-derived clones are different depending on if they localize to scale or interscale (Figure S3). How would this impact the modelling which takes all these clones into account? Can the authors uncouple the effect on location from cell intrinsic properties?

While there is a modest preference of the Aspm-derived clones for interscale relative to scale, the clonal dynamics in both locations were essentially the same. As detailed in Fig. S5 (old figure S3), after adjusting for the overall difference in localization, the p-values testing the similarity of the dynamics of total cell fraction, basal cell fraction, clone survival, and mean clone size are all at least 0.5, indicating that there are *no* significant differences. So, analyzing the clones in scale and interscale separately would give the same result. However, splitting the data would cause the error bars to be larger, particularly at long chase times when there are few clones. We previously already concluded in discussion

© 2024. Published by The Company of Biologists under the terms of the Creative Commons Attribution License (https://creativecommons.org/licenses/by/4.0/).

that the scale- vs inter-scale environment does not strongly influence the behavior of our two populations. We emphasize these points and data more in text, to avoid confusion.

The wounding model is interesting, but the design of the experiment leaves more to ask: The authors suggest that both Dlx1-derived and Aspm-derived cells contribute to wound healing. The quantifications are done by quantifying the reporter+ area in the wound, which would likely correlate or at least reflect also to the starting labelling efficiency of the two lines. This data would need to be included to be able to interpret the result.

We normalized the data to the original labeling efficiency, as requested by the reviewer and analyzed groups of n=4 mice for statistical analysis. While our overall qualitative conclusion that both populations contribute to wound healing remained, now both the low TM tail data and the back skin data show that the NSR Aspm-CreER marked population contributes less than the control SR population to wounding.

Furthermore, it would be interesting to characterize the basal contribution of Dlx1 and Aspm in wound healing. During epidermal homeostasis, Aspm-derived progeny is almost exclusively suprabasal – is this pattern changed upon wounding? The timeline of tail wounding should be extended to see if Aspm-derived cells indeed undergo an “extinction phase” as suggested by the authors.

It is hard to make any conclusions of the back skin wounding model, since characterization of Aspm+ cells during homeostasis is only demonstrated in the tail. In addition, similar back skin wounding experiments in the Aspm-line are already published from the group (Kang, Stem Cell Reports, 2020).

N=2 is too little to allow for statistical analysis and would need to be expanded.

To address the question of long-term contribution to wound healing in tail skin, we would need to generate new mice that should be TM induced at ~ 1 month of age and chased for 6-12 months after wounding. We would also need a large cohort of mice for proper statistical analysis of contributions over time, as the wound is tiny on tail skin (4mm) and very few clones exist in this small-wounded-area, when we induced with low TM to capture the TA cells. This is a good question in principle, because if the NSR lineage is not extinguished in wounds, it would imply some sort of de-differentiation relative to their homeostatic state. However, addressing it appropriately is quite challenging and we are unfortunately unable to perform these experiments as part of this revision.

While we have not characterized in detail the Aspm population in back skin during homeostasis (and hence we moved the back skin data to the supplement to de-emphasize it), we do see that wounds (and surrounding areas) gradually lose labeling in this body area over the several weeks chase with n=4 mice/time point, 3 time points and two genotypes. This data was quantified, normalized to the early time point and shows statistical differences; it is quite suggestive, as this is not the case for the known K14-marked SR population in the same region. The wound healing data in back skin that the reviewer cites (Kang et al 2020) only chased the wounds for 1-week and examined effects of demethylase inhibitors on the contributions of the K14—marked SR population. That study never reported the decreased contribution of the Aspm population over time when compared with the K14-population, which is very pertinent to this current study. We also believe that it is a useful for the scientific community to know this behavior of the Aspm-CreER population in back skin wound healing, so we keep this data in the study for readers’ reference; however, we moved it to supplementary figures to de-emphasize it.

Previously, another NSR population (the Involucrin-CreER population) was shown to not contribute to wound healing (Mascre et al 2012). It is interesting to know that our Aspm-CreER NSR population does contribute to some extent, albeit less strongly than the SR population. We focus our conclusion on this narrower and better supported point and hope that the reviewer agrees that the data still makes a valuable contribution to the field and the study.

© 2024. Published by The Company of Biologists under the terms of the Creative Commons Attribution License (https://creativecommons.org/licenses/by/4.0/).

***** Reviewer 2 Advance Summary and Potential Significance to Field:
The authors have been able to identify the elusive epidermal self-renewing transit-amplifying progenitor population in vivo and the interesting finding from this study was the biphasic behavior exhibited by the TA progenitor population by using genetic lineage tracing and mathematical modeling.

Reviewer 2 Comments for the Author:
Overall, the article was well-written though it might be best if the authors avoided extensive abbreviations; for example, BL could be written as basal layer and TD as terminal differentiation. The abbreviations are distracting and would make it difficult for a non-skin scientist to understand.

Because most figure have the abbreviations TD and BL and it'd be difficult to fit entire words in, it'd be best for the reader to remember these abbreviations. We do realize that there are these and other abbreviations, and that it can be distracting. We provide a short list of abbreviation now placed at the beginning of the study and we will work with the editor to find the best place for it in the paper.

The authors demonstrate that the Aspm population was highly proliferative with more than 90% of the cells in S/G2/M in the mouse skin (Fig 1E). On the other hand, in Fig S1B the immunofluorescence staining of human skin with Ki67 and Aspm does not demonstrate a significant colocalization of the two proteins. An image with a higher resolution might be more convincing.

The immunofluorescence (IF) protein expression and the scRNAseq data together are informative, but not only do not report on the same thing but they might have different sensitivity. For many genes we studied so far, we have observed that scRNAseq data only captures the high expressing cells and misses cells with lower expression. The IF and the Aspm-CreER induction data at high TM dose suggest that the Aspm-expressing cells are frequent in the basal layer. In contrast, scRNAseq only detects rare basal cells, namely those that express the highest level of Aspm. Aspm-CreER induction at low TM level (likely capturing only high Aspm-expressing cells) and hence labels a small fraction of basal cells. Moreover, as also described above according to our lineage tracing data (both FACS -Fig S4A- and microscopy analysis in homeostasis and wound healing – Fig. 2) the high-dose TM labelled cells contain some long-lasting cells (in addition to TA cells), while the low dose TM labelled cells do not contain stem cells because they do not self-renew in the long term. The IF staining is not quantitative enough to detect levels accurately, but the lineage tracing data uncovers a good correlation of Ki67+ staining with Aspm-CreER marked cells when compared with the Dlx1-CreER marked cells (Figure 2C).

Fig 2D and E is a quantification of tdTomato+ clones in Aspm-CreER and Dlx1-CreER lineage traced mice. There seems to be a datapoint missing for the Dlx1-CreER clones on day 7.

An abnormally low number of clones were counted in some of the Dlx1-CreER mouse images from day 7, probably because of a problem with tamoxifen injection in these mice. Therefore, we excluded the cell and clone fraction data at this datapoint, since it was closely flanked by the data at days 3.5 and day 14. The ratio between scale and interscale BL clones or mean clone sizes are not affected by the TM issue, so this data was still useable. The raw data (bee-swarm plots) and interscale/scale ratio data were included in old Fig. S2 (now S4) but the mean clone size data was inadvertently excluded. It is now included in the revised Figs. 4C, 5B, S5E, and S10A, and the day 7 raw data is included in Fig. 3. We also added text to the to the appendix to clarify this point.

The switch between proliferation to extinction phase is highly interesting, but the authors could have delved a little more into the mechanism behind the switch.
For Fig. S1H the authors did perform flow cytometry to assess the percentage of tdTomato+ population from Aspm-CreER, they could have flow sorted that fraction of cells from different timepoints to assess the transcriptomic or epigenetic changes that occur during the shift from proliferation to extinction.

© 2024. Published by The Company of Biologists under the terms of the Creative Commons Attribution License (https://creativecommons.org/licenses/by/4.0/).

The difficulty with analyzing data before and after transition is that at high TM dose the labeling is persistent in long-term, suggesting that in addition to TA cells we also capture some SCs. This is the case for the FACS data referenced above. Only at low (clonal) TM dose do we selectively label the TA cells in the basal layer. (This point was unclear in the previous manuscript, and we now add more data and text to clarify how different doses on TM result in different labeling outcomes.) Working with this small fraction of labeled cells at low TM to elucidate mechanisms is not trivial. Furthermore, even if we quickly determined the RNAseq profiles at different stages, to provide in vivo validation confirming such data and to extract the correct interpretation of the mechanisms – which would likely be complex - would require many more years of intense effort. Given this study spanned almost a decade to acquire and analyze this extensive lineage tracing data for several populations until one finally turned out to be a TA population and to develop a new mathematical apparatus that has broad applicability to lineage tracing data of any time of progenitor types (not only the self-renewing ones that can be modeled by neutral drift), we must leave this endeavor for the next study and hope this reviewer will agree with us.

***** Reviewer 3 Advance Summary and Potential Significance to Field:
The manuscript by Ghuwalewala et al aims to understand the biphasic behavior of Transit-amplifying progenitor which contributes to epidermal renewal. Here, the authors use multiple lineage tracing experiments with mathematical modelling and single -cell RNA sequencing to identify the TA progenitor with biphasic behavior. By using the clonal analysis and modelling authors identified the non-self-renewing biphasic TA progenitor which might be important to understand their contribution in tissue repair and homeostasis.
The manuscript is clearly written, and the data well presented. The experiments appear to be well conducted. Overall, the results are nicely explained and discussed thoroughly with appropriate literature form the field. This manuscript will appeal the broad readership of Development. Below are few minor comments to further improve the quality of the paper.

Reviewer 3 Comments for the Author:

Several aspects that might improve the manuscript:
1.The authors showed that "Aspm+ cells were highly enriched in proliferative Ki67 cells". It will be nice to show the other markers which are specifically expressed in the Aspm+ cluster and explain how these markers are different and important in functions apart from all cell cycle G2-M markers.

We have now regressed the cell cycle genes and provide extensive additional analysis and characterization of the Aspm population in new Figure 1 and new Figures S1 and S2 that address this reviewers' point. We also provide all differentially expressed gene list in excel files in the supplementary data.

2.The authors mentioned that "Aspm-CreER marks a novel biphasic NSR population that initially divides rapidly in an early growth phase before a subsequent transition to an extinction phase". It would be interesting to discuss about the mechanism behind this biphasic behavior.

We provide some speculation about the mechanisms of fate transition in Discussion.

3.In Figure 4, authors showed the Wounding response from SR and non-SR populations in tail vs back skin and how these Aspm and K14 derived cells behave differently in tissue repair. It is important to understand the contribution of basal and suprabasal cells in wounding response specifically from Aspm derived cells. Is there any specificity in contribution in tissue repair for basal vs suprabasal cells form Aspm lineage?

Unfortunately, our wounding samples where imaged as whole mount tissue and were not embedded for sectioning and are by now no longer usable for imaging. Furthermore, even with new samples quantitatively estimating contributions to basal vs suprabasal cells in the tinny wounded tail area based on the few clones produced by TM at clonal dose would be extremely challenging. To gather enough data, we would require a large cohort of new mice from both lineages and a major effort for the next 6 or possibly more months.

© 2024. Published by The Company of Biologists under the terms of the Creative Commons Attribution License (https://creativecommons.org/licenses/by/4.0/).

While we agree that the wounding data could have gone in more depth, we hope that the reviewer agrees with us that it still makes a valuable contribution to the field and to our study, even in its current revised form. Previously, another NSR population (the Involucrin-CreER population) did not contribute to wound healing (Mascre et al 2012). It is interesting to know that our Aspm-CreER NSR population does contribute to some extent, albeit less strongly than our SR control population, both in tail and in back skin.

Second decision letter

MS ID#: DEVELOP/2023/202389

MS TITLE: A Transit-amplifying progenitor with biphasic behavior contributes to epidermal renewal

AUTHORS: Sangeeta Ghuwalewala, Kevin Jiang, Sara Ragi, David Shalloway, and Tudorita Tumbar

I have now received all the referees reports on the above manuscript. The referees' comments are appended below, or you can access them online: please go to BenchPress and click on the 'Manuscripts with Decisions' queue in the Author Area.

I am delighted to say that the overall evaluation is positive and we would like to publish a revised manuscript in Development, provided that the referee 1 minor additional comments can be satisfactorily addressed.

Please attend to the reviewers' comments in your revised manuscript and detail them in your point-by-point response. If you do not agree with their suggestion explain clearly why this is so. If it would be helpful, you are welcome to contact us to discuss your revision.

Reviewer 1

Advance summary and potential significance to field

The authors have revised the manuscript and addressed several of the concerns raised.
I appreciate the effort further characterize the Aspm+ cells using available transcriptional data and to include the regressed sc-data. Including images of the Aspm-traced clones is also very illustrative.

Comments for the author

I have two comments on the new data:
1.Figure S2C: how does the “fraction of Aspm+ cells”-graph after regression correlate to the violin-plot displaying Aspm-expression in different clusters? Only cluster 7 seem to have significant Aspm-expression?

2.In the new wounding data (Figure 2E-F) the authors use high and low doses of tamoxifen and perform wound healing assays. If I understand correctly, the argument is that a low dose of tam induces recombination in cells expressing high levels of Dlx/Aspm, whereas a high dose of tamoxifen would ALSO (not preferentially) be able to recombine cells with low(er) gene/CreER expression.

If so, a high dose of tamoxifen would be expected to recombine all Dlx-high cells (SR) targeted using a low dose, and in addition target all Dlx-low expressing cells (NSR), potentially giving rise to a diluted Dlx-high regenerative effect. This is perhaps what the difference in contribution comparing Dlx-cells with high and low tamoxifen represents? The same argument would then be true for the Aspm-positive population. Looking at the results (now nicely normalized to starting labelling efficiency), it seems that changing the dose of tamoxifen does not alter the regenerative capacity of the Aspm-population, suggesting that the same (NSR) population/cell state is labelled in

© 2024. Published by The Company of Biologists under the terms of the Creative Commons Attribution License (https://creativecommons.org/licenses/by/4.0/).

both experiments. Which is the opposite to the conclusion made by the authors, and also suggested by the lineage tracing/FACS quantification. It would be good if the authors could clarify this point.

Reviewer 2

Advance summary and potential significance to field

The authors have made substantial revisions to the manuscript and have satisfactorily addressed all the questions raised by this reviewer.

Comments for the author

None

Reviewer 3

Advance summary and potential significance to field

This paper defined the heterogeneity of stem cells and progenitors in the skin using elegant lineage tracing approaches

Comments for the author

The manuscript by Ghuwalewala et al has addressed all the concerns with new experiments and analysis and thoughtful responses to the comments.

The authors have performed an extensive analysis of single-cell data and characterization of the Aspm population to show how Aspm+Ki67+
And Aspm+Ki67- cells are different. They have discussed probable mechanisms of fate transition and mentioned how the Aspm lineage contributes to wound healing.

I recommend this article for publication in Development.

Second revision

Author response to reviewers' comments

Response to Additional Points of Reviewer 1:

Reviewer 1 Advance summary and potential significance to field
The authors have revised the manuscript and addressed several of the concerns raised.
I appreciate the effort further characterize the Aspm+ cells using available transcriptional data and to include the regressed sc-data. Including images of the Aspm-traced clones is also very illustrative.

Reviewer 1 Comments for the Author:
I have two comments on the new data:
1. Figure S2C: how does the “fraction of Aspm+ cells”-graph after regression correlate to the violin-plot displaying Aspm-expression in different clusters? Only cluster 7 seem to have significant Aspm-expression?

© 2024. Published by The Company of Biologists under the terms of the Creative Commons Attribution License (https://creativecommons.org/licenses/by/4.0/).

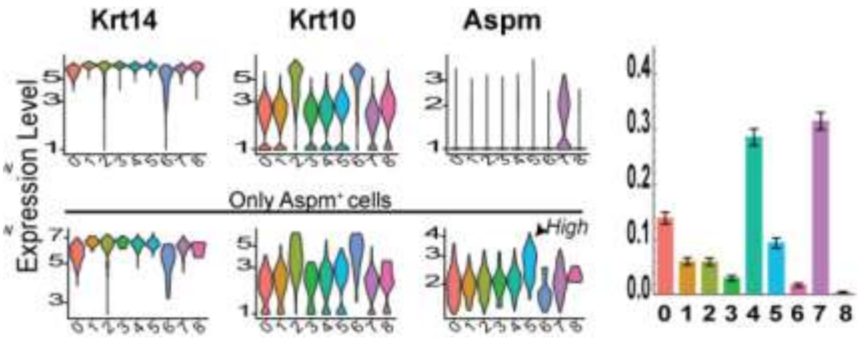

In Figure S2C (see panel above) The upper panels show violin plots that include both Aspm+ and Aspm- cells; most clusters have a relatively low fraction of Aspm+ cells and cluster 7 is the one with most (~30%) Aspm+ cells. Cluster 4 also has ~30% Aspm+ cells, but the level is relatively low, hence the violin plot is nearly absent in the top panel graphs. We suspect the reviewer might have missed the lower panel graphs labelled "only Aspm+ cells", which show violin plots for expression including ONLY Aspm+ cells. In this representation you can see that for instance cluster 5, although it contains only ~10% Aspm+ cells (right bar graph) it contains the highest Aspm expressing cells (arrowhead and the word 'High' indicate that in the lower panel on the violin plot. We added a sentence to figure S2C legend to avoid this confusion as follows.

Fig S2 (C) UMAP visualization of the Sca1+/α6-integrin+ basal layer (BL) datasets before and after regression (left). Cluster cell type annotations are denoted by color and number in the legend. Violin plots of the indicated gene expression levels in the clusters and bar charts of the fractions of Aspm+ cells/cluster are shown. Note lower panel graphs showing violin plots of the Aspm+ subset from each cluster.

2.In the new wounding data (Figure 2E-F) the authors use high and low doses of tamoxifen and perform wound healing assays. If I understand correctly, the argument is that a low dose of tam induces recombination in cells expressing high levels of Dlx/Aspm, whereas a high dose of tamoxifen would ALSO (not preferentially) be able to recombine cells with low(er) gene/CreER expression.

We indeed believe that this is likely the best explanation for differences between high vs low tamoxifen data.

If so, a high dose of tamoxifen would be expected to recombine all Dlx-high cells (SR) targeted using a low dose, and in addition target all Dlx-low expressing cells (NSR), potentially giving rise to a diluted Dlx-high regenerative effect. This is perhaps what the difference in contribution comparing Dlx-cells with high and low tamoxifen represents?

This is a good observation and a good explanation for the Dlx lineage behavior. It is important to realize that because wound healing occurs over a 3-5 week period, the putative Dlx-low NSR population captured at high TM hypothesized by this reviewer, must be very short-lived (less than 3-weeks). This NSR could be the K10+ basal differentiating, which lives only ~1-2 weeks in basal layer, according to data from Greco lab. Their disappearance over the 3-5 wound healing period would affect the lineage tracing data in wound healing. Notably, at least ~10% of Dlx+ basal cells are K10+ by scRNAseq (Fig. 1B).

The same argument would then be true for the Aspm-positive population. Looking at the results (now nicely normalized to starting labelling efficiency), it seems that changing the dose of tamoxifen does not alter the regenerative capacity of the Aspm-population, suggesting that the same (NSR) population/cell state is labelled in both experiments. Which is the opposite to the conclusion made by the authors, and also suggested by the lineage tracing/FACS quantification. It would be good if the authors could clarify this point.

Indeed, the long-term lineage tracing and FACS quantification in homeostasis indicate clearly that in the Aspm-CreER lineage low TM marks a TA population while high TM captures some stem cells. This conclusion is mostly revealed in long-term chases. There is still a difference between high TM

© 2024. Published by The Company of Biologists under the terms of the Creative Commons Attribution License (https://creativecommons.org/licenses/by/4.0/).

and low TM at the 3-5 weeks period of wound healing but is small. This could be due to the short chase period, the small number of SCs captured at high TM dose in the Aspm lineage, or the labeling of K10+ basal progenitors, that balance out the SC contributions, further diminishing the difference between high dose and low dose Aspm. It is also formally possible that the SCs captured by high TM Aspm are not the same as the Dlx low TM SCs, and that their contribution to wound healing is different.

Third decision letter

MS ID#: DEVELOP/2023/202389

MS TITLE: A Transit-amplifying progenitor with biphasic behavior contributes to epidermal renewal

AUTHORS: Sangeeta Ghuwalewala, Kevin Jiang, Sara Ragi, David Shalloway, and Tudorita Tumbar

I am happy to tell you that your manuscript has been accepted for publication in Development, pending our standard ethics checks.

© 2024. Published by The Company of Biologists under the terms of the Creative Commons Attribution License (https://creativecommons.org/licenses/by/4.0/).
